# Supplementary material for: Re-assessing thermal response of schistosomiasis transmission risk: Evidence for a higher thermal optimum than previously predicted
Source: PLoS Negl Trop Dis. 2024 Jun 10;18(6):e0011836. doi: 10.1371/journal.pntd.0011836 (PMC11207148; doi:10.1371/journal.pntd.0011836)
Supplement: S1 Supplementary materials — Fig A: Fecundity rate of snails and confidence intervals Biomphalaria Spp. Fig B: Fecundity rate of snails and confidence interval Bulinus Spp. Fig C: Mortality rate of snails and confidence interval Biomphalaria Spp. Fig D: Mortality rate of snails and confidence interval Bulinus Spp. Fig E: Prepatent period of snails and confidence interval Biomphalaria Spp. Fig F: Prepatent period of snails and confidence interval Bulinus Spp. Fig G: Probability of hatching success of miracidia and confidence interval S. mansoni. Fig H: Cercariae release rate and confidence interval Biomphalaria Spp. Fig I: Mortality rate of miracidia and confidence interval S. mansoni. Fig J: Mortality rate of miracidia and confidence interval S. haematobium. Fig K: Mortality rate of cercaria and confidence interval S. mansoni. Fig L: Mortality infected snails and confidence interval Biomphalaria Spp. Fig M: Mortality infected snails and confidence interval Bulinus Spp. Fig N: Transmission rate in snails and confidence interval Biomphalaria Spp. Fig O: Transmission rate in snails and confidence interval Bulinus Spp. Fig P: Transmission rate in humans and confidence interval Biomphalaria Spp. Fig Q: Simulation of basic reproduction number for S. mansoni (left) and S. haematobium (right). Fig R: Quantile regression of prevalence over R0 estimates for our estimate and for Nguyen et al. estimate [27]. Fig S: Blue and red color show when the water contact rate is non constant function of temperature and black color shows when the water contact rate is constant and equals to 1 for all temperature. Table A: The parameters’ description. Table B: The selected curves for S. mansoni and Biomphalaria species. Table C: The selected curves for S. haematobium and Bulinus species. (DOCX) [file pntd.0011836.s001.docx]

THE SUPPLEMENTARY SOURCE OF THERMAL SENSITIVE MECHANISTIC MODEL FOR SCHISTOSOMIASIS

Ibrahim Aslan and Giulio De Leo

Stanford, January 24, 2024

Contents

[The mechanistic model 2](#_Toc161320883)

[Parameters 4](#_Toc161320884)

[Constant parameters 4](#_Toc161320885)

[The factor of density-dependent fecundity rate of snails 4](#_Toc161320886)

[The reduction factor of the fecundity rate due to infection 4](#_Toc161320887)

[The maximum rate of snail invasion 5](#_Toc161320888)

[Constant human population having schistosomiasis 5](#_Toc161320889)

[The number of eggs produced by a mature worm 5](#_Toc161320890)

[The maturation rate of the worms in the human body 5](#_Toc161320891)

[The mortality rate of worms due to human death 5](#_Toc161320892)

[The mortality rate of worms 5](#_Toc161320893)

[Nonconstant parameters 5](#_Toc161320894)

[The fecundity rate of snails 7](#_Toc161320895)

[The mortality rate of snails 13](#_Toc161320896)

[The prepatent period in snails 19](#_Toc161320897)

[The probability of hatching success of miracidia 26](#_Toc161320898)

[The number of cercarial released per snail 28](#_Toc161320899)

[The mortality rate of miracidia 31](#_Toc161320900)

[The mortality rate of cercaria 34](#_Toc161320901)

[The mortality rate of infected snails 36](#_Toc161320902)

[The transmission rate of schistosomiasis in snails 43](#_Toc161320903)

[The transmission rate of schistosomiasis in humans 49](#_Toc161320904)

[Basic reproduction number 53](#_Toc161320905)

[The calculation of basic reproduction number 53](#_Toc161320906)

[Model comparison with data 56](#_Toc161320907)

[GNTD prevalence data and temperature 56](#_Toc161320908)

[Basic reproduction number as a predictor of prevalence 56](#_Toc161320909)

[Masking for non-suitable regions for schistosomiasis 57](#_Toc161320910)

[Human water contact rate 58](#_Toc161320911)

[Sensitivity 58](#_Toc161320912)

[The derivative of each thermal performance function 59](#_Toc161320913)

[Gaussian 59](#_Toc161320914)

[Spain 59](#_Toc161320915)

[Filnn 59](#_Toc161320916)

[Thomas 59](#_Toc161320917)

[Quadratic 60](#_Toc161320918)

[Briere2 60](#_Toc161320919)

[Johnson Lewin 60](#_Toc161320920)

[The partial derivative of each parameter 60](#_Toc161320921)

[Transmission rate in human $\boldsymbol{\beta hT}$ 60](#_Toc161320922)

[Transmission rate in snail $\boldsymbol{\beta sT}$ 60](#_Toc161320923)

[Miracidia hatching rate $\boldsymbol{\delta eT}$ 61](#_Toc161320924)

[Cercarial releasing rate $\boldsymbol{\nu cT}$ 61](#_Toc161320925)

[Prepatent period $\boldsymbol{\sigma sT}$ 61](#_Toc161320926)

[Mortality rate of miracidia, $\boldsymbol{\mu mT}$ 61](#_Toc161320927)

[Mortality rate of cercaria, $\boldsymbol{\mu cT}$ 62](#_Toc161320928)

[Mortality rate of infected snails, $\boldsymbol{\mu iT}$ 62](#_Toc161320929)

[References 62](#_Toc161320930)

# The mechanistic model

The model consisted of five compartments: susceptible snails $(S$); prepatent snails, infected by *miracidia* but not shedding *cercariae* yet, which is also the stage of developing sporocysts ($P$); infectious snails, shedding *cercariae* ($I$); mean number of immature worms that do not produce eggs ($W$); and mean number of mature worms that produce eggs ($W_{m}$).

$$\frac{dS}{dt}=\left( \nu_{s}\left( T \right)-\left( S+P+I \right)\nu\right)\left( S+rP \right)-\lambda\left( T \right)S-\mu\left( T \right)S$$

$$\frac{dP}{dt}=\lambda\left( T \right)S-\left( \sigma_{s}\left( T \right)+\mu_{i}\left( T \right) \right)P$$

$$\frac{dI}{dt}=\sigma_{s}\left( T \right)P-\mu_{i}\left( T \right)I$$

$$\frac{dW}{dt}=C_{w}\beta_{h}\left( T \right)C^{*}-\sigma_{p}W$$

$$\frac{dW_{m}}{dt}=\sigma_{p}W-\left( \mu_{h}+\mu_{p} \right)W_{m}$$

where the force of infection

$$\lambda\left( T \right)=\Lambda\left( 1-e^{-\beta_{s}\left( T \right)\frac{M^{*}}{N}} \right)=\Lambda\left( 1-e^{-\beta_{s}\left( T \right)\frac{h\delta_{e}\left( T \right)\nu_{e}W_{m}/\mu_{m}\left( T \right)}{S+P+I}} \right)$$

Note that the explicit format of dynamic consists of seven compartments. We embed two fast dynamics (miracidia (*C*); and cercariae (*M*) life span) into slow dynamics by setting the system into the equilibrium points.

$$\frac{dM}{dt}=h\nu_{e}\delta_{e}\left( T \right)W_{m}-\mu_{m}\left( T \right)M=0\Rightarrow M^{*}=\frac{h\nu_{e}\delta_{e}\left( T \right)W_{m}}{\mu_{m}\left( T \right)}$$

$$\frac{dC}{dt}=\nu_{c}\left( T \right)I-\mu_{c}\left( T \right)C=0\Rightarrow C^{*}=\frac{\nu_{c}\left( T \right)}{\mu_{c}\left( T \right)}I$$

Note that some portions of miracidia and cercariae are transmitted to snails' tissue and the human body. These portions are neglected due to their magnitude compared to the mortality rate of miracidia and cercaria.

Table A: The parameters' description

| Parameter | Description | Unit |
| --- | --- | --- |
| $\beta_{s}$*(T)* | Transmission rate of schistosomiasis in snails | $s/p$ |
| $\beta_{h}$*(T)* | Transmission rate of schistosomiasis in humans | *1/*$h*d$ |
| $v_{s}$*(T)* | Fecundity rate of snails | *1/*$d$ |
| $\mu(T)$ | Mortality rate of snails | *1/*$d$ |
| $\delta_{e}$*(T)* | Probability of hatching success of miracidia | *p/*$e$ |
| $\mu_{m}$*(T)* | Mortality rate of miracidia | [*1/*$d$](https://www.codecogs.com/eqnedit.php?latex=1%2Fd#0) |
| $\mu_{c}$*(T)* | Mortality rate of cercariae | [*1/*$d$](https://www.codecogs.com/eqnedit.php?latex=1%2Fd#0) |
| $\sigma_{s}$*(T)* | Prepatent period in snails | [*1/*$d$](https://www.codecogs.com/eqnedit.php?latex=1%2Fd#0) |
| $\mu_{i}$*(T)* | Mortality rate of infected snails | [*1/*$d$](https://www.codecogs.com/eqnedit.php?latex=1%2Fd#0) |
| $v_{c}$*(T)* | Number of cercariae released by a snail per day | [*p/s*d*](https://www.codecogs.com/eqnedit.php?latex=p%2Fs*d#0) |
| $v$ | Factor of density-dependent fecundity rate | [*1/s*d*](https://www.codecogs.com/eqnedit.php?latex=1%2Fs*d#0) |
| $r$ | Reduction factor of fecundity rate due to infection | *-* |
| $v_{e}$ | Number of eggs produced by a mature worm | [*e/p*d*](https://www.codecogs.com/eqnedit.php?latex=e%2Fp*d#0) |
| $\Lambda$ | Maximum rate of snail invasion | [*1/d*](https://www.codecogs.com/eqnedit.php?latex=1%2Fd#0) |
| $\sigma_{p}$ | Maturation rate of worms in human body | [*1/d*](https://www.codecogs.com/eqnedit.php?latex=1%2Fd#0) |
| $h$ | Constant human population having worms | *h* |
| $\mu_{p}$ | Mortality rate of worms | *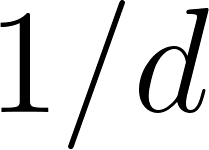* |
| $\mu_{h}$ | Mortality rate of worms due to human death | *1/d* |
| $C_{w}$ | Water contact rate | *-* |

# Parameters

The mechanistic model has two sets of parameters. The constant parameters do not vary with temperature and the non-constant parameters vary with temperature.

## Constant parameters

In this section, we discuss the parameter values not varying with temperature. The list of these parameters: Factor of density-dependent fecundity rate, Reduction factor of fecundity rate due to infection, Number of eggs produced by a mature worm, Maximum rate of snail invasion, Maturation rate of worms in the human body, Constant human population having worms, Mortality rate of worms, Mortality rate of worms due to human death.

### The factor of density-dependent fecundity rate of snails

The parameter is denoted in the model with $\nu$and has the unit of 1/(snail*day). The parameter represents the rate of contribution to reach the carrying capacity per snail in one day if the disease-free equilibrium (DFE) is stable. For instance, if we assume DFE is stable and the carrying capacity is 10000 then, $\nu=1/10000$. However, since the endemic equilibrium is stable in most areas of our interest [1], we adjust this value to get a desirable snail population size.

### The reduction factor of the fecundity rate due to infection

The parameter is denoted in the model with $r$ and does not have a unit. It represents the drop in the fecundity rate due to disease in the infected snails. The values should vary between [0,1]. There are not many resources, but one the study by [2] takes the fecundity rate of infected snails to be a little less than half of the susceptible snails, so we can assume it drops the fecundity rate by half. In addition, a study [3] uses the value $r= 0.5$.

### The maximum rate of snail invasion

The parameter is denoted in the model with $\Lambda$ and has a unit of 1/day. It regulates the transmission rate of disease in snails [4]. Thus, we manipulate the value of $\Lambda$ to reach the desirable prevalence in snails.

### Constant human population having schistosomiasis

The parameter is denoted in the model with $h$and has the unit of human. The parameter represents the constant human population in the territory of interest. This value varies across geography.

### The number of eggs produced by a mature worm

The parameter is denoted in the model with $\nu_{e}$ and has the unit of egg/(parasite*day). The value of the parameter represents the eggs released into the environment by one pair of worms in one day. One study in [5] mentions “*S.* *mansoni* worm pairs laid approximately 350 eggs per day with no change in the apparent rate of egg-laying between eight and 52 weeks after infection, and approximately one-third of the eggs were passed in the feces”. [6] mentions “a mated pair of schistosomes is responsible for 200–300 ~ 350/3 eggs per day”. We assume this value does not depend upon temperature because the eggs are produced inside the human body.

### The maturation rate of the worms in the human body

The parameter is denoted in the model with $\sigma_{p}$ and has a unit of 1/day. The value of the parameter represents the maturation rate of worms per day. The average time for maturation of *S.mansoni* is 50-60 days according to [6]. One other resource by [2] uses $\sigma_{p}= 0.02$ for the maturation rate of worms in the human body for species *S.* *haematobium* and *S.* *mansoni.* We assume this value does not depend upon temperature because worms’ maturation is happening inside the human body.

### The mortality rate of worms due to human death

The parameter is denoted in the model with $\mu_{h}$and has a unit of 1/day. The value of the parameter represents the death rate of humans per day.

### The mortality rate of worms

The parameter is denoted in the model with $\mu_{p}$ and has a unit of 1/day. It represents the death rate of worms per day. Since parasites live in the human body and humans are endothermic species, this value does not change with the outside temperature. A study by [7] found the *S. mansoni* worm's life span with a 95% confidence interval to be [5.7-10.5] ~ 8 years. Thus, we assume the average lifespan of a worm in the human body is 8 years.

## Nonconstant parameters

In this section, we discuss the temperature-varying rates from the experiment results that are found in the literature for different species and then use these rates with the R package called rTPC [8] to find a curve for each temperature-dependent parameter. The package performs 24 thermal-sensitive curves for each temperature-dependent parameter. We pick the curve based on three criteria; i) the smallest Akaike information criteria (AIC) score, ii) simpler regarding the number of parameters and iii) biologically applicable. The curves are performed for *S. mansoni* and *S.* *haematobium* parasites separately and for *Biomphalaria* and *Bulinus* snails separately. In addition, in order to analyze the uncertainty in our curve estimation, we did bootstrap resampling and found a 95 percent confidence interval around each curve. The following two tables give the selected models and their details (which model the selected to be used, the AIC ranking of the selected model, the optimal temperature of the selected model, bootstrapping method to obtain the confident interval, explicit formulation of selected model) for *S. mansoni-Biomphalaria* system and *S. haematobium-Bulinus* system. The rest of the section extensively discusses the details about each selected curve.

Table B: The selected curves for S. mansoni and Biomphalaria species

| **The parameter** | **The selected model** | **The (AIC) ranking of the model** | **The optimal temp** | **Bootstrapping method** | **Representation** |
| --- | --- | --- | --- | --- | --- |
| Fecundity rate of snail | Gaussian | 1 | 24 °C | Resampling | $r_{max}e^{-0.5\left( \frac{T-T_{opt}}{a} \right)^{2}}$ |
| Mortality rate of snail | Spain | 1 | 21 °C | Resampling | $r_{0}e^{\mathrm{aT}}\left( 1-be^{\mathrm{cT}} \right)$ |
| Mortality rate of inf. Snail | Flinn | 8 | Increasing function | Residual resampling | $\frac{1}{1+a+bT+cT^{2}}$ |
| Mortality rate of miracidia | Thomas 2017 | 1 | 13.5 °C | Resampling | $ae^{bT}-\left( c+de^{eT} \right)$ |
| Mortality rate of cercaria | Spain | 2 | 16.5 °C | Residual resampling | $r_{0}e^{\mathrm{aT}}\left( 1-be^{\mathrm{cT}} \right)$ |
| Hatching rate of miracidia | Flinn | 1 | Increasing function | Resampling | $\frac{1}{1+a+bT+cT^{2}}$ |
| Prepatent period in snail | Irf | 1 | Increasing function | Resampling |  |
| Cercaria release rate | Gaussian | 1 | 27.5 °C | Resampling | $r_{max}e^{-0.5\left( \frac{T-T_{opt}}{a} \right)^{2}}$ |
| The transition rate in snails | Spain | 12 | 33 °C | Resampling | $r_{0}e^{\mathrm{aT}}\left( 1-be^{\mathrm{cT}} \right)$ |
| The transition rate in humans | Briere2 | 8 | 24 °C | Resampling | $aT\left( T-T_{min} \right)\left( T_{max}-T \right)^{\frac{1}{b}}$ |

Table C: The selected curves for S. haematobium and Bulinus species

| **The parameter** | **The selected model** | **The (AIC) ranking of model** | **The optimal temp** | **Bootstrapping method** | **Representation** |
| --- | --- | --- | --- | --- | --- |
| Fecundity rate of snail | Johnson Lewin | 14 (simpler) | 27.8 °C | Resampling |  |
| Mortality rate of snail | Quadratic | 8 | 15 °C | Resampling | $a+bT+cT^{2}$ |
| Mortality rate of inf. Snail | Spain | 3 | Increasing function | Residual resampling | $r_{0}e^{\mathrm{aT}}\left( 1-be^{\mathrm{cT}} \right)$ |
| Mortality rate of miracidia | Spain | 2 | 22.5 °C | no enough data to do resampling | $r_{0}e^{\mathrm{aT}}\left( 1-be^{\mathrm{cT}} \right)$ |
| Mortality rate of cercaria | Spain | 2 | 16.5 °C | Residual resampling | $r_{0}e^{\mathrm{aT}}\left( 1-be^{\mathrm{cT}} \right)$ |
| Hatching rate of miracidia | Flinn | 1 | Increasing function | Resampling | $\frac{1}{1+a+bT+cT^{2}}$ |
| Prepatent period in snail | Irf | 1 | Decaying function | Resampling |  |
| Cercaria release rate | Gaussian | 1 | 27.5 °C | Resampling | $r_{max}e^{-0.5\left( \frac{T-T_{opt}}{a} \right)^{2}}$ |
| The transition rate in snails | Flinn | 8 | 30.5 °C | Residual resampling | $\frac{1}{1+a+bT+cT^{2}}$ |
| The transition rate in humans | Briere2 | 8 | 24 °C | Resampling | [$\mathrm{aT}\left( T-T_{\min} \right)\left( T_{\max}-T \right)^{\frac{1}{b}}$](https://www.codecogs.com/eqnedit.php?latex=aT(T-T_%7Bmin%7D)(T_%7Bmax%7D-T)%5E%5Cfrac%7B1%7D%7Bb%7D#0) |

### The fecundity rate of snails

The parameter is denoted in the model with $\nu_{s}$has the unit of 1/day. The value represents reproduction of snails in a day. We use Euler- Lotka equation to approximate the fecundity rate [9].

$$1 = \sum_{a}^{w} \lambda^{-a}l(a)b(a)$$

where $\lambda$ is the growth rate, *l*(a) is the fraction of individuals surviving to age *a,* and *b(a)* is the number of offspring born to an individual of age during the time step. $w$ is the longest time to survive. By utilizing the model above

1= $\sum_{a}^{w} \left( growth rate \right)^{-\left( a+age of maturity \right)}*\left( daily surv. \right)^{a}*0.5*\left( \frac{\frac{egg}{snail}}{day} \right)*\left( prob. of surv. till maturity \right)$

We can draw the growth rate from the formula above and by using fecundity-mortality = growth rate, thus the fecundity rate is mortality + growth. We will be using: Age at maturity, hatching rate, Daily Survival, and Egg/snail/day given in empirical studies to estimate the fecundity rate of snails.

*Biomphalaria sudanica* [10]. In this table, *Biomphalaria alexandrina* data are used from [11] for the age of maturity and hatching rates.

| Temperature | Age at maturity | Hatching rate | Daily survival rate | Egg/snail/day | Fecundity rate |
| --- | --- | --- | --- | --- | --- |
| 13.4°C | 9999 | 0 | 0.990596 | 0.11714 | 0 |
| 15.7°C | 360 | 89.5 | 0.9831741 | 3.71714 | 0 |
| 16.7°C | 107 | 89.5 | 0.994776 | 6.48286 | ​​0.0349 |
| 18.9°C | 107 | 89.5 | 0.9929975 | 8.52714 | 0.0369 |
| 20.9°C | 107 | 97.9 | 0.9984211 | 8.63857 | 0.0395 |
| 22.8°C | 107 | 97.9 | 0.9910489 | 7.00000 | 0.0386 |
| 26.7°C | 77 | 98.8 | 0.9926992 | 7.03857 | 0.0499 |
| 28.3°C | 77 | 98.8 | 0.977706 | 5.11429 | 0 |
| 29.5°C | 154 | 97.7 | 0.9610102 | 2.96286 | 0 |
| 32.0°C | 360 | 0 | 0.9409337 | 0.58714 | 0 |

*Biomphalaria pfeifferi* [12]*.* The temperature fluctuates in this study. (This study was not used in the simulation due to not constant temperature.)

| Temperature | Age at maturity | Hatching rate | Daily survival rate | Egg/snail/day | Fecundity rate |
| --- | --- | --- | --- | --- | --- |
| 18-28^o^ C | 32 | 98 | 0.99748 | 10.22014 | 0.09774 |
| 20.5-25.5^o^ C | 33 | 99 | 0.99368 | 4.024405 | 0.07891 |
| 23^o^ C | 36 | 99 | 0.99631 | 4.941586 | 0.07686 |

*Biomphalaria pfeifferi* [13].

| Temperature | Age at maturity | Hatching rate | Daily survival rate | Egg/snail/day | Fecundity rate |
| --- | --- | --- | --- | --- | --- |
| 25^o^ C | 42 | 95.6 | 0.99717 | 7.133363 | 0.07058 |
| 27^o^ C | 35 | 88.8 | 0.98991 | 3.652113 | 0.06824 |
| 29^o^ C | N/A | N/A | N/A | 0 | 0 |

*Biomphalaria alexandrina* [11].

| Temperature | Age at maturity | Hatching rate | Daily survival rate | Egg/snail/day | Fecundity rate |
| --- | --- | --- | --- | --- | --- |
| 15^o^ C | 360 | 89.5 | 0.989 | 0.166 | 0.00683 |
| 20^o^ C | 107 | 97.9 | 0.994 | 0.663 | 0.0229 |
| 25^o^ C | 77 | 98.8 | 0.993 | 0.849 | 0.0309 |
| 30^o^ C | 154 | 97.7 | 0.985 | 0.057 | 0.00824 |
| 35^o^ C | 360 | 0 | 0.962 | 0 | 0 |

*Biomphalaria pfeifferi* [14].

| Temperature | Age at maturity | Hatching rate | Daily survival rate | Egg/snail/day | Fecundity rate |
| --- | --- | --- | --- | --- | --- |
| 19^o^ C | 110 | 97.9 | 0.98581 | 1.24541 | 0.02720 |
| 25^o^ C | 40 | 98.9 | 0.98647 | 5.03455 | 0.07378 |
| 30^o^ C | 38 | 97.7 | 0.95977 | 1.990214 | 0.06568 |
| 35^o^ C | N/A | 0 | N/A | 0 | 0 |

*Biomphalaria glabrata* [15].

| Temperature | Age at maturity | Hatching rate | Daily survival rate | Egg/snail/day | Fecundity rate |
| --- | --- | --- | --- | --- | --- |
| 20^o^ C | 112 | 86 | 0.7876 | 5.714 | 0.05856 |
| 25^o^ C | 55 | 96 | 0.9160 | 21.428 | 0.08750 |
| 30^o^ C | 56 | 95 | 0.8439 | 2.857 | 0.06577 |
| 35^o^ C | NA | 0 | NA | 0 | 0 |

*Biomphalaria glabrata* [16]*.* In this table, *Biomphalaria alexandrina* data are used from [11] for the age of maturity and hatching rates.

| Temperature | Age at  maturity | Hatching rate | Daily survival rate | Egg/snail/day | Fecundity rate |
| --- | --- | --- | --- | --- | --- |
| 5^0^ | - | - |  | 0 | 0 |
| 15^0^ | - | - |  | 0 | 0 |
| 20^0^ | 107 | 97.9 | 0. 994 | 2.5/21 = 0.12 | 0.0132 |
| 25^0^ | 77 | 98.8 | 0.993 | (42.2/20+19.2/15+17.1/10+1.7/6)/4=1.35 | 0.0349 |
| 30^0^ | 154 | 97.7 | 0.985 | (0/5+2.5/10+1.3/15+0)/4 =0.0842 | 0.00969 |
| 35^0^ | 360 | 0 | 0.962 | 0 | 0 |

*Biomphalaria pfeiffer* [17]*.* In this table, *Biomphalaria alexandrina* data are used from [11] for hatching rates.

| Temperature | Age at maturity | Hatching rate | Daily survival rate | Egg/snail/day | Fecundity rate |
| --- | --- | --- | --- | --- | --- |
| 18^0^ | 11.5*14 = 161 | 89.5 | 0.998 | 0.33 | 0.0138 |
| 22^0^ | 4.5*14 = 63 | 97.9 | 0.996 | 3.58 | 0.0507 |
| 25^0^ | 4.5*14 = 63 | 98.8 | 0.997 | 0.81 | 0.0349 |
| 27^0^ | 5.5*14 = 77 | 98.8 | 0.995 | 1.521 | 0.036 |

*Bulinus truncates* [11].

| Temperature | Age at maturity | Hatching rate | Daily survival rate | Egg/snail/day | Fecundity rate |
| --- | --- | --- | --- | --- | --- |
| 15^o^ C | 220 | 90.7 | 0.998 | 0.261 | 0.0103 |
| 20^o^ C | 108 | 100 | 0.999 | 0.968 | 0.025 |
| 25^o^ C | 75 | 93.7 | 0.995 | 1.335 | 0.0348 |
| 30^o^ C | 135 | 97.5 | 0.970 | 0.548 | 0.0199 |
| 35^o^ C | 250 | 95.2 | 0.908 | 0.102 | 0 |

*Bulinus globosus* [18]*.* In this table, *Bulinus truncates* data are used from [11] for the age of maturity and hatching rates.

| Temperature | Age at  maturity | Hatching rate | Daily survival rate | Egg/snail/day | Fecundity rate |
| --- | --- | --- | --- | --- | --- |
| 15.5°C | N/A | N/A | 0.998 | 0 | 0 |
| 21.2°C | 108 | 100 | 0.993 | 2.59909 | 0.027 |
| 25.8°C | 75 | 93.7 | 0.989 | 2.766767 | 0.037 |
| 31°C | 135 | 97.5 | 0.979 | 2.08225 | 0.019 |
| 35.5°C | N/A | N/A | 0.955 | 0 | 0 |

*Bulinus globosus* [19]*.* In this table, *Bulinus truncates*  data are used from [11] for the hatching rates.

| Temperature | Age at maturity | Hatching rate | Daily survival rate | Egg/snail/day | Fecundity rate |
| --- | --- | --- | --- | --- | --- |
| 18^0^ | 11.5*14 = 161 | 90.7 | 0.994 | 0.3215143 | 0.0137 |
| 22^0^ | 6.5*14 = 63 | 100 | 0.993 | 2.285877 | 0.0447 |
| 25^0^ | 4.5*14 = 63 | 93.7 | 0.98 | 3.197019 | 0.0497 |
| 27^0^ | 4.5*14 = 63 | 93.7 | 0.98 | 1.159799 | 0.0391 |

*Bulinus nyassanus,* [20]*.* In this table, *Bulinus truncatus* data are used from [11] for the age of maturity rates.

| Temperature | Age at maturity | Hatching rate | Daily survival rate | Egg/snail/day | Fecundity rate |
| --- | --- | --- | --- | --- | --- |
| 22^0^ | 108 | 67.11 | 0.993 | 2.846667 | 0.0297 |
| 25^0^ | 75 | 88.65 | 0.994 | 3.336667 | 0.0427 |
| 28^0^ | 75 | 88.96 | 0.979 | 4.226667 | 0.0463 |
| 31^0^ | 135 | 91.12 | 0.989 | 3.733333 | 0.0451 |

The top five models for *Biomphalaria* snails

| Model | AIC value | BIC |
| --- | --- | --- |
| Gaussian | -176 | -169 |
| Modifiedgaussian | -174 | -166 |
| Oneill | -174 | -166 |
| Pawar | -174 | -166 |
| Sharpeschoolhugh | -174 | -166 |

Gaussian curve is selected and the expression is

$$r_{\max}e^{-0.5\left( \frac{T-T_{\mathrm{opt}}}{a} \right)^{2}}$$

See [21] for additional information. The fitted curve to *Biomphalaria* data as follow

| A  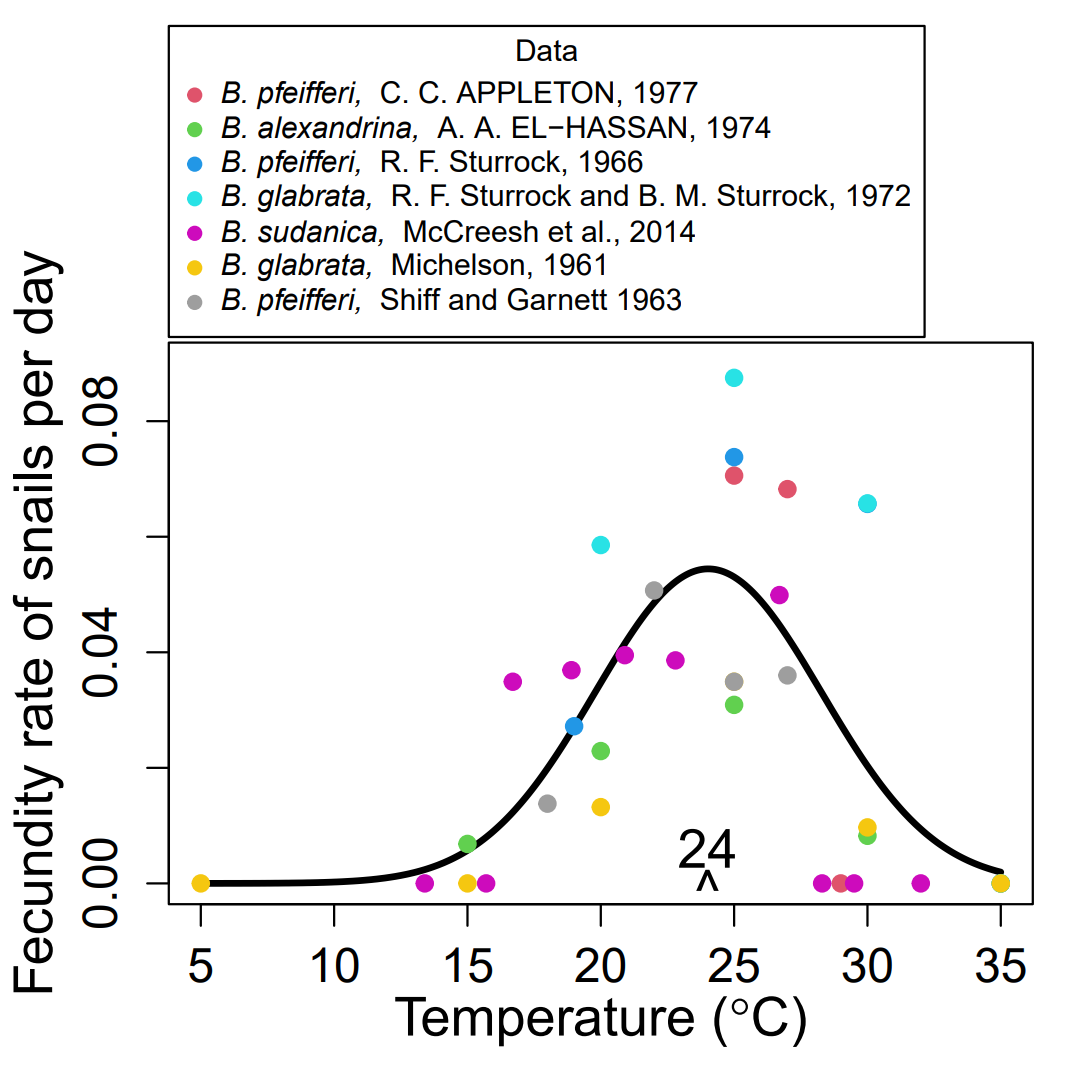 | B  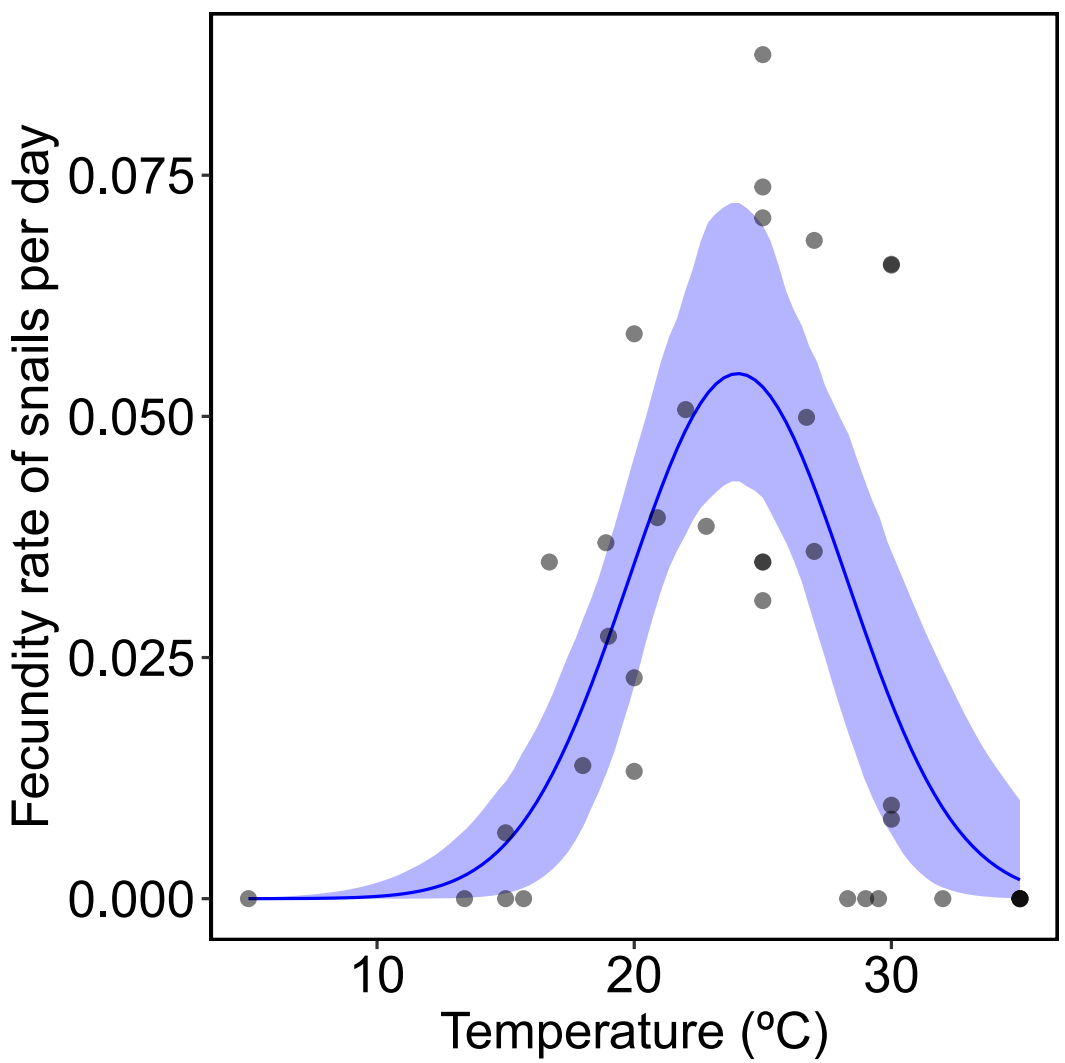 |
| --- | --- |

Fig A: Fecundity rate of snails and confidence intervals Biomphalaria Spp.

The top five models for *Bulinus* snails

| Model | AIC value | BIC |
| --- | --- | --- |
| Ratkowsky | -134 | -129 |
| Modifiedgaussian | -133 | -128 |
| Oneill | -133 | -128 |
| Gaussian | -132 | -128 |
| Weibull | -132 | -127 |
| Johnson Lewin (14th in ranking) | -128 | -123 |

Johnson Lewin curve is selected and the expression is

$$\frac{r_{0}e^{\frac{-e}{k\left( T+273.15 \right)}}}{1+e^{-\frac{e_{h}-\left( \frac{e_{h}}{T_{opt}+273.15}+kln\left( \frac{e}{e_{h}-e} \right) \right).\left( T+273.15 \right)}{k.\left( T+273.15 \right)}}}$$

See Johnson and Lewin [22] for additional information. The fitted curve to *Bulinus* data as follow

| A  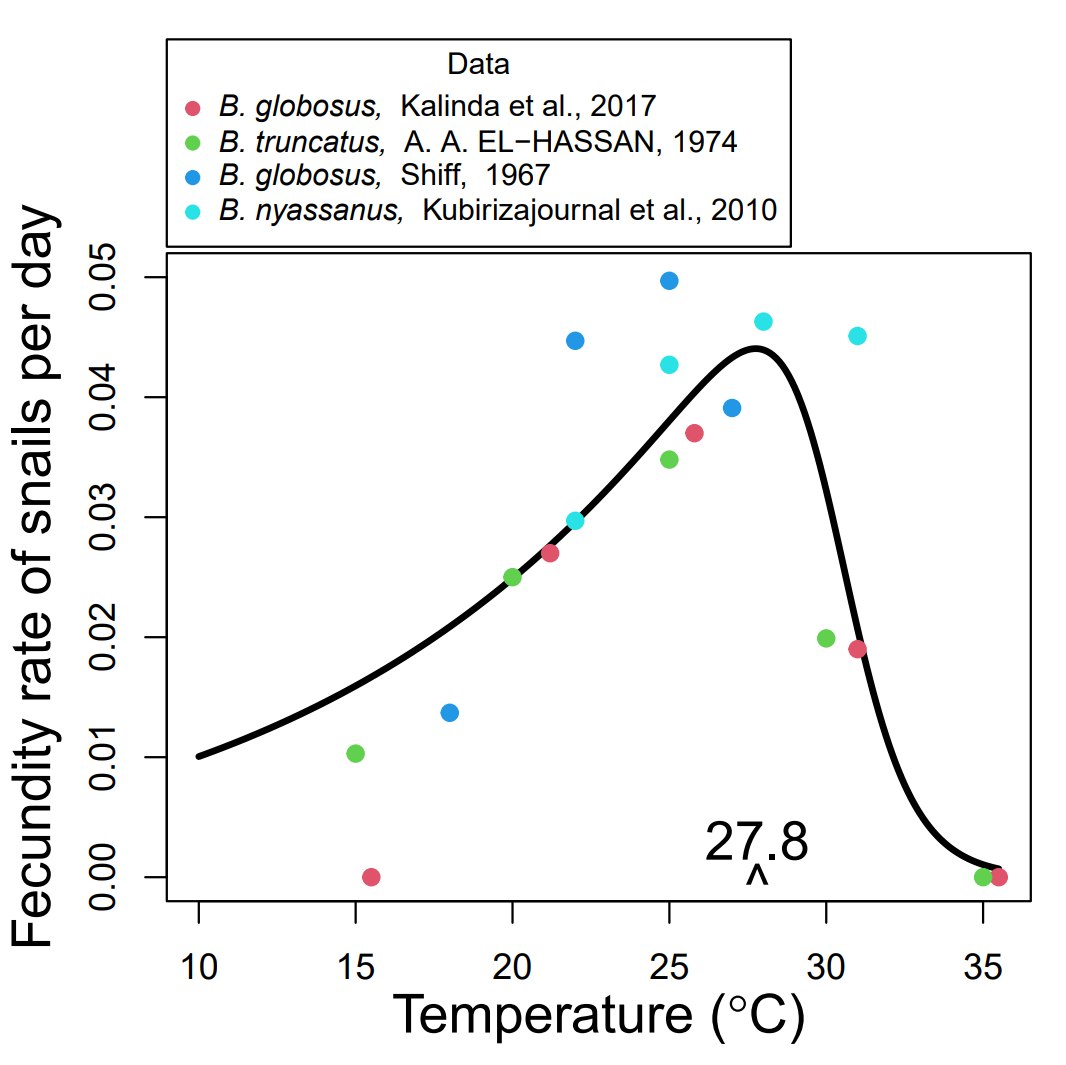 | B  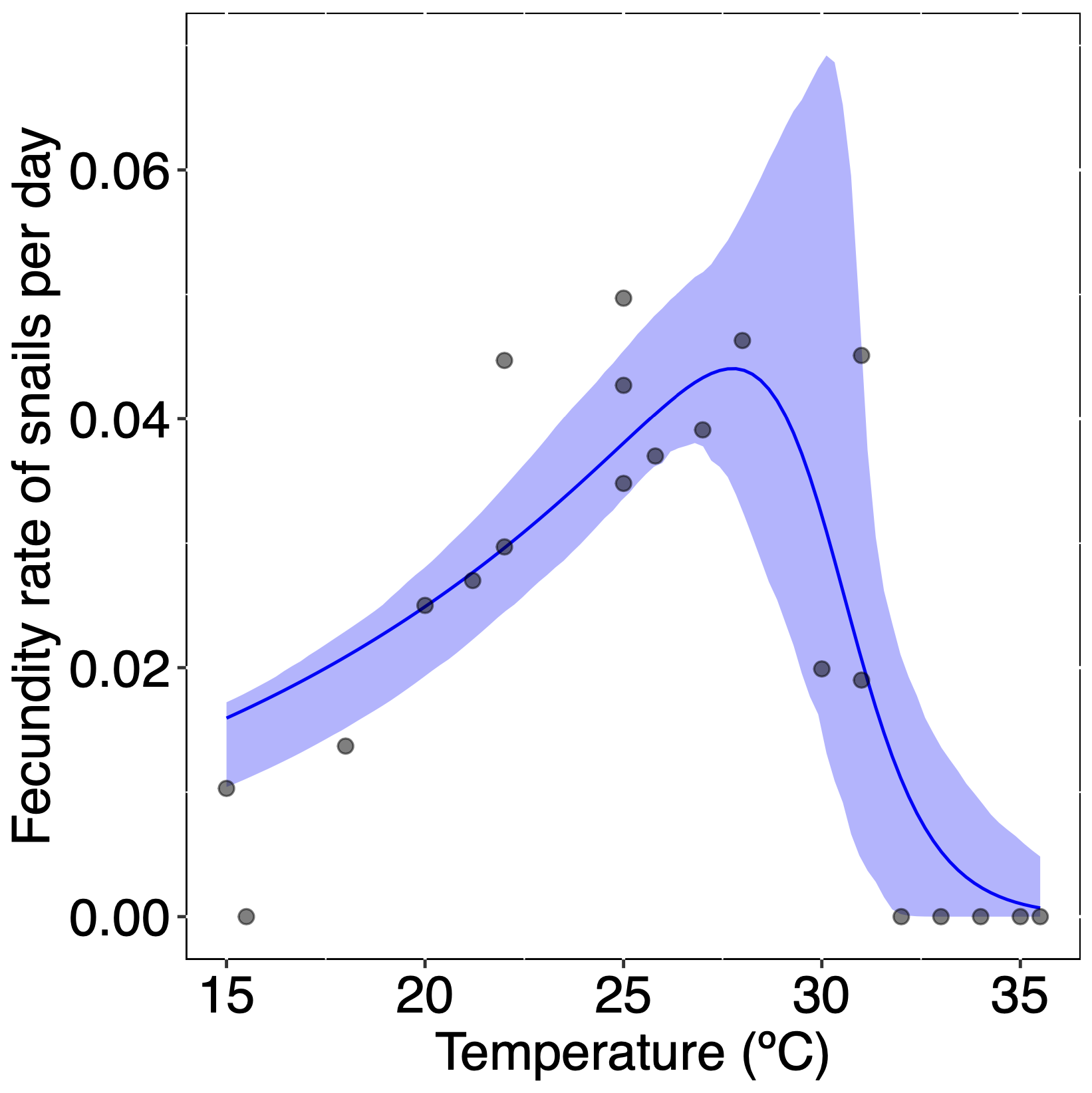 |
| --- | --- |

Fig B: Fecundity rate of snails and confidence interval Bulinus Spp.

### The mortality rate of snails

The parameter is denoted in the mechanistic model with $\mu$has the unit of 1/day. The parameter represents the mortality rate of snails per day. We use the exponential growth model

$$\frac{dN(t)}{dt}= -\mu N(t)\Rightarrow\mu=-\frac{log(N/N_{0})}{t}$$

with given survival portions of snails each time then take the average of the found rates to get the mortality rate. For instance, given the percentage of survivals $\left( x_{1}, ..., x_{n} \right)$ for times $\left( t_{1}, ..., t_{n} \right)$ at each temperature T. We use the formula $\mu_{i}=\frac{log(x_{i})}{t_{i}}$ and then take the average of $\mu$to get the mortality rate for temperature T.

*Biomphalaria pfeifferi* [17]*.*

| Temperature | Mortality rate |
| --- | --- |
| 18^0^ | 0.001650303 |
| 22^0^ | 0.002659904 |
| 25^0^ | 0.002373008 |
| 27^0^ | 0.004170385 |

*Biomphalaria sudanica* [10]*.*

| Temperature | Mortality rate |
| --- | --- |
| 13.4°C | 0.009404046 |
| 15.7°C | 0.01682591 |
| 16.7°C | 0.005224019 |
| 18.9°C | 0.007002545 |
| 20.9°C | 0.001578888 |
| 22.8°C | 0.008951098 |
| 26.7°C | 0.007300832 |
| 28.3°C | 0.02229403 |
| 29.5°C | 0.03898983 |
| 32.0°C | 0.05906629 |

*Biomphalaria pfeifferi* [23]*.*

| Temperature | Mortality rate |
| --- | --- |
| 34°C | 0.1457006 |
| 36°C | 0.6661235 |
| 38°C | 1.649401 |
| 40°C | 5.821733 |

*Biomphalaria pfeifferi* [12]*.* Note that here temperature fluctuates.

| Temperature | Mortality rate |
| --- | --- |
| 23^o^ C, no fluctuation | 0.00369937 |
| 20.5-25.5^o^ C fluctuation | 0.006338469 |
| 18-28^o^ C | 0.002522396 |

*Biomphalaria pfeifferi* [13]*.*

| Temperature | Mortality rate |
| --- | --- |
| 25^o^ C | 0.002832593 |
| 27^o^ C | 0.01014083 |
| 29^o^ C | 0.02164846 |

*Biomphalaria alexandrina* [11]*.*

| Temperature | Mortality rate |
| --- | --- |
| 10^o^ C | 0.01161 |
| 15^o^ C | 0.01077 |
| 20^o^ C | 0.00596 |
| 25^o^ C | 0.00674 |
| 30^o^ C | 0.01505 |
| 35^o^ C | 0.03891 |
| 37^o^ C | Inf |

*Biomphalaria pfeifferi* [14]*.*

| Temperature | Mortality rate |
| --- | --- |
| 19^o^ C | 0.0142923 |
| 25^o^ C | 0.01361983 |
| 30^o^ C | 0.04106602 |

*Biomphalaria pfeifferi* [24]*.*

| Temperature | Mortality rate |
| --- | --- |
| 22.85^o^ C | 0.006674626 |
| 24.01^o^ C | 0.01155245 |
| 26.26^o^ C | 0.0260108 |
| 28.07^o^ C | 0.03678792 |

*Biomphalaria alexandrina* [25]*.*

| Temperature | Mortality rate |
| --- | --- |
| 10^0^ | NAN |
| 18^0^ | 0.006188814 |
| 26^0^ | 0.006188814 |
| 28^0^ | 0.009373412 |
| 33^0^ | NAN |

*Bulinus truncates* [25]*.*

| Temperature | Mortality rate |
| --- | --- |
| 10^0^ | 0.00256859 |
| 18^0^ | 0.004560943 |
| 26^0^ | 0.003184598 |
| 28^0^ | 0.004560943 |
| 33^0^ | NAN |

*Bulinus globosus* [19]*.*

| Temperature | Mortality rate |
| --- | --- |
| 18^0^ | 0.005314696 |
| 22^0^ | 0.006322683 |
| 25^0^ | 0.01045628 |
| 27^0^ | 0.01057945 |

*Bulinus globosus* [18]*.*

| Temperature | Mortality rate |
| --- | --- |
| 15.5°C | 0.00244925 |
| 21.2°C | 0.006901087 |
| 25.8°C | 0.01151302 |
| 31°C | 0.02073716 |
| 35.5°C | 0.04642702 |

*Bulinus nyassanus* [20]*.*

| Temperature | Mortality rate |
| --- | --- |
| 22^0^ | 0.006849573 |
| 25^0^ | 0.00513718 |
| 28^0^ | 0.02054872 |
| 31^0^ | 0.01027436 |

*Bulinus globosus* [23]*.*

| Temperature | Mortality rate |
| --- | --- |
| 34°C | 0.02924029 |
| 36°C | 0.1490773 |
| 38°C | 0.8022027 |
| 40°C | 3.540546 |

*Bulinus africanus* [23]*.*

| Temperature | Mortality rate |
| --- | --- |
| 34°C | 0.1044946 |
| 36°C | 0.5664857 |
| 38°C | 1.618022 |
| 40°C | 4.344277 |

*Bulinus truncates* [11]*.*

| Temperature | Mortality rate |
| --- | --- |
| 10^o^ C | 0.00442 |
| 15^o^ C | 0.00218 |
| 20^o^ C | 0.00144 |
| 25^o^ C | 0.00518 |
| 30^o^ C | 0.03077 |
| 35^o^ C | 0.09622 |
| 37^o^ C | Inf |

The top five models for *Biomphalaria* snails

| Model | AIC value | BIC |
| --- | --- | --- |
| Spain | -223 | -216 |
| Thomas2 | -222 | -213 |
| Joehnk | -219 | -210 |
| Beta | -216 | -207 |
| Ratkowsky | -215 | -208 |

Spain curve is selected and the expression is

$$r_{0}e^{aT}\left( 1-be^{cT} \right)$$

See [26] for additional information. The fitted curve to *Biomphalaria* data as follow

| A  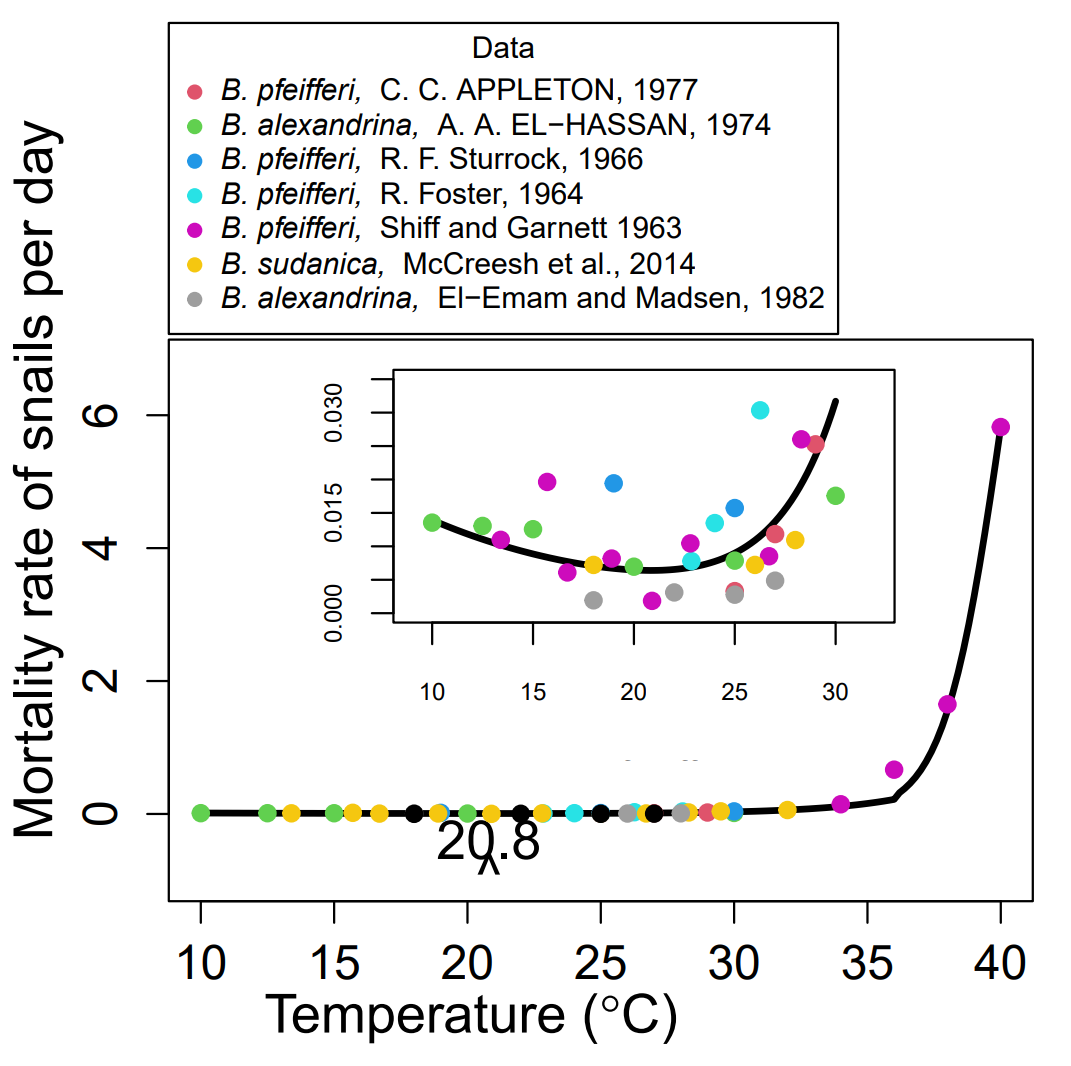 | B  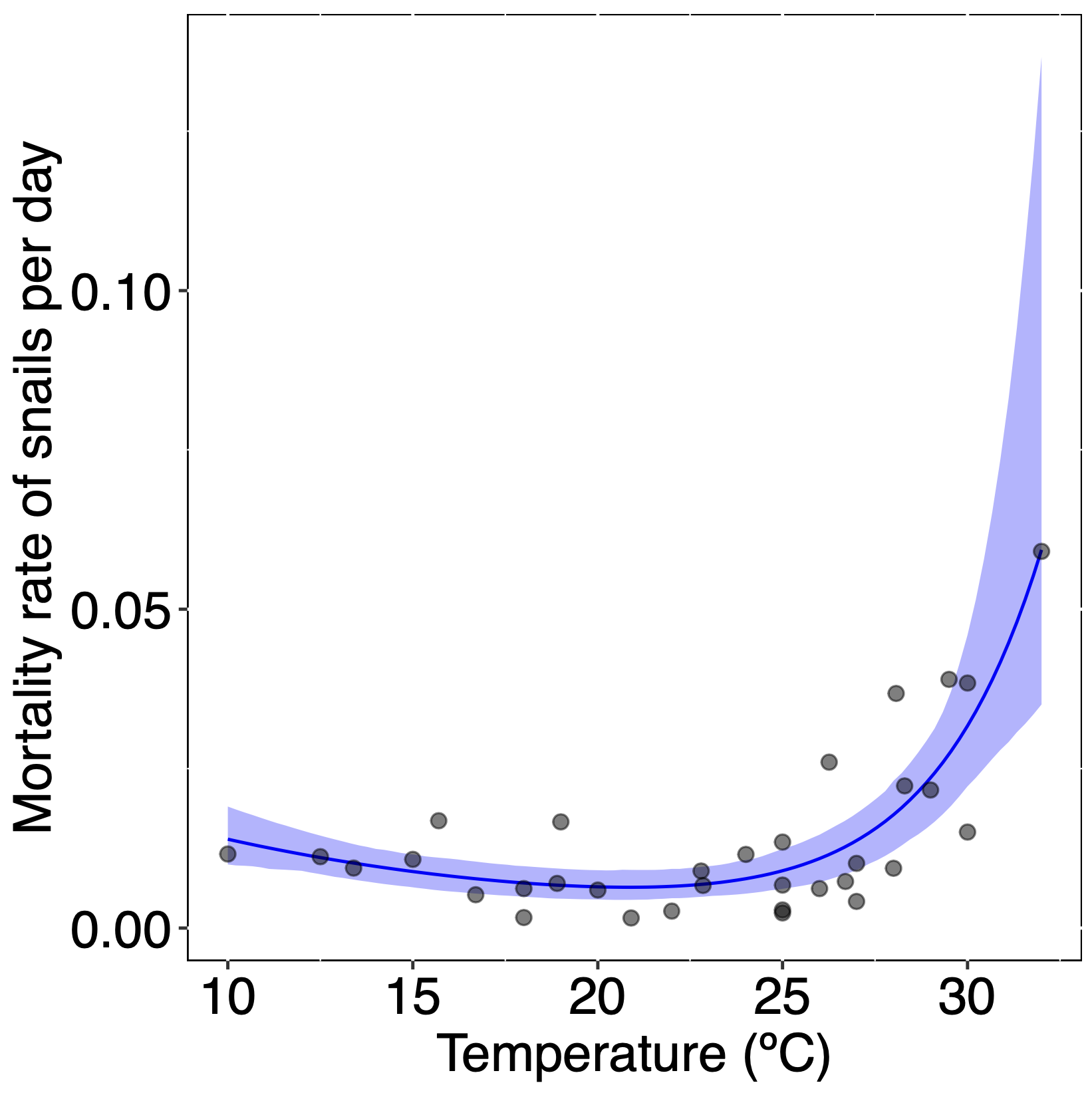 |
| --- | --- |

Fig C: Mortality rate of snails and confidence interval Biomphalaria Spp.

The top five models for *Bulinus* snails

| Model | AIC value | BIC |
| --- | --- | --- |
| Irf | -165 | -160 |
| Modifiedgaussian | -165 | -160 |
| Joehnk | -158 | -158 |
| Spain | -158 | -153 |
| Pawar | -158 | -153 |
| Quadratic (8th in ranking) | -156 | -152 |

Quadratic curve is selected and the expression is

$$a+bT+cT^{2}$$

See [27] for additional information. The fitted curve to *Bulinus* data as follow

| A  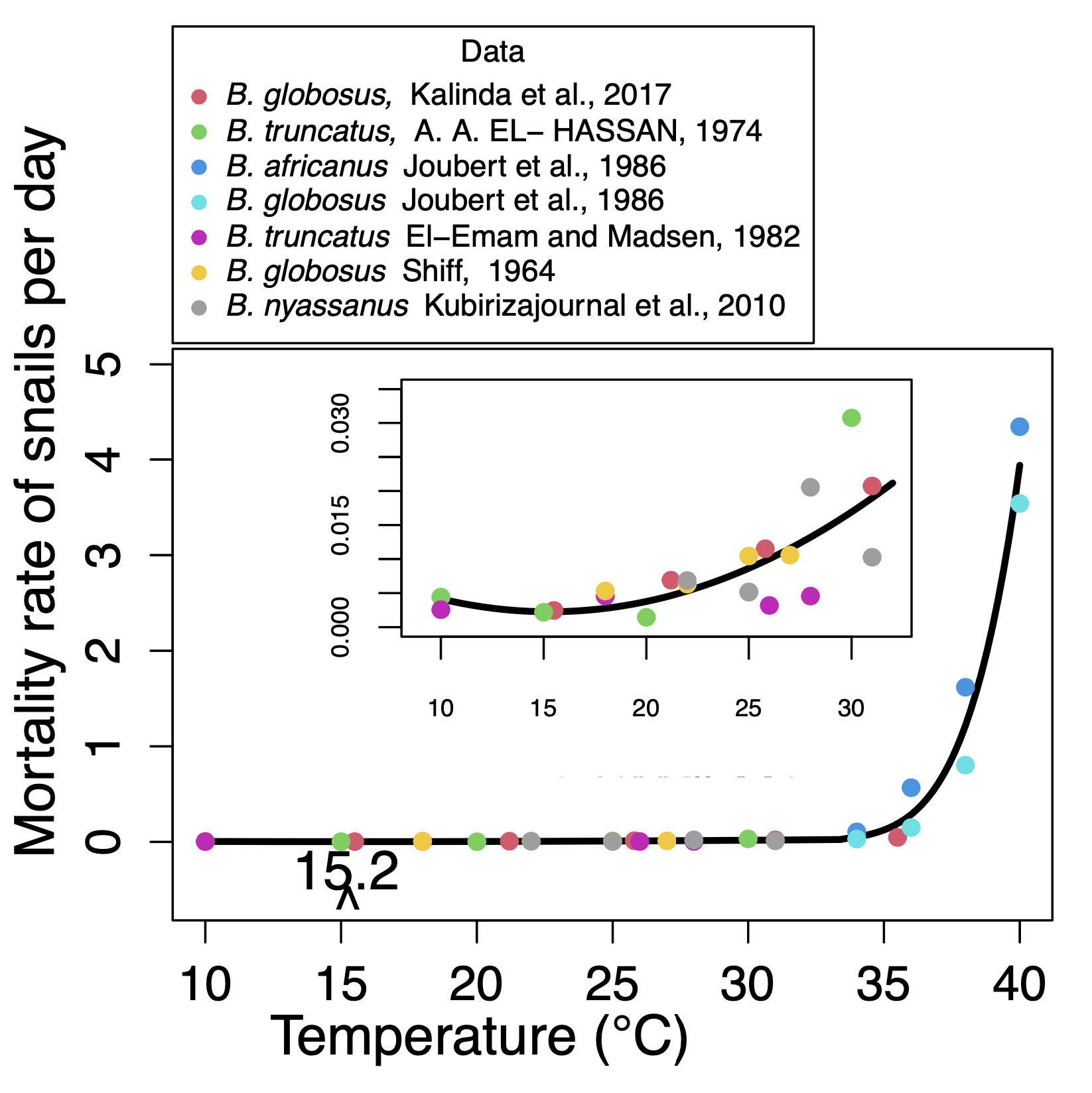 | B  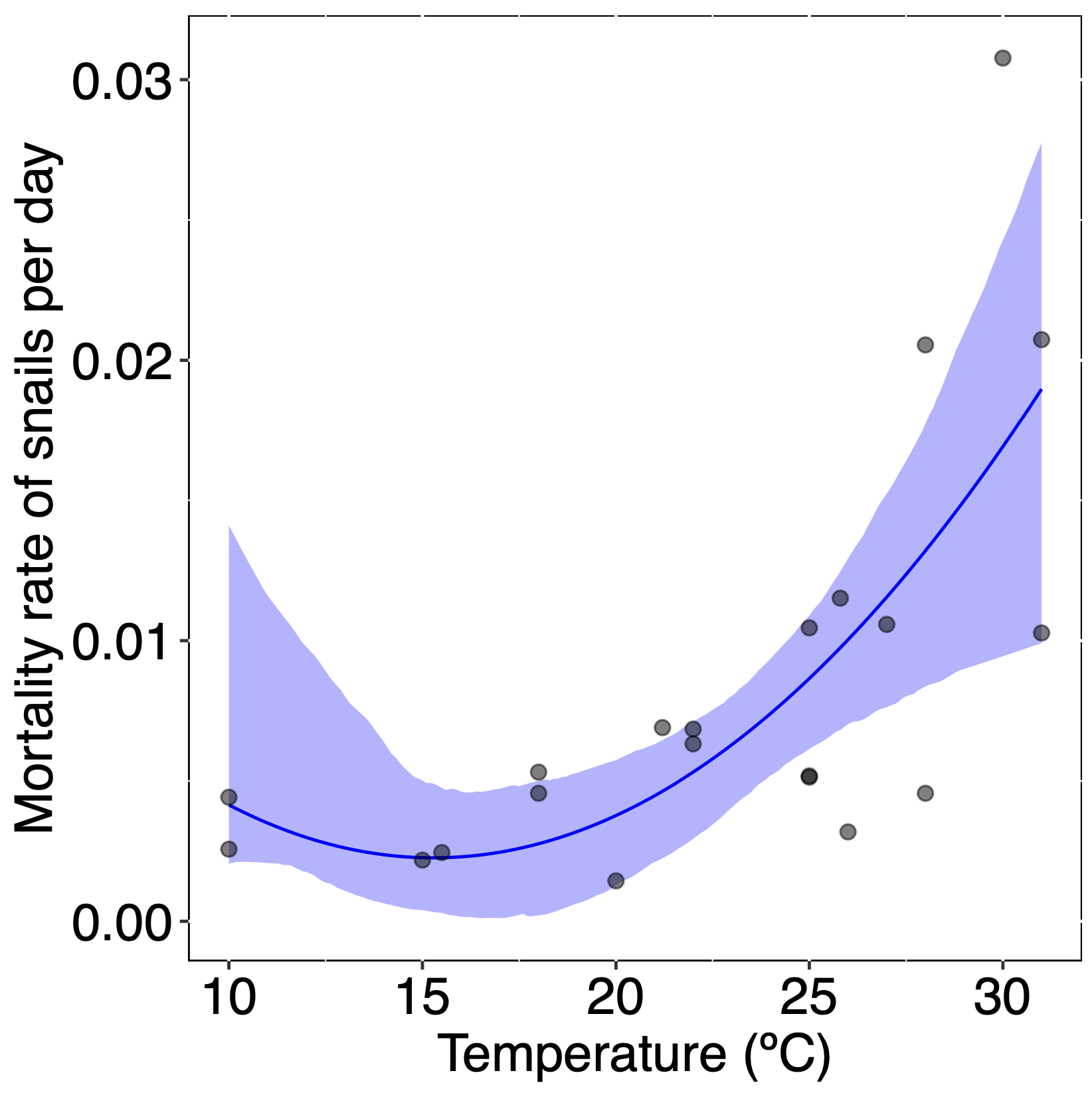 |
| --- | --- |

Fig D: Mortality rate of snails and confidence interval Bulinus Spp..

### The prepatent period in snails

$\sigma_{s}$ has the unit of 1/day and represents the rate of snails moving from exposed class to infectious class per day.

*Biomphalaria glabrata* with *S. mansoni* [28]. Diurnally changing water temperature.

| Temperature | Prepatent period (days) | Average of the prepatent period (days) |
| --- | --- | --- |
| 12 hours 28-19^o^ C, mean 24.3^o^ C | 25-27 | 26 |
| 6 hours 28-19^o^ C, mean 21.9^o^ C | 35-36 | 35.5 |
| 2 hours 28-19^o^ C, mean 20.2^o^ C | 43-45 | 44 |
| 1 hours 28-19^o^ C, mean 19.8^o^ C | 50-51 | 50.5 |
| 23.4 – 11.5^o^ C, mean 17.5^o^ C | 68 | 68 |
| 22.5 – 13.3^o^ C, mean 17.9^o^ C | 57-58 | 57.5 |
| 25.2 – 13.1^o^ C, mean 19.2^o^ C | 52 | 52 |
| 26.9 – 15.2^o^ C, mean 21.1^o^ C | 37 - 39 | 38 |
| 21.5 – 16.4^o^ C, mean 18.9^o^ C | 56-59 | 57.5 |
| 30 – 20^o^ C, mean 25^o^ C | 16-18 | 17 |
| 35.1 – 24.7^0^ C, mean 29.9^o^ C | 16-17 | 16.5 |
| 38.9 – 27.5^o^ C, mean 33^o^ C | 16-17 | 16.5 |

*Biomphalaria glabrata* with *S. mansoni* [29]. Constant temperature.

| Temperature | Prepatent period (days) |
| --- | --- |
| 17^o^ C | 92.5 |
| 19^o^ C | 55.5 |
| 22^o^ C | 34 |
| 25^o^ C | 24.8 |
| 28^o^ C | 19.2 |
| 30^o^ C | 16.3 |
| 31^o^ C | 15.9 |
| 32^o^ C | 14.9 |
| 33^o^ C | 15.3 |
| 34^o^ C | 15.9 |
| 35^o^ C | 16 |

*Biomphalaria pfeifferi* with *S. mansoni* [24]*.*

| Temperature | Prepatent period (days) |
| --- | --- |
| 18^o^ C | 57 |
| 21^o^ C | 37 |
| 22.85^o^ C | 32 |
| 24.01^o^ C | 30 |
| 26.26^o^ C | 23 |
| 28.07^o^ C | 19 |
| 30.04^o^ C | 18 |
| 31.75^o^ C | 16 |

*Planorbis pfeifferi* with *S. mansoni* [30]*.*

| Temperature | Prepatent period (days) |
| --- | --- |
| 21.7^o^ C | 36 |
| 22^o^ C | 33 |
| 26.3^o^ C | 23.5 |
| 26.6^o^ C | 23 |
| 26.9^o^ C | 22.2 |
| 27.3^0^ C | 22 |
| 27.7^o^ C | 19 |
| 31.8^o^ C | 15.4 |
| 32.1^o^ C | 15.8 |
| 32.7^o^ C | 17.2 |
| 32.8^o^ C | 14.7 |
| 35^o^ C | 16.4 |

*Physopsis (bolinus) globosa* with *S. haematobium* [30]*.*

| Temperature | Prepatent period (days) |
| --- | --- |
| 22^o^ C | 67 |
| 26.3^o^ C | 38.5 |
| 26.4^o^ C | 39.3 |
| 26.8^o^ C | 34.8 |
| 31.9^o^ C | 27.5 |
| 32.1^o^ C | 23 |
| 33^o^ C | 25.5 |
| 35.2^o^ C | 26.3 |

*Bulinus truncatus* with *S. haematobium* [31]*.*

| Temperature | Prepatent period (days) | Average of the prepatent period (days) |
| --- | --- | --- |
| 17^o^ C | No infection |  |
| 33^o^ C | No infection |  |
| 18^o^ C | Min 106-113, Max 120-129 | 117 |
| 19^o^ C | Min 75-78, Max 137-151 | 110 |
| 20^o^ C | Min 64-67, Max 91-95 | 79 |
| 21^o^ C | Min 41-56, Max 69-75 | 60 |
| 22^o^ C | Min 44-54, Max 62-69 | 57 |
| 23^o^ C | Min 39-42, Max 70-76 | 56 |
| 25^o^ C | Min 29-33, Max 52-65 | 44 |
| 28^o^ C | Min 22-24, Max 41-44 | 32 |
| 30^o^ C | Min 17-19, Max 33-54 | 30 |
| 31^o^ C | Min 17-19, Max 41-50 | 31 |
| 32^o^ C | Min 17-20, Max 35-48 | 30 |

*Oncomelania hupensis* with *S. japonicum* [32]*.*

| Temperature | Prepatent period (days) |
| --- | --- |
| 24^o^ C | - N/A |
| 18^o^ C | - |
| 21^o^ C | 125 |
| 24^o^ C | 90 |
| 27^o^ C | 71 |
| 30^o^ C | 62 |

The top five models *Biomphalaria* snails with with *S. mansoni*

| Model | AIC value | BIC value |
| --- | --- | --- |
| Irf | -354 | -345 |
| Thomas1 | -354 | -345 |
| Briere2 | -354 | -345 |
| Ratkowsky | -354 | -345 |
| Lactin2 | -354 | -345 |
| Gaussian (9th in ranking) | -353 | -346 |

Irf curve is selected and the fitted curve to *Biomphalaria* data as follow

| A  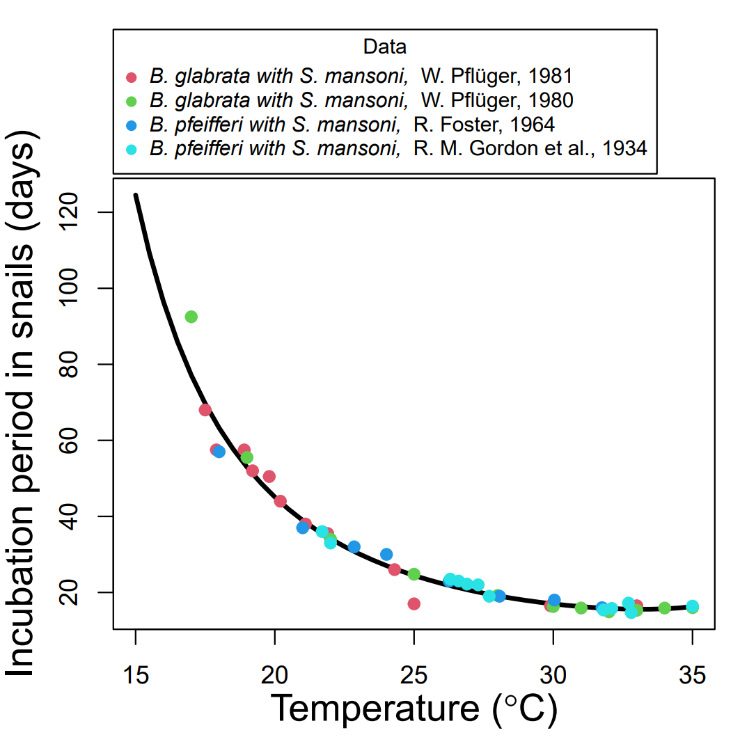 | B  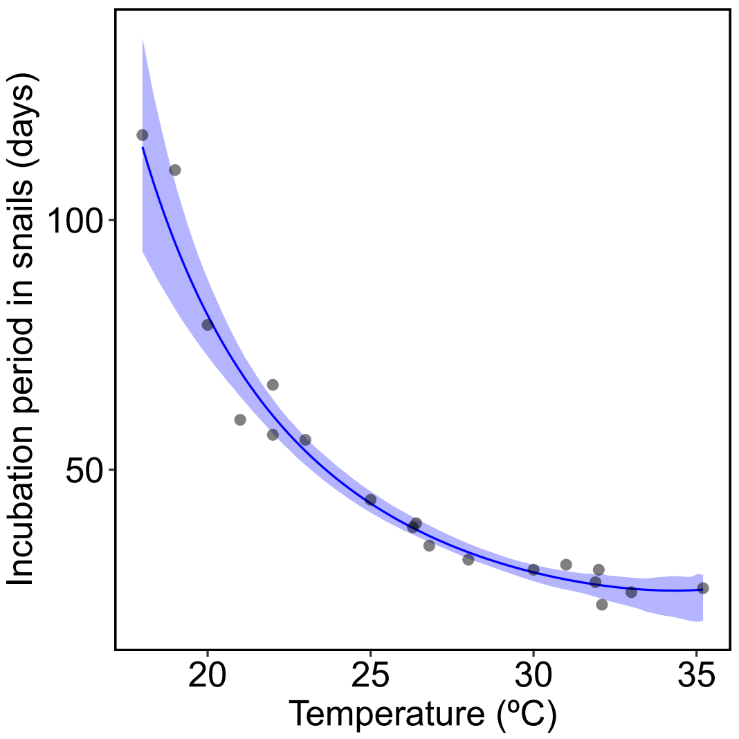 |
| --- | --- |

Fig E: Prepatent period of snails and confidence interval Biomphalaria Spp.

The top five models for *Bulinus* snails with

| Model | AIC value | BIC value |
| --- | --- | --- |
| Gaussian | -172 | -168 |
| Lactin2 | -171 | -166 |
| Briere2 | -171 | -166 |
| Thomas1 | -170 | -166 |
| lrf | -170 | -166 |

The Irf curve is selected and the fitted curve to *Bulinus* data as follow

| A  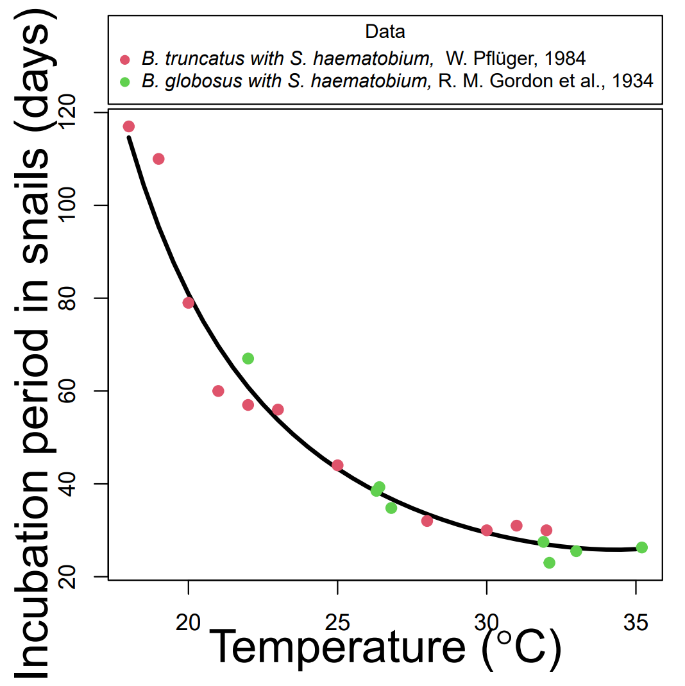 | B  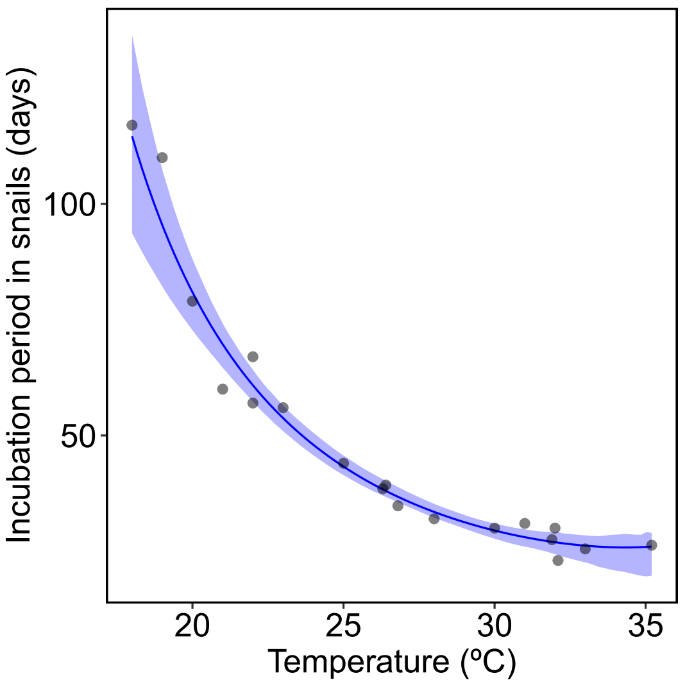 |
| --- | --- |

Fig F: Prepatent period of snails and confidence interval Bulinus Spp.

### The probability of hatching success of miracidia

$\delta_{e}$has the unit of parasite/egg and represents the rate of parasite hatch per egg. The only resource that we found is [33].

*S. mansoni* [33]*.*

| Temperature | Hatching rate |
| --- | --- |
| 5^o^ C | 0.2333333 |
| 9^o^ C | 0.2 |
| 13^o^ C | 0.3 |
| 17^o^ C | 0.33 |
| 21^o^ C | 0.253 |
| 22^o^ C | 0.38 |
| 25^o^ C | 0.456 |
| 29^o^ C | 0.35 |
| 33^o^ C | 0.5 |
| 37^o^ C | 0.45 |

*S. mansoni* [34]*.*

| Temperature | The half time (minutes) |
| --- | --- |
| 4^0^ | 120 |
| 12^0^ | 8 |
| 22^0^ | 4 |
| 34 | 2 |

The top five models

| Model | AIC value | BIC value |
| --- | --- | --- |
| Flinn | -23.3 | -22.1 |
| Quadratic | -23 | -21.8 |
| Gaussian | -22.5 | -21.3 |
| Modifiedgaussian | -21.9 | -20.4 |
| Spain | -21.8 | -20.3 |

Flinn curve is selected and has the following expression

[
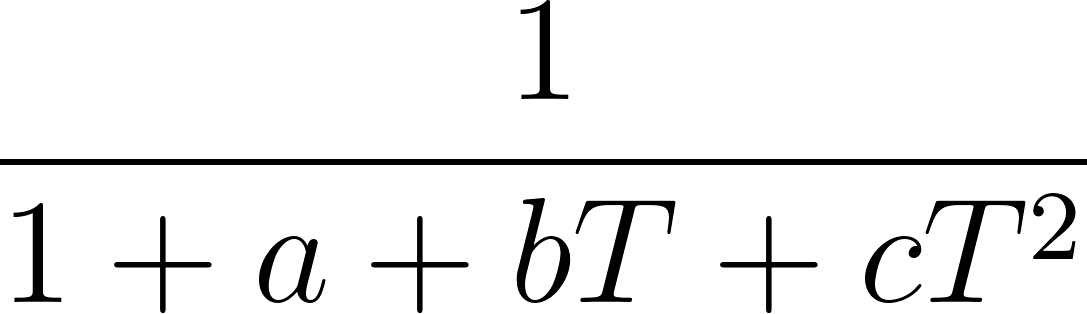
](https://www.codecogs.com/eqnedit.php?latex=%5Cfrac%7B1%7D%7B1%2Ba%2BbT%2BcT%5E2%7D#0)

See [35] for additional information. the fitted curve to *S. mansoni* data as follow

| A  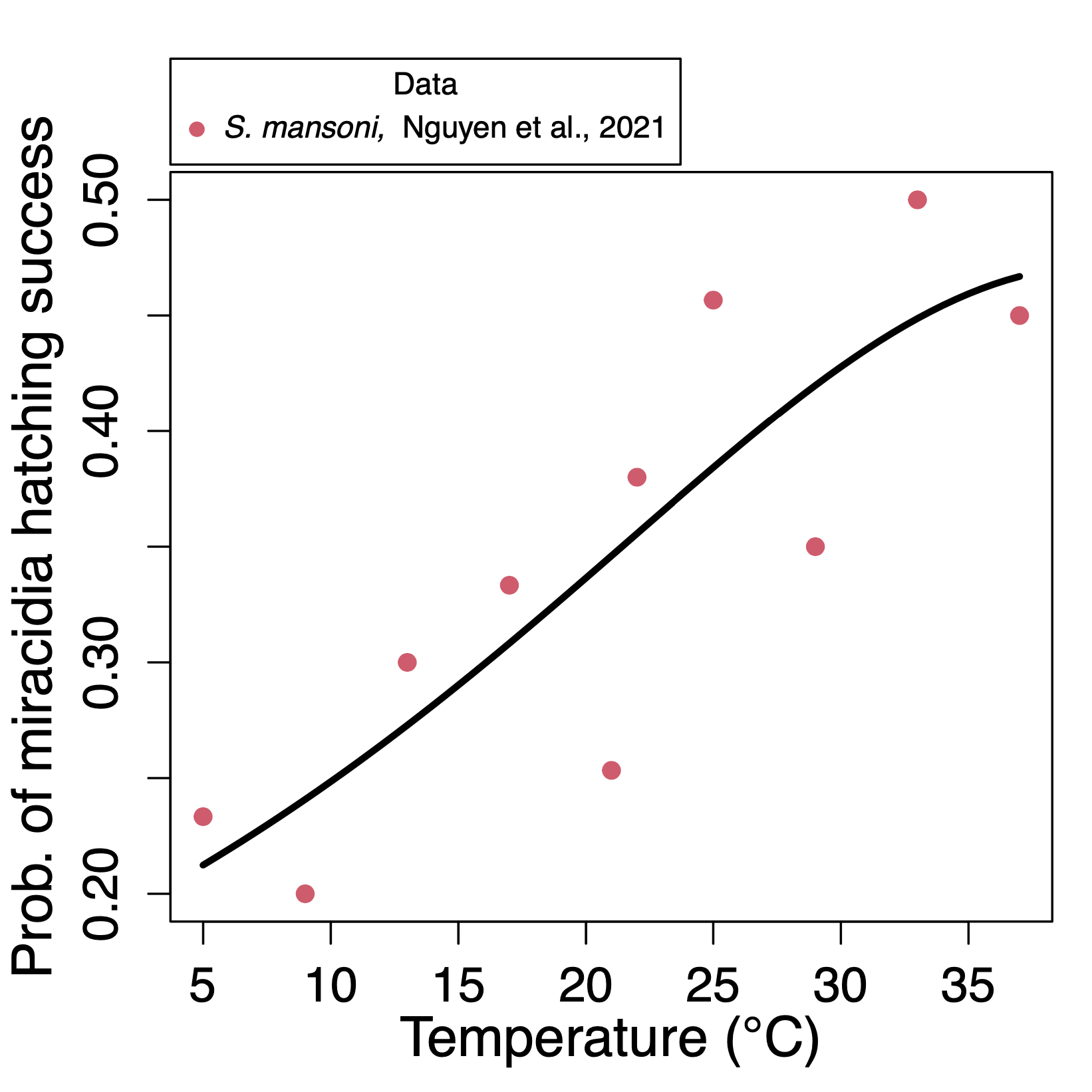 | B  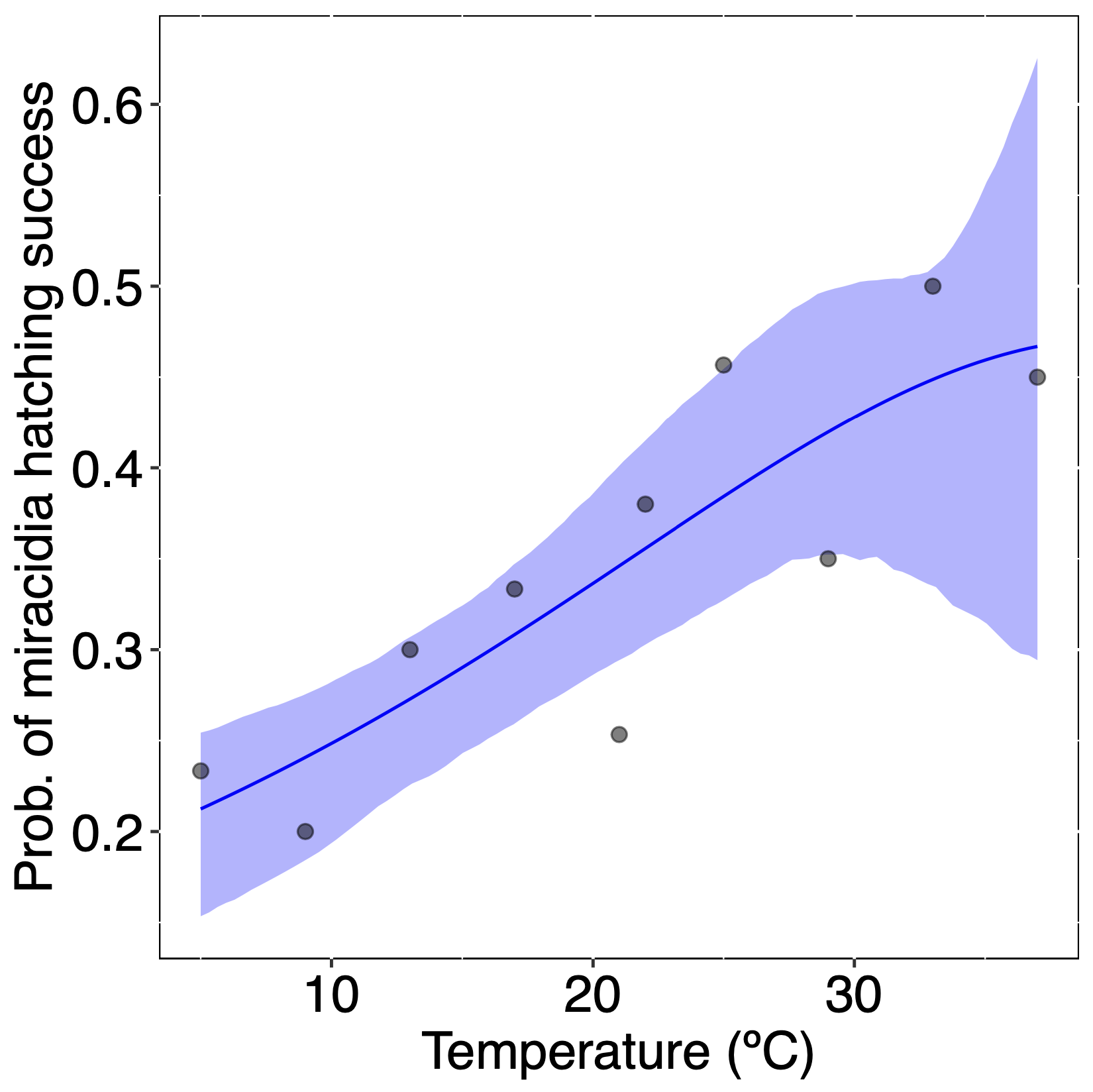 |
| --- | --- |

Fig G: Probability of hatching success of miracidia and confidence interval S. mansoni.

### The number of cercarial released per snail

$\nu_{c}$ has the unit of parasite/day*snail and represents the number of cercarial produced per snail per day.

*Biomphalaria glabrata* with *S. mansoni* [33]*.*

| Temperature | The number of cercarial releases per day per snail |
| --- | --- |
| 5^o^ C | 0 |
| 9^o^ C | 15 |
| 13^o^ C | 260 |
| 17^o^ C | 1350 |
| 21^o^ C | 1970 |
| 25^o^ C | 2900 |
| 29^o^ C | 1970 |
| 33^o^ C | 1770 |
| 37^o^ C | 690 |

[36] presented data on the number of cercariae released by *Biomphalaria glabrata* during one hour under various conditions. Based on their standard (incandescent light) conditions, at different temperatures, the daily cercaria production rates (per infected snail) are:

*Biomphalaria glabrata* with *S. mansoni*

| Temperature | The number of cercarial release per hour per snail | The number of cercarial release per day per snail, adjusted based on Nguyen’s results |
| --- | --- | --- |
| 12^o^ C | 21 | 352 day^-1^ per 24 hours |
| 35^o^ C | 350 | 5880 day^-1^ per 24 hours |

*Australorbis glabratus* with (*B. glabrata) S. mansoni* [37]*.* (records are daily but from 10AM to 2PM)

| Temperature | The number of cercarial release per day per snail |
| --- | --- |
| 23 to 25^o^ C | 784 |
| 26 to 28^o^ C | 838 |

*Australorbis glabratus (B. glabrata)* with *S. mansoni* [38].

Temperature is 30^o^ and the average number of cercarial produced from 9 am to 11 am is 511 when it is dark, and 692 when it is light. From 11 am to 3 pm is 340 when it is dark, 654 when it is light.

*B. glabrata* with *S. mansoni* [39]*.*

| Temperature | Th number of cercaria after crushing the snail |
| --- | --- |
| 10^0^ | 0 |
| 13^0^ | 0 |
| 16^0^ | 206 |
| 19^0^ | 114 |
| 22^0^ | 442 |
| 25^0^ | 1200 |
| 28^0^ | 1400 |
| 31^0^ | 1233 |
| 34^0^ | 1241 |
| 37^0^ | 243 |
| 40^0^ | 121 |

*Bulinus globosus* with *S. haematobium* [18]*.*

| Temperature | The number of cercarial release per day per snail |
| --- | --- |
| 21.2^o^ C | 1738 |
| 25.8^o^ C | 2409 |
| 31^o^ C | 829 |

The top five models for *Biomphalaria* snails

| Model | AIC value | BIC value |
| --- | --- | --- |
| Gaussian | 314 | 318 |
| Flinn | 315 | 319 |
| Ratkowsky | 316 | 321 |
| Weibull | 316 | 321 |
| Oneill | 316 | 321 |

The Gaussian curve is selected and the fitted curve to *Biomphalaria* data as follow

| A  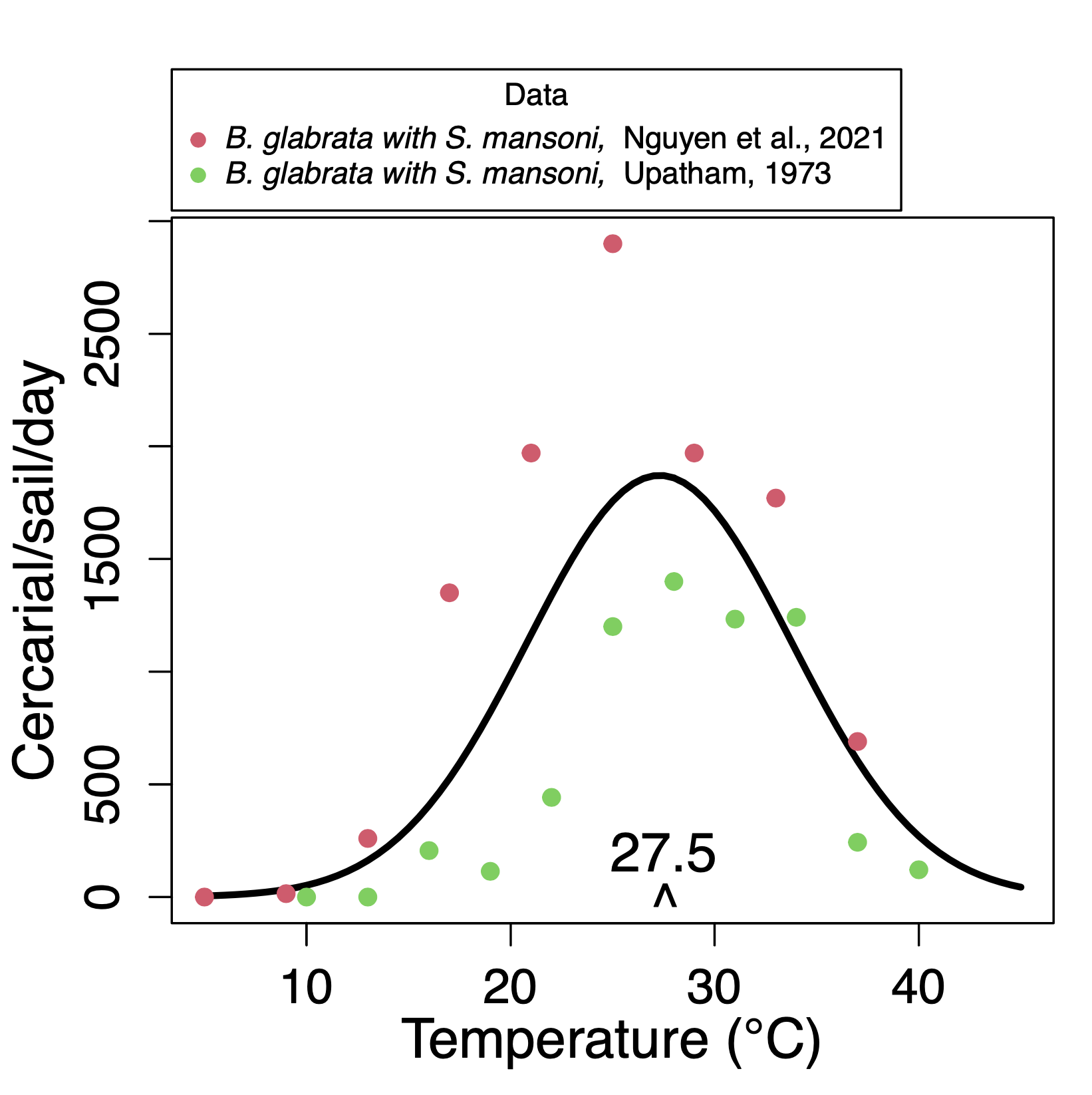 | B  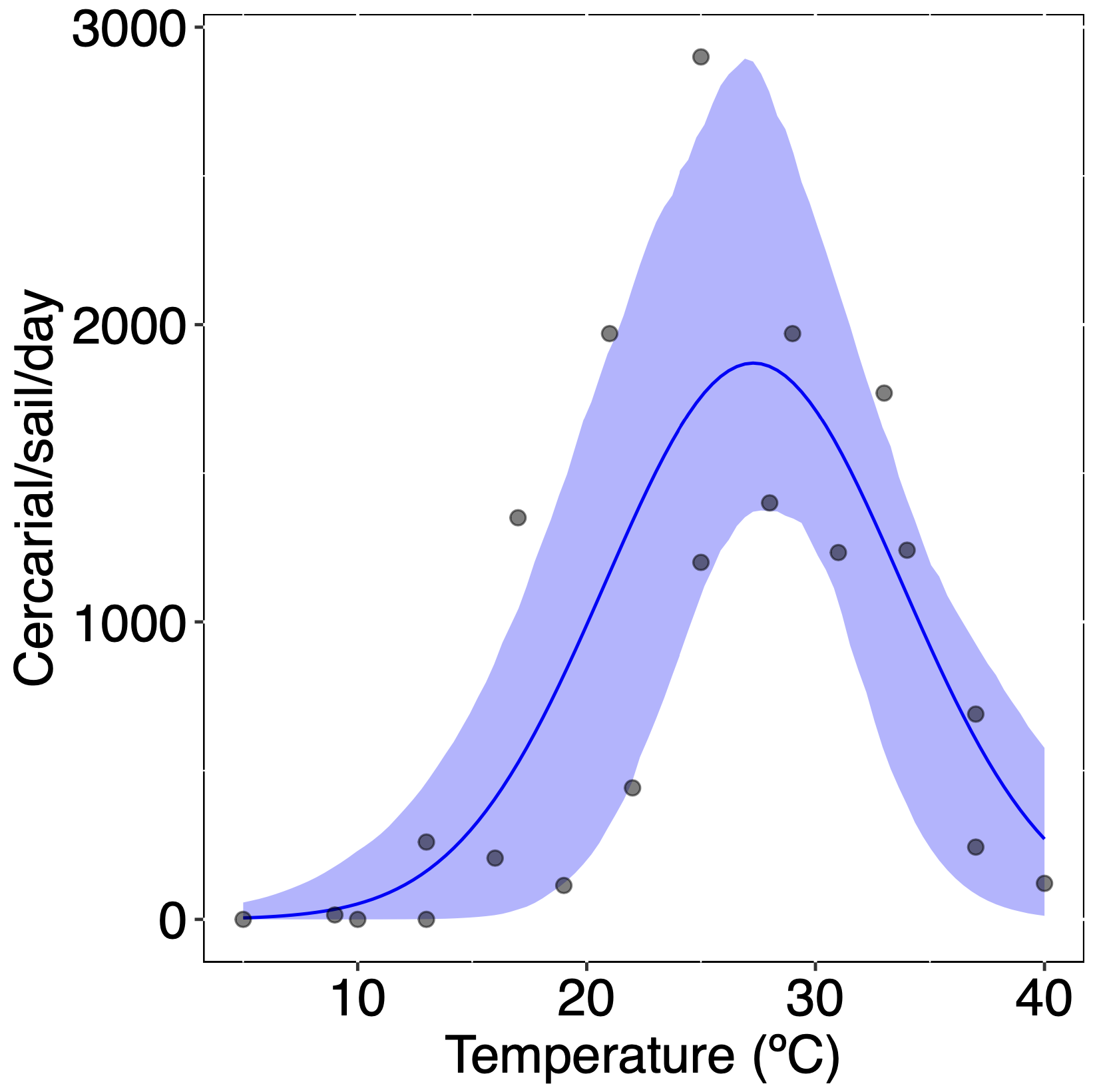 |
| --- | --- |

Fig H: Cercariae release rate and confidence interval Biomphalaria Spp.

### The mortality rate of miracidia

$\mu_{m}$ has the unit of 1/day and represents the mortality rate of miracidia per day.

[40] gives the life expectancy in hour for *S. mansoni*. We convert to the day unit.

| Temperature | The mortality rate of miracidia (1/day) |
| --- | --- |
| 5^o^ C | 4.957653 |
| 10^o^ C | 2.141901 |
| 15^o^ C | 1.50839 |
| 20^o^ C | 1.974984 |
| 25^o^ C | 2.513615 |
| 30^o^ C | 4.322767 |
| 35^o^ C | 4.490178 |
| 40^o^ C | 5.114 |

[41] experiment with *S. mansoni* and two-time range

| Temperature | 2 hours at temperature | 6 hours at temperature | The mortality rate of miracidia |
| --- | --- | --- | --- |
| 12^o^ C | 3.261705 | 1.637893 | 2.449799 |
| 14^o^ C | 2.396054 | 1.785148 | 2.090601 |
| 16^o^ C | 2.904859 | 1.957561 | 2.43121 |
| 18.5^o^ C | 1.520372 | 3.054279 | 2.287326 |
| 21.5^o^ C | 2.337589 | 2.812790 | 2.57519 |
| 24.8^o^ C | 2.752958 | 3.891444 | 3.322201 |
| 28.6^o^ C | 3.842463 | 8.757026 | 6.299745 |
| 32.7^o^ C | 4.877587 | 9.721674 | 7.299631 |

Two studies [42] [41] give the percentage of miracidia survival for different times at each temperature. By using these percentages and the method described in the mortality rate of snails, we found the mortality rate of miracidia as follow

*S. mansoni* [42].

| Temperature | The mortality rate of miracidia |
| --- | --- |
| 5-10^o^ C | 2.56078 |
| 18-22^o^ C | 0.6536434 |
| 25-30^o^ C | 1.499468 |
| 35-38^o^ C | 3.940166 |

*S. haematobium* [42].

| Temperature | The mortality rate of miracidia |
| --- | --- |
| 5-10^o^ C | 2.275331 |
| 18-22^o^ C | 2.210028 |
| 25-30^o^ C | 2.24239 |
| 35-38^o^ C | 4.354702 |

The top five models for *S. mansoni*

| Model | AIC value | BIC value |
| --- | --- | --- |
| Thomas2 | 73.6 | 79.6 |
| Modifiedgaussian | 74 | 78.9 |
| Spain | 74.1 | 79 |
| Quadratic | 74.2 | 78.2 |
| lrf | 75.3 | 80.3 |

Thomas2 curve is selected and has the following expression

$$ae^{\mathrm{bT}}-\left( c+de^{\mathrm{eT}} \right)$$

see [43] for more information and the fitted curve to *S. mansoni* data as follow

| A  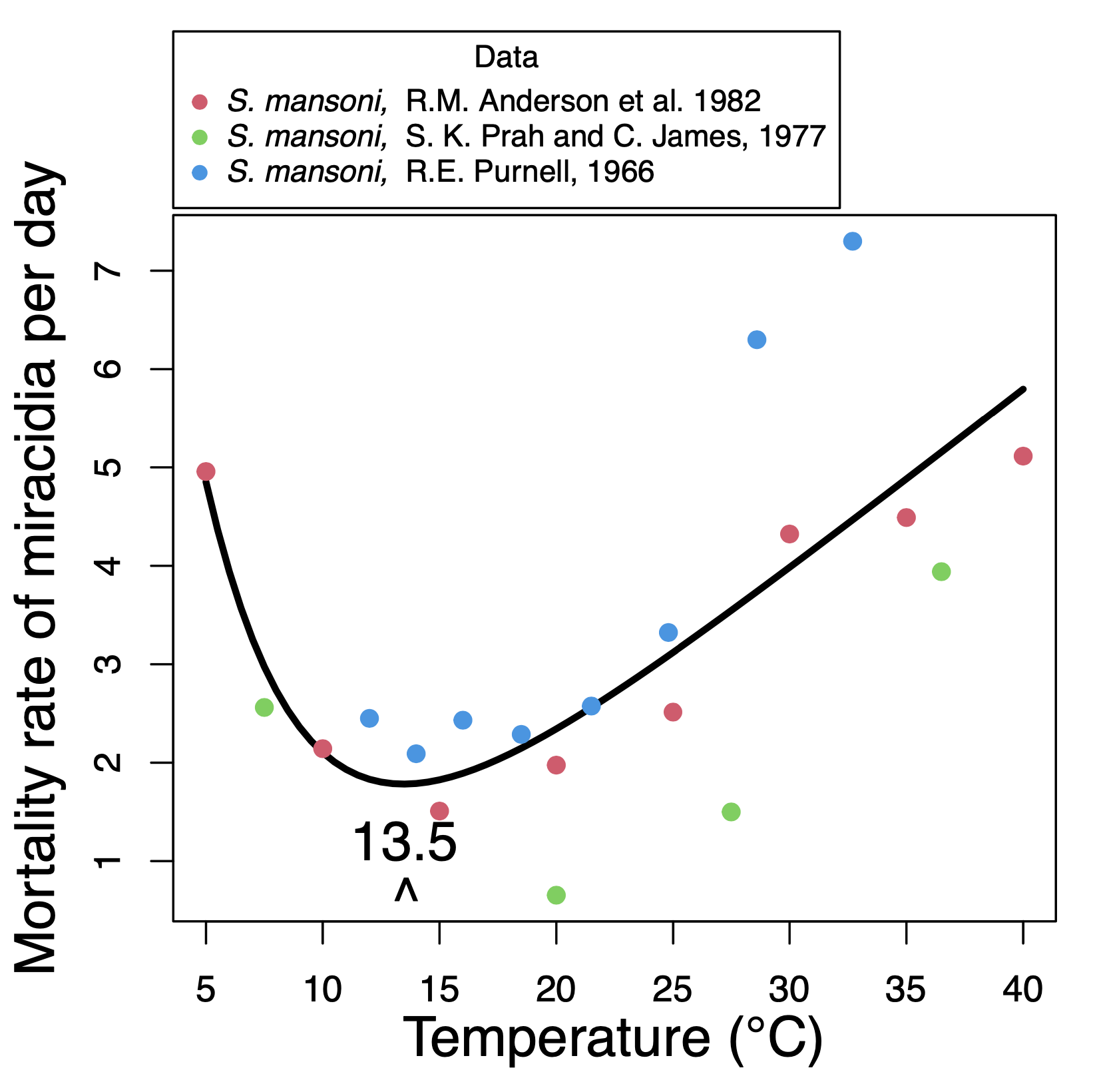 | B  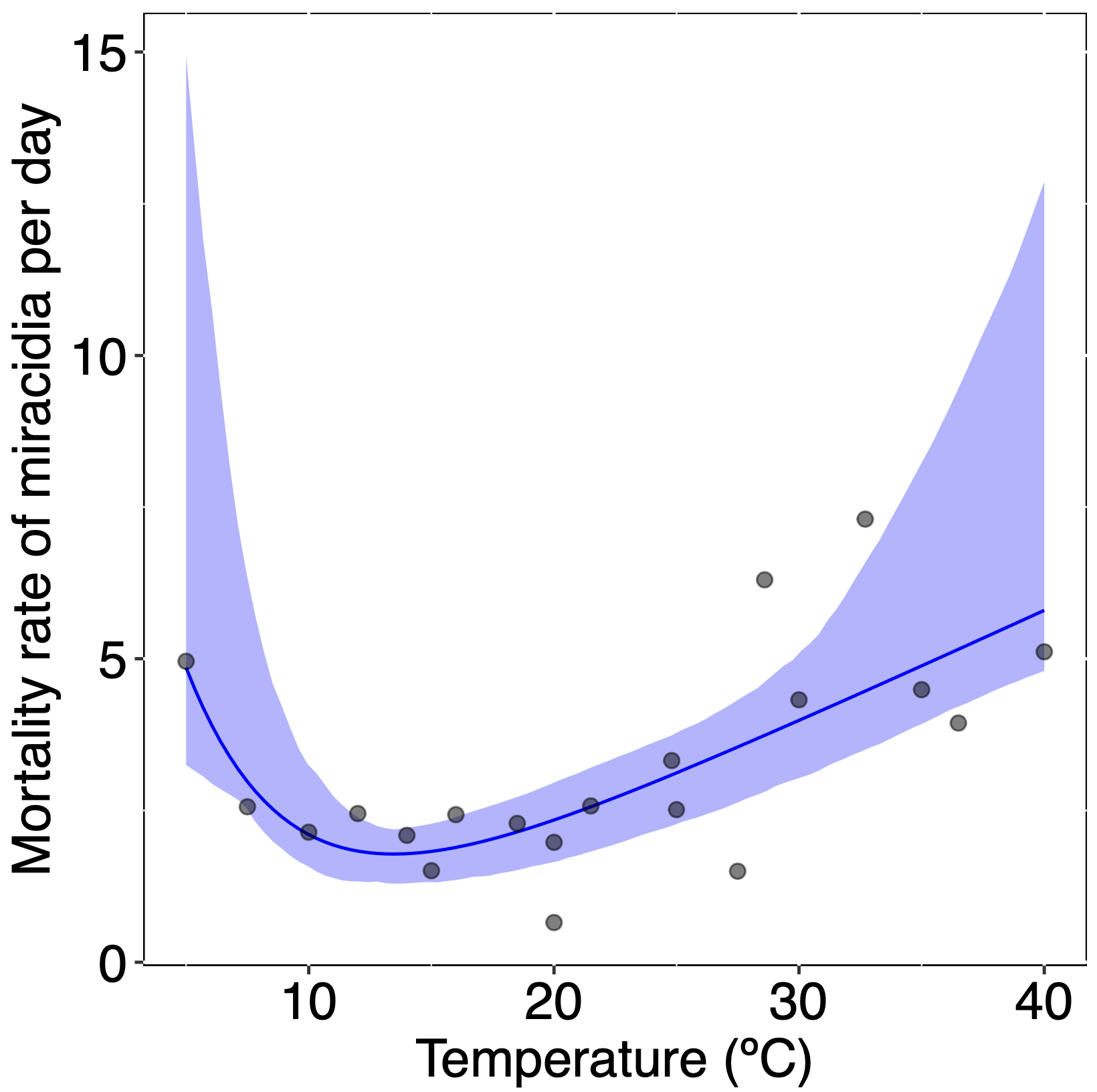 |
| --- | --- |

Fig I: Mortality rate of miracidia and confidence interval S. mansoni.

The top five models for *S. haematobium*

| Model | AIC value | BIC value |
| --- | --- | --- |
| Joehnk | -0.509 | -0.833 |
| Spain | 0.563 | 0.292 |
| Thomas2 | 2.00 | 1.67 |
| Quadratic | 5.57 | 5.35 |
| Modifiedgaussian | 8.62 | 8.35 |

Spain curve is selected and the fitted curve to *S. haematobium* data as follow

| A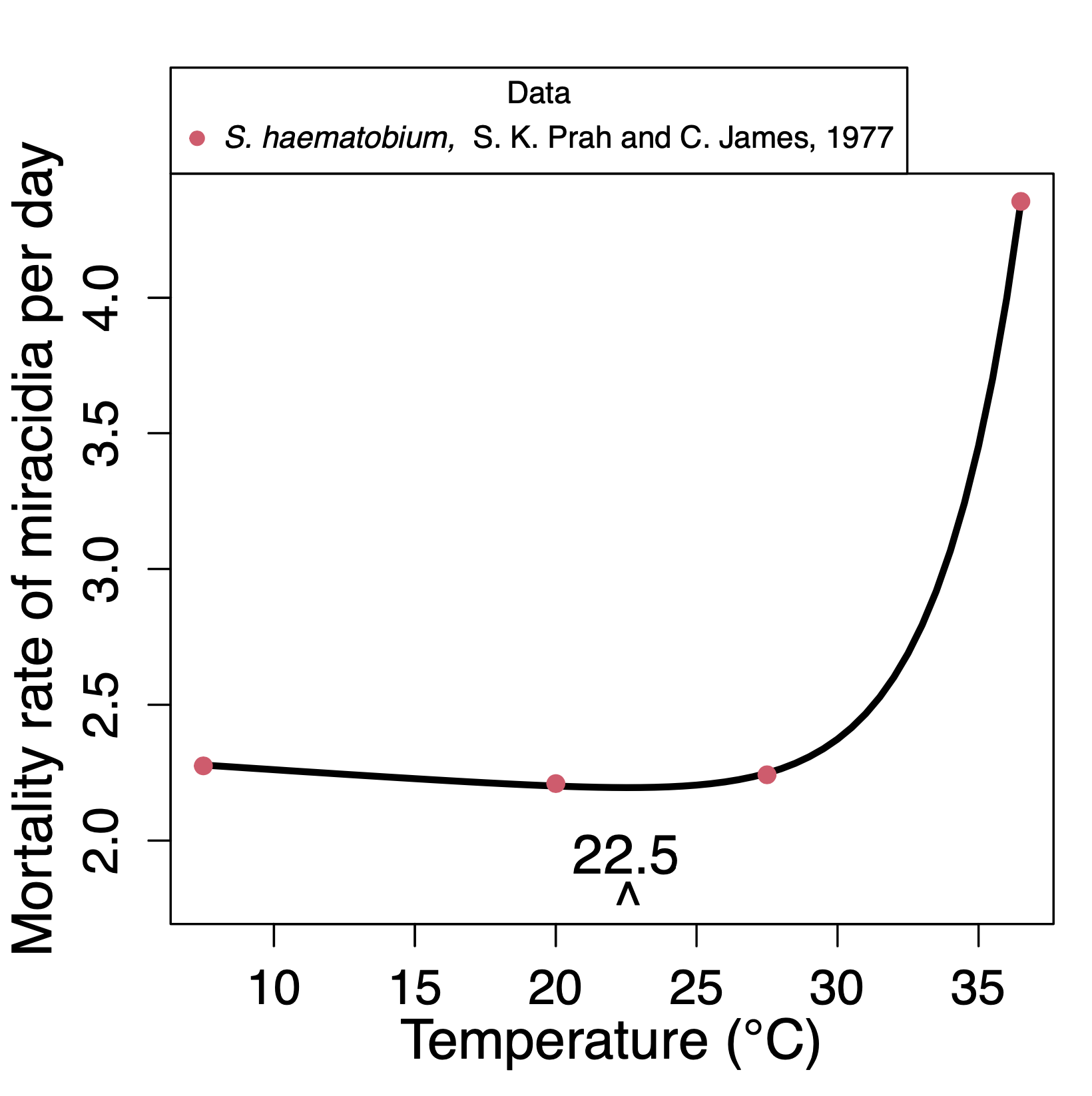 | B  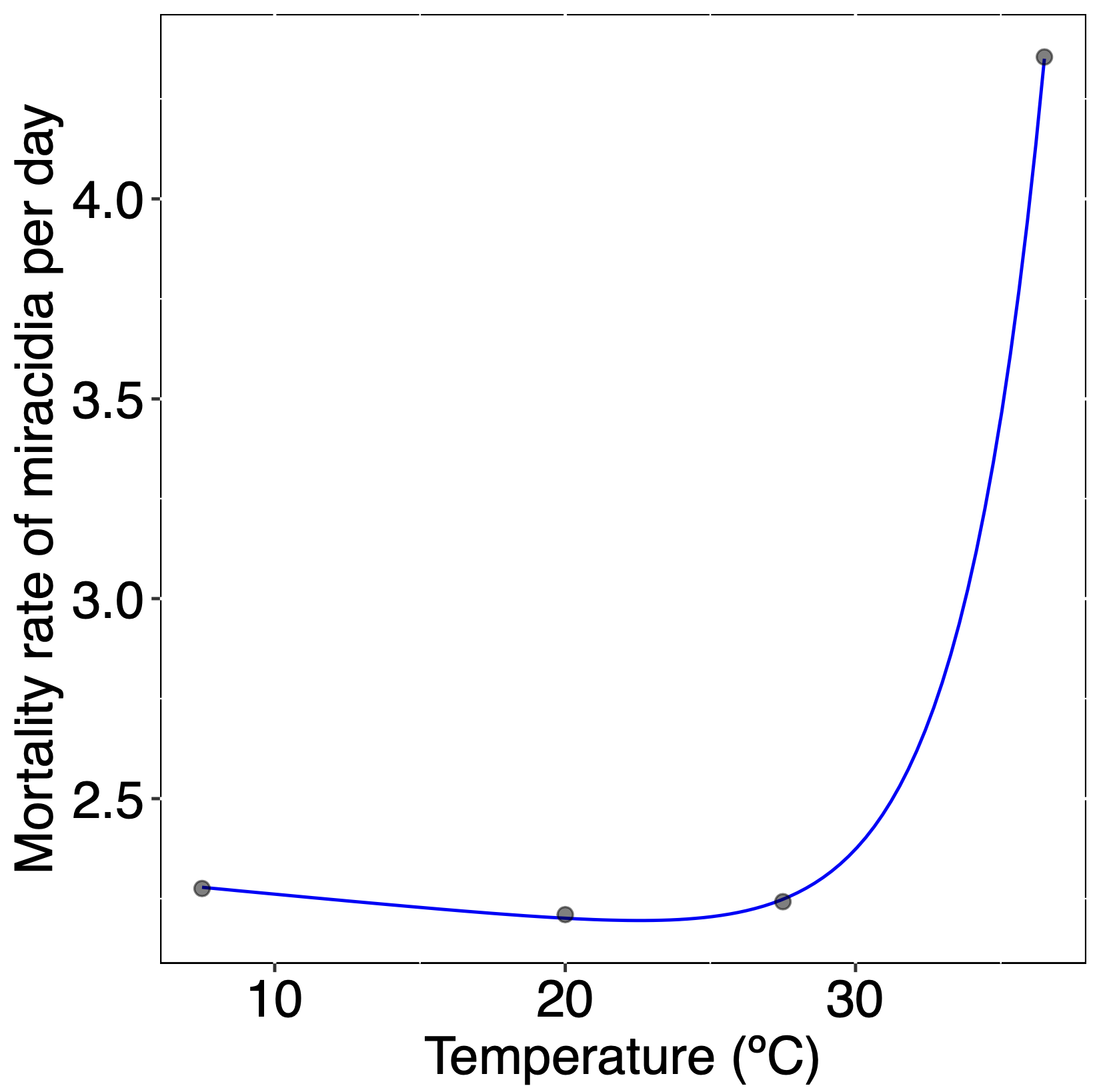 |
| --- | --- |

Fig J: Mortality rate of miracidia and confidence interval S. haematobium.

The mortality rate of cercaria

$\mu_{c}$ has the unit 1/day and represents the death rate of cercaria per day.

Two studies [44] and [41] give the percentage of cercaria survival at different times at each temperature. By using these percentages and the method described in the mortality rate of snails, we found the mortality rate of cercaria as follows

*S. mansoni* [44]*.*

| Temperature | The mortality rate of cercarial |
| --- | --- |
| 10^o^ C | 0.6844903 |
| 15^o^ C | 0.347094 |
| 20^o^ C | 0.9361688 |
| 25^o^ C | 0.9384906 |
| 30^o^ C | 1.767248 |
| 35^o^ C | 3.79802 |
| 40^o^ C | 7.210983 |

*S. mansoni* [41]*.*

| Temperature | 2 hours | 6 hours | 24 hours | The mortality rate of cercarial |
| --- | --- | --- | --- | --- |
| 12^o^ C | 1.520372 | 0.9277282 | 0.5395681 | 0.9958894 |
| 15^o^ C | 3.468196 | 2.036641 | 0.9467499 | 2.150529 |
| 18^o^ C | 2.677723 | 1.735458 | 0.7765288 | 1.729903 |
| 21^o^ C | 1.171354 | 1.358709 | 0.549913 | 1.026659 |
| 24^o^ C | 1.264326 | 1.199019 | 0.7918632 | 1.085069 |
| 27^o^ C | 1.992655 | 0.9327755 | 0.7657179 | 1.230383 |
| 30^o^ C | 1.893889 | 1.369961 | 1.890475 | 1.718108 |
| 33^o^ C | 1.291023 | 3.745974 | NA | NA |

*S. mansoni* [45]*.* ​​(He analyze only death rate of cercaria during the penetration. We do not use in our simulaiton)

| Temperature | Mortality rate |
| --- | --- |
| 10^0^ | 9.66236 |
| 40^0^ | 10.58267 |
| 25-27^0^ | 4.435385 |

*S. haematobium* [45]*.*

| Temperature | Mortality rate |
| --- | --- |
| 10^0^ | 4.280099 |
| 40^0^ | 5.169395 |
| 25-27^0^ | 1.950227 |

The top five models for *S. mansoni*

| Model | AIC value | BIC value |
| --- | --- | --- |
| Rezende | 24.5 | 27.7 |
| Spain | 26.8 | 30 |
| Joehnk | 26.9 | 30.7 |
| Flinn | 28.2 | 30.7 |
| Thomas2 | 28.3 | 30.2 |

Spain curve is selected and the fitted curve to *S. mansoni* data as follow

| A  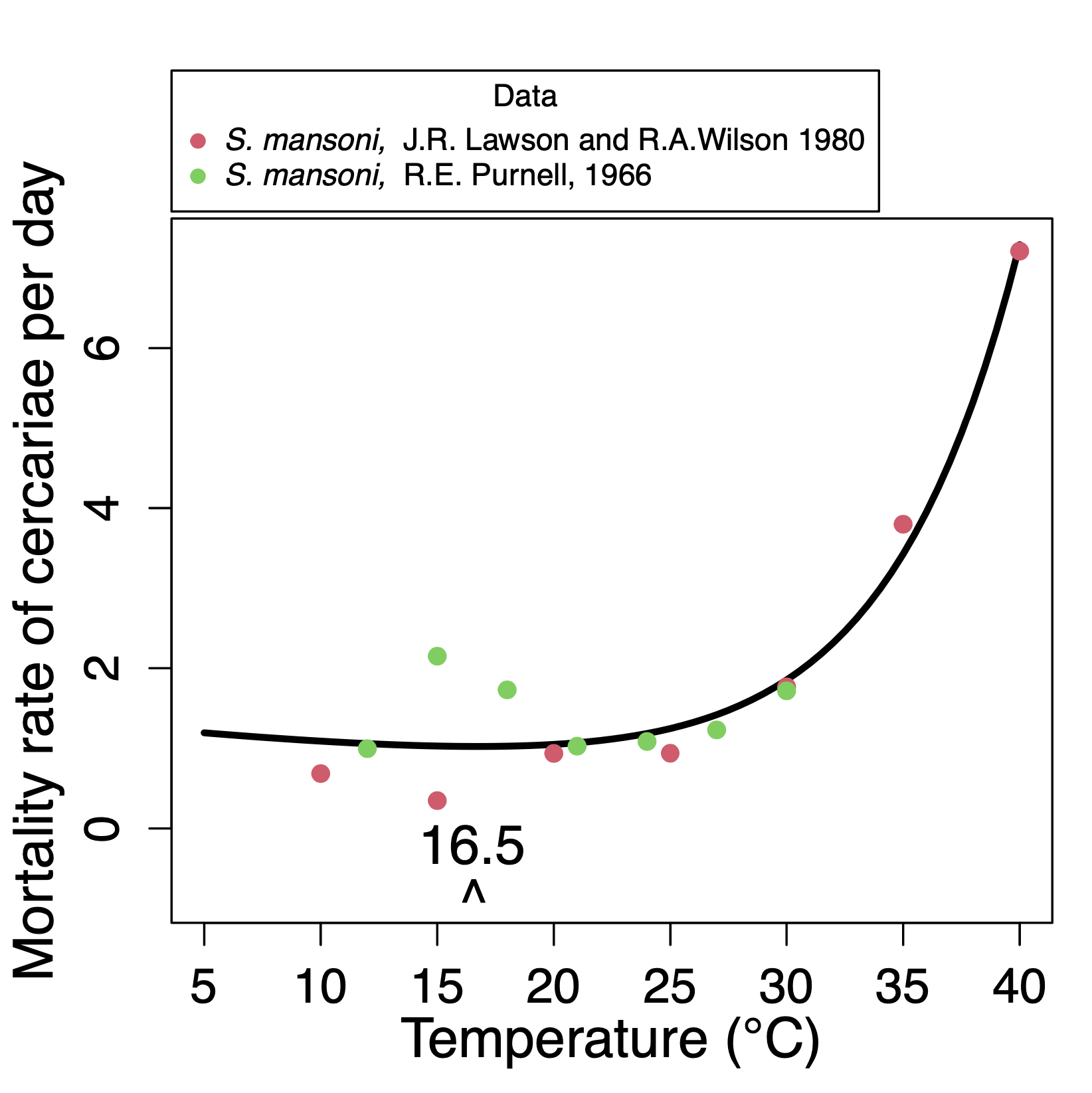 | B  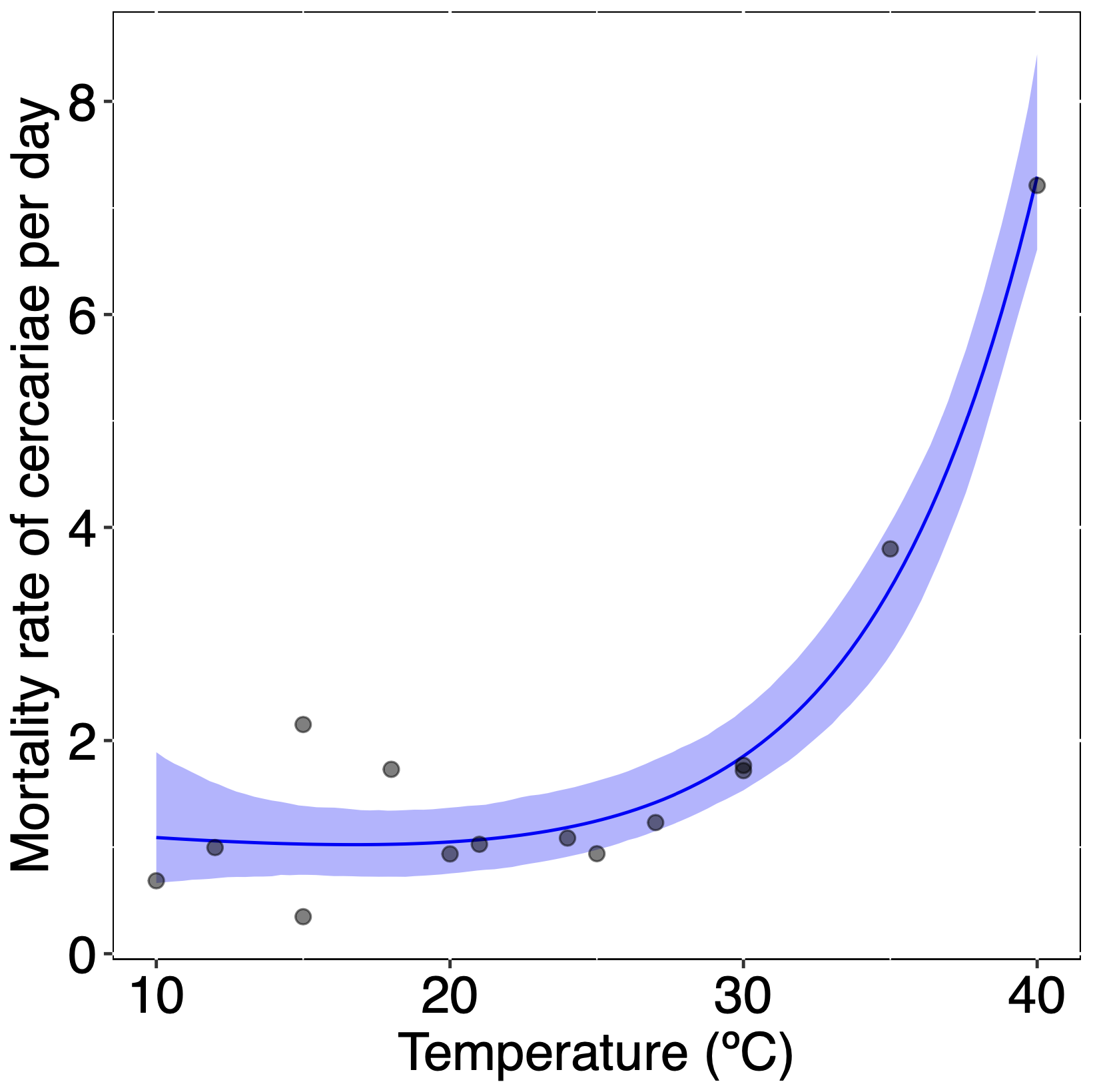 |
| --- | --- |

Fig K: Mortality rate of cercaria and confidence interval S. mansoni.

Since we do not have data for *S. haematobium*. We do not have a curve.

### The mortality rate of infected snails

$\mu_{i}$ has the unit of 1/day. The value represents the death rate of snails due to infection per day.

The data is given as the percentage of death in snails after exposed to miracidia for each day. So we use the method that we use in the mortality rate of snails to find the mortality rate of infected snails.

*Biomphalaria glabrata* and *S. mansoni,* [28]. This result from prepatent snails. This study has two experiments; the first is diurnally changing water temperature, and the second one is a sinusoidal variation of diurnal temperature.

First experiment

| Temperature | Prepatent period | Average percent of mortality | Death rate |
| --- | --- | --- | --- |
| 24.3^o^C | (25+27)/2 = 26 (days) | (46+49)/2 = 47.5 | 0.02478296 |
| 21.9^o^C | 36 (days) | (43+65)/2 = 54 | 0.02157024 |
| 20.2^o^C | 44 (days) | (75+68)/2 = 71.5 | 0.02852877 |
| 19.7^o^C | 48 (days) | (66+73)/2 = 69.5 | 0.02473841 |

Second experiment

| Temperature | Prepatent period | Average percent of mortality | Death rate |
| --- | --- | --- | --- |
| 17.5^o^C | 68 (days) | 30 | 0.00524522 |
| 17.9^o^C | 57 (days) | 65 | 0.01841793 |
| 19.2^o^C | 52 (days) | 40 | 0.00982357 |
| 21.1^o^C | 38 (days) | 27 | 0.008281862 |
| 18.9^o^C | 57 (days) | (75+77)/2 = 76 | 0.02503713 |
| 25^o^C | 24 (days) | 28 | 0.01368767 |
| 29.9^o^C | 17 (days) | 30 | 0.02098088 |
| 33.1^o^C | 17 (days) | (18+79)/2 = 48.5 | 0.03903461 |

*Biomphalaria glabrata* and *S. mansoni*  [29]. This result from prepatent snails.

| Temperature | Prepatent perioed | Average percent of mortality | Death rate |
| --- | --- | --- | --- |
| 16^o^C | 140 (days) | (95+96+98)/3 = 96.3 | 0.02354884 |
| 17^o^C | 92.5 (days) | (90+58)/2 = 74 | 0.01456296 |
| 18^o^C | 70 (days) | (85+65)/2 = 75 | 0.01980421 |
| 19^o^C | 55.5 (days) | (57+38)/2 = 47.5 | 0.01161004 |
| 22^o^C | 34 (days) | (51+64+38)/3 = 51 | 0.02098088 |
| 25^o^C | 24.8 (days) | (42+58+48+22+33+20)/6 = 37 | 0.01863046 |
| 28^o^C | 19.2 (days) | (9+17+33)/3 = 19.6 | 0.01136229 |
| 30^o^C | 16.3 (days) | 19 | 0.01292767 |
| 31^o^C | 15.9 (days) | (12+13+27)/3 = 17.3 | 0.01194658 |
| 32^o^C | 14.9 (days) | (29+7+3)/3 = 13 | 0.009346447 |
| 33^o^C | 15.3 (days) | (43+8)/2 = 25.5 | 0.01923994 |
| 34^o^C | 15.9 (days) | (62+79)/2 = 70.5 | 0.07677861 |
| 35^o^C | 16 (days) | (47+50)/2 = 48.5 | 0.04147427 |

*Biomphalaria pfeifferi* and *S. mansoni* [24]*.*

| Temperature | Death rate |
| --- | --- |
| 22.85^o^C | 0.006674626 |
| 24.01^o^C | 0.01155245 |
| 26.26^o^C | 0.0260108 |
| 28.07^o^C | 0.03678792 |

*B. glabrata* with *S. mansoni* [39].

| Temperature | Percent of survival after 12 days | Death rate |
| --- | --- | --- |
| 10^0^ | 92.7 | 0.0058551278 |
| 13^0^ | 89.3 | 0.0085350331 |
| 16^0^ | 93 | 0.0061192732 |
| 19^0^ | 78.2 | 0.0195844770 |
| 22^0^ | 89.7 | 0.0085350331 |
| 25^0^ | 95.35 | 0.0032587267 |
| 28^0^ | 99.5 | 0.0004931228 |
| 31^0^ | 92.8 | 0.0061192732 |
| 34^0^ | 92.8 | 0.0058551278 |
| 37^0^ | 30.2 | 0.0968812480 |
| 40^0^ | 33.3 | 0.0915518574 |

*Bulinus globosus* and *S. haematobium* [18]. This result from prepatent snails.

| Temperature | Death rate |
| --- | --- |
| 15.5°C | 0.00244925 |
| 21.2°C | 0.006901087 |
| 25.8°C | 0.01151302 |
| 31°C | 0.02073716 |
| 36°C | 0.04642702 |

*Bulinus truncatus* and *S. haematobium* [31]. This result from prepatent snails.

| Temperature | Length of prepatent | Average | Percentage of death | Death rate |
| --- | --- | --- | --- | --- |
| 17^o^C | No infection | - | - | - |
| 33^o^C | No infection | - | - | - |
| 18^o^C | Min 106-113, Max 120-129 (days) | [109.5, 124.5] = 117 | 78.6 | 0.0131776 |
| 19^o^C | Min 75-78, Max 137-151 (days) | [76.5, 144] = 110.25 | 71.6 | 0.01141752 |
| 20^o^C | Min 64-67, Max 91-95 (days) | [65.5, 93] = 79.25 | 78.9 | 0.01963277 |
| 21^o^C | Min 41-56, Max 69-75 (days) | [48.5, 72] = 60.25 | 72.3 | 0.02130685 |
| 22^o^C | Min 44-54, Max 62-69 (days) | [49, 65.5] = 57.25 | 71.7 | 0.02204905 |
| 23^o^C | Min 39-42, Max 70-76 (days) | [40.5, 73] = 56.75 | 77.6 | 0.02636316 |
| 25^o^C | Min 29-33, Max 52-65 (days) | [31, 58.5] = 44.75 | 62.4 | 0.02185846 |
| 28^o^C | Min 22-24, Max 41-44 (days) | [23, 42.5] = 32.75 | 40.6 | 0.01590461 |
| 30^o^C | Min 17-19, Max 33-54 (days) | [18, 43.5] = 30.75 | 38.3 | 0.01570362 |
| 31^o^C | Min 17-19, Max 41-50 (days) | [18, 45.5] = 31.75 | 54.4 | 0.02473268 |
| 32^o^C | Min 17-20, Max 35-48 (days) | [18.5, 41.5] = 30 | 54.5 | 0.0262486 |

*Bulinus truncatus* with *S. haematobium* [46]*.*

| Temperature | Number of exposed snails | Number of snails alive on the 50^th^ day after exposure | Death rate |
| --- | --- | --- | --- |
| 10^0^ | 100 | 85 | 0.003250379 |
| 12^0^ | 50 | 40 | 0.004462871 |
| 14^0^ | 50 | 42 | 0.003487068 |
| 15^0^ | 100 | 90 | 0.00210721 |
| 20^0^ | 100 | 80 | 0.004462871 |
| 25^0^ | 150 | 118 | 0.004799013 |
| 30^0^ | 97 | 87 | 0.002176057 |
| 35^0^ | 97 | 89 | 0.001721492 |
| 38^0^ | 45 | 37 | 0.003914892 |

The top five models for *Biomphalaria* *snails*

| Model | AIC value | BIC value |
| --- | --- | --- |
| Joehnk (1st) | -223 | -213 |
| Spain (2nd) | -219 | -211 |
| Thomas2 (3rd) | -218 | -208 |
| Irf (4th) | -216 | -208 |
| Modifiedgaussian (5th) | -213 | -205 |

Flinn curve is selected and the fitted curve to *Biomphalaria* data as follow

| A  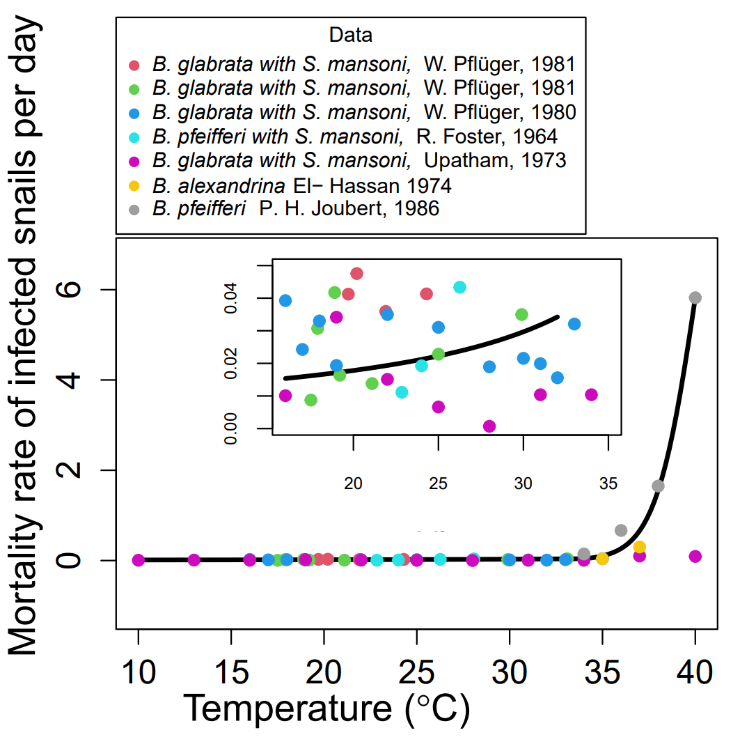 | B  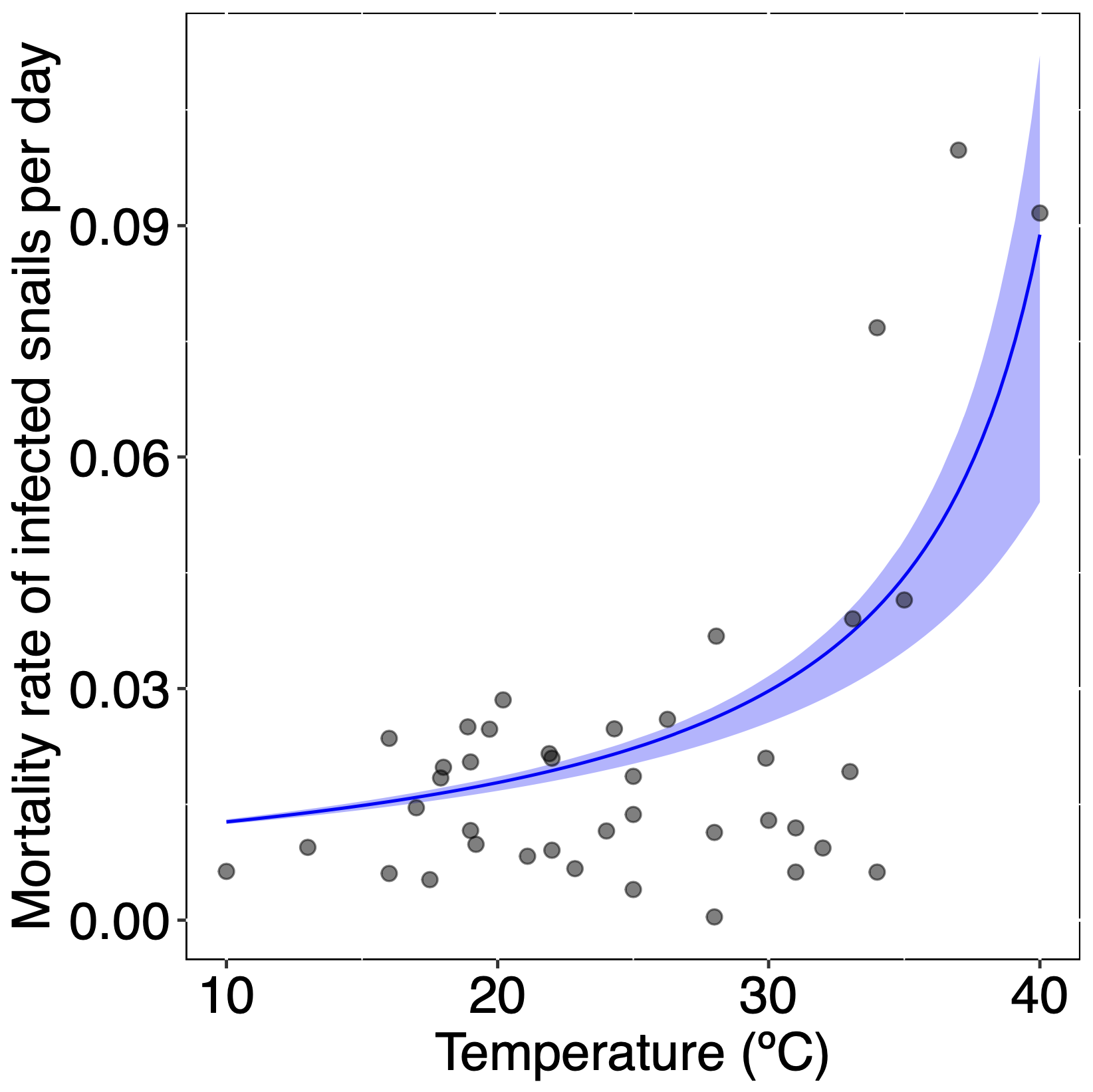 |
| --- | --- |

Fig L: Mortality infected snails and confidence interval Biomphalaria Spp.

The top five models for *Bulinus* snails.

| Model | AIC value | BIC value |
| --- | --- | --- |
| Joehnk | -173 | -165 |
| Weibull | -160 | -154 |
| Spain | -152 | -146 |
| Thomas | -151 | -143 |
| Irf | -149 | -143 |

Spain curve is selected and the fitted curve to *Bulinus* data as follow

| A  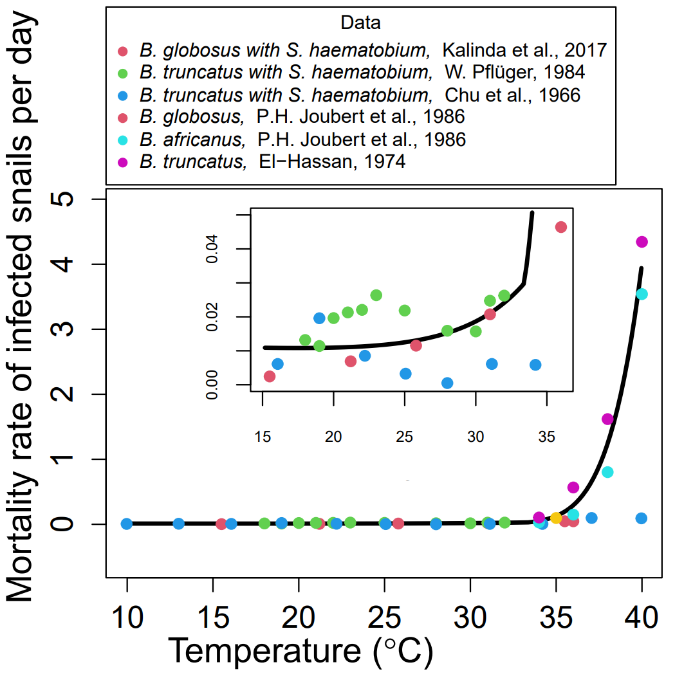 | B  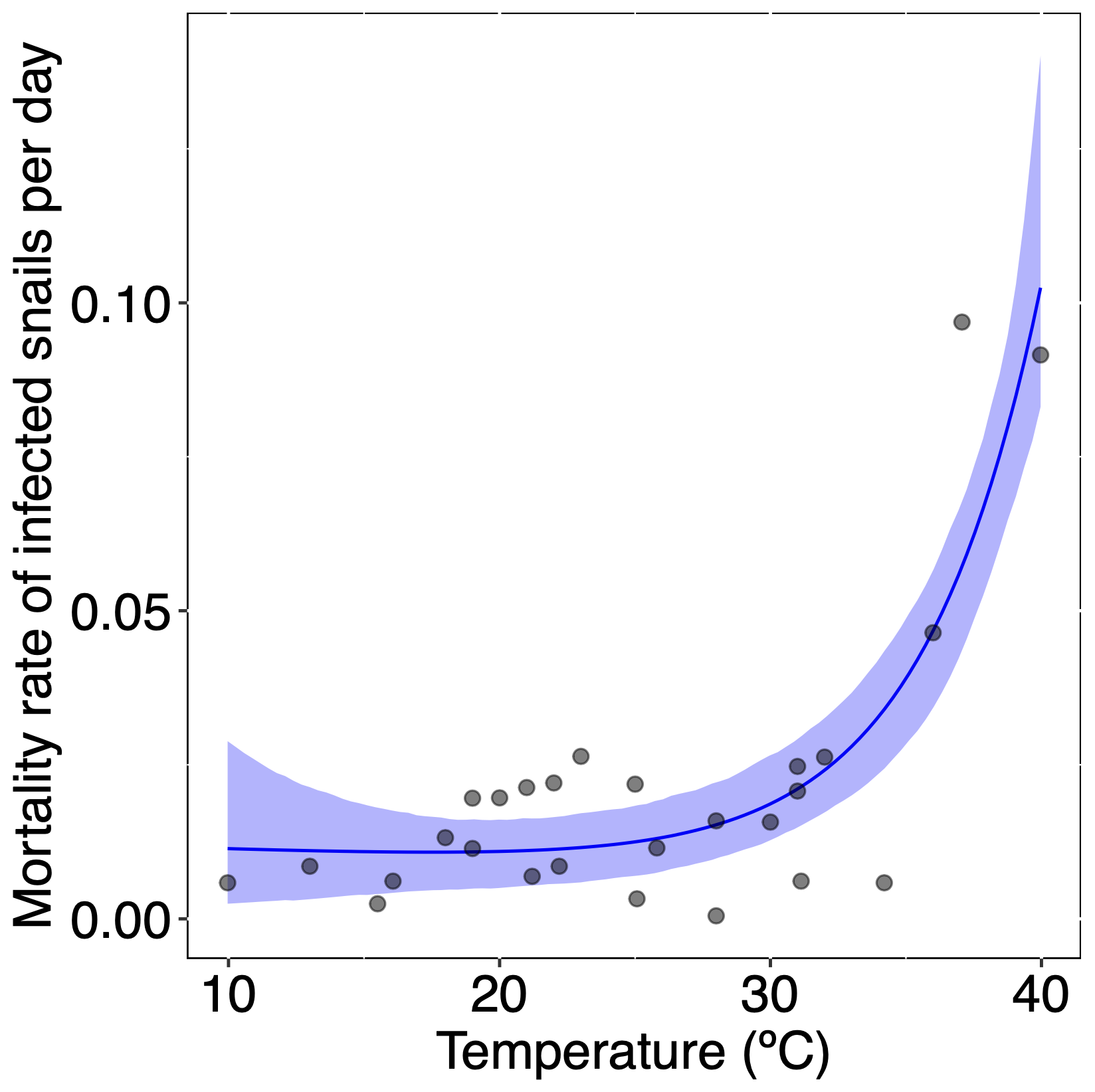 |
| --- | --- |

Fig M: Mortality infected snails and confidence interval Bulinus Spp.

### The transmission rate of schistosomiasis in snails

$\beta_{s}$ has the unit of snail/parasite and represents the number of parasites per snail

$\beta_{s}$ varies with many environmental factors, such as the density of miracidia per snail or habitat for snails, so it is hard to find a good estimate of $\beta_{s}$. However, since the main focus of this study is the thermal curve, we can approximate the shape of this thermal curve by using the percentage of infected snails from different experiments. To do that, we use a simpler model used in [47]

$\frac{dU}{dt}= -MU\beta_{s}$

$$\frac{dI}{dt}= MU\beta_{s}$$

$$\frac{dM}{dt}= -MN\beta_{s}-\mu_{m}M$$

where *U* are the number of uninfected snails, *I* the number of infected snails, *N* the total number of snails *(U+I)* and *M* the number of miracidia. After we solve this dynamic we get $\beta_{s}$

Given *M_0_* is the initial number of miracidia, *t* is the day.

*Australorbis glabratus* with *S. mansoni* [37]*.* A group of twenty to twenty-five snails exposed one miracidium each at time for 24 hours individually.

| Temperature | Infection rate (%) | $\beta_{s}$ value |
| --- | --- | --- |
| 23-25^o^C | 32 | 0.07515584 |
| 26-28^o^C | 54 | 0.4808139 |
| 31-33^o^C | 50 | 0.3922359 |

*Biomphalaria pfeifferi* with *S. mansoni* [24]*.* Snail exposed four miracidia and 30 snails were used in each experiment for 24 hours.

| Temperature | Infection rate (%) | $\beta_{s}$ value |
| --- | --- | --- |
| 22.85^o^C | 21/30 = (70) | 0.04079299 |
| 24.01^o^C | 21/30 = (70) | 0.04333207 |
| 26.26^o^C | 28/30 = (93) | 0.2203394 |
| 28.07^o^C | 24/30 = (80) | 0.08210066 |
| 30.04^o^C | 21/30 = (70) | 0.05756868 |
| 31.75^o^C | 23/30 = (76) | 0.07958718 |

*Biomphalaria pfeifferi* with *S. mansoni* [42]*.* Four to eight snails exposed ten miracia for 3 to 6 hours.

| Temperature | Infection rate (%) | $\beta_{s}$ value |
| --- | --- | --- |
| 5^o^ C | 0.5 | 0.0006344901 |
| 15^o^ C | 40 | 0.05059652 |
| 20^o^ C | 77 | 0.1629059 |
| 22.5^o^ C | 85 | 0.2249733 |

*Biomphalaria glabrata* with *S. mansoni* [40]*.* Single snail exposed five miracidia for one hour replication 30 snails.

| Temperature | Infection rate (%) | $\beta_{s}$ value |
| --- | --- | --- |
| 15^o^ C | 83 | 0.3658224 |
| 20^o^ C | 84 | 0.3865833 |
| 25^o^ C | 96 | 0.9074138 |
| 30^o^ C | 88 | 0.487644 |
| 35^o^ C | 84 | 0.4107836 |

*B. glabrata* with *S. mansoni* [48]*.* Each snail exposed to five miracidia for 2 hours.

| Temperature | Dominican Republic; Surv./%Pos | BSurv./Pos  razil; Surv./%Pos | Puerto Rico  Surv./%Pos | Venezuela  (1946)  Surv./%Pos | Venezuela  (1952)  Surv./%Pos |
| --- | --- | --- | --- | --- | --- |
| 10^0^ | 62/0  (0) | - | 88/0  (0) | 86/0  (0) | 75/0  (0) |
| 25^0^ | 142/46  (​​0.0078523) | 48/0  (0) | 76/43  (0.032993) | 82/27  (0.01388562) | 82/68  (0.07405795) |
| 35^0^ | 116/73  (0.0283053) | 52/0  (0) | 100/53  (0.0241807) | 67/36  (0.03692433) | 68/80  (0.09039597) |
| 40^0^ | 5/0  (0) | 12/0  (0) | 0/0  (0) | 12/58  (0.01246124) | 10/90  (0.00401955) |

*Biomphalari glabrata* with *S. mansoni* [49]. 30 snails individually exposed to 10 miracidia for 12 hours.

| Temperature | The percent of snails positive |
| --- | --- |
| 15^0^ | 1.3 |
| 20^0^ | 46 |
| 30^0^ | 89 |

*B. glabrata* with *S. mansoni* [39]*.* 360 snails exposed to four miracidia indivudully for 1 hours.

| Temperature | Percent of positive | $\beta_{s}$ value |
| --- | --- | --- |
| 10^0^ | 0 | 0 |
| 13^0^ | 0 | 0 |
| 16^0^ | 15 | 0.002895729 |
| 19^0^ | 21.5 | 0.004360468 |
| 22^0^ | 40.5 | 0.009807099 |
| 25^0^ | 48.6 | 0.01299096 |
| 28^0^ | 50 | 0.01373957 |
| 31^0^ | 53 | 0.01528639 |
| 34^0^ | 70.65 | 0.02722317 |
| 37^0^ | 13.43 | 0.002743025 |
| 40^0^ | 9.95 | 0.001999862 |

*Bulinus (Physopsis) globosus* with *S. haematobium* [42]*.* Four to eight snails exposed ten miracidia for 3 to 6 hours.

| Temperature | Infection rate (%) | $\beta_{s}$ value |
| --- | --- | --- |
| 5^o^ C | 1 | 0.001279126 |
| 12^o^ C | 37 | 0.04565688 |
| 19^o^ C | 64 | 0.1084776 |
| 26^o^ C | 78 | 0.1849867 |

*Bulinus globosus* infected with *S. haematobium* [19]*.* 10 snails exposed 200 miracidia for each replicated for 24 hours in each experiment.

| Temperature | Percent of infection | $\beta_{s}$ value |
| --- | --- | --- |
| 25^0^ | 87.4 | 0.03730699 |
| 20^0^ | 23.5 | 0.003512327 |
| 18^0^ | 56.4 | 0.01022082 |
| 16^0^ | 14.8 | 0.001782906 |
| 13^0^ | 21 | 0.002553961 |
| 11^0^ | 10.5 | 0.001281865 |

*Bulinus trancatus* infected with *S. haematobium* [46]*.* Two miracidia were used in each tube for two hours.

| Temperature | Number of exposed snails | Total number of snails found positive after the 50^th^ day | $\beta_{s}$ value |
| --- | --- | --- | --- |
| 10^0^ | 100 | 3 | 0.002009499 |
| 12^0^ | 50 | 3 | 0.008145561 |
| 14^0^ | 50 | 7 | 0.02029471 |
| 15^0^ | 100 | 25 | 0.02016721 |
| 20^0^ | 100 | 45 | 0.04754069 |
| 25^0^ | 150 | 71 | 0.03577785 |
| 30^0^ | 97 | 49 | 0.06475144 |
| 35^0^ | 97 | 38 | 0.04395093 |
| 38^0^ | 45 | 12 | 0.05660857 |

The top five models for *Biomphalaria* snails with *S. mansoni*

| Model | AIC value | BIC value |
| --- | --- | --- |
| Sharpeschoollow | 13.7 | 26.8 |
| Pawar | 14.7 | 24 |
| Sharpeschoolfull | 14.7 | 24 |
| Ratkowsky | 14.7 | 24.1 |
| Briere2 | 15.1 | 24.4 |
| Spain (12th in ranking) | 17 | 26.4 |

Spain curve is selected and the fitted curve to *Biomphalaria* data as follow

| 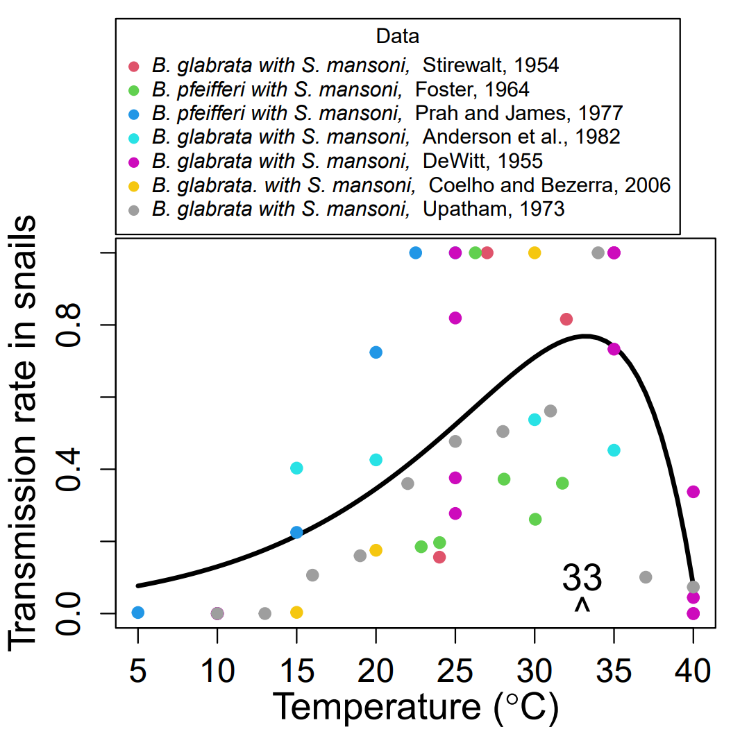 | 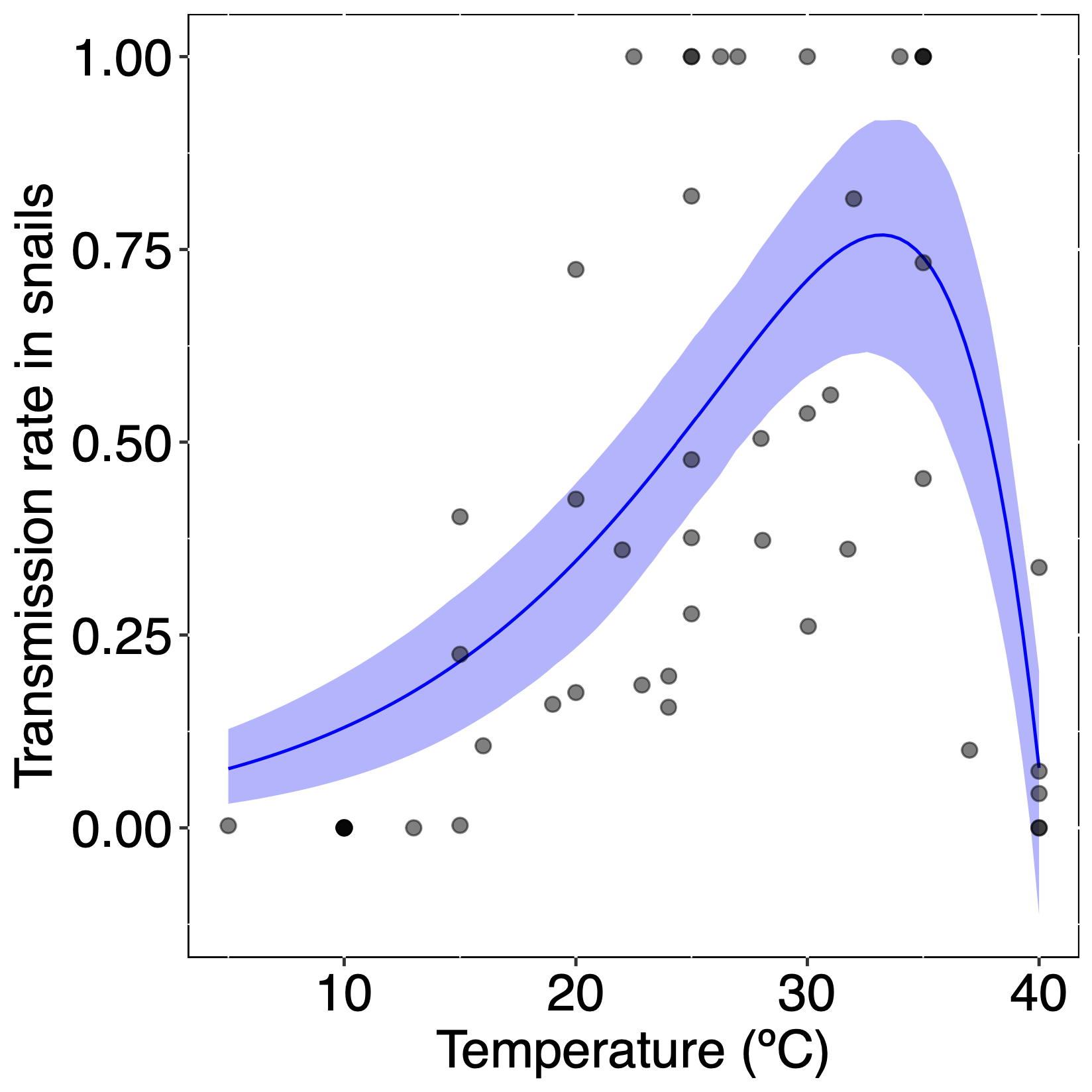 |
| --- | --- |

Fig N: Transmission rate in snails and confidence interval Biomphalaria Spp.

The top five models for *Bulinus trancatus* with *S. haematobium*

| Model | AIC value | BIC value |
| --- | --- | --- |
| Ratkowsky | -5.84 | -1.12 |
| Gaussian | -5.75 | -1.97 |
| Sharpeschoolhigh | -4.54 | 0.181 |
| Pawar | -4.54 | 0.181 |
| Weibull | -3.87 | 0.850 |
| Flin (8th in ranking) | -3.23 | 0.548 |

Flinn curve is selected and the fitted curve to *Bulinus* data as follow

| A  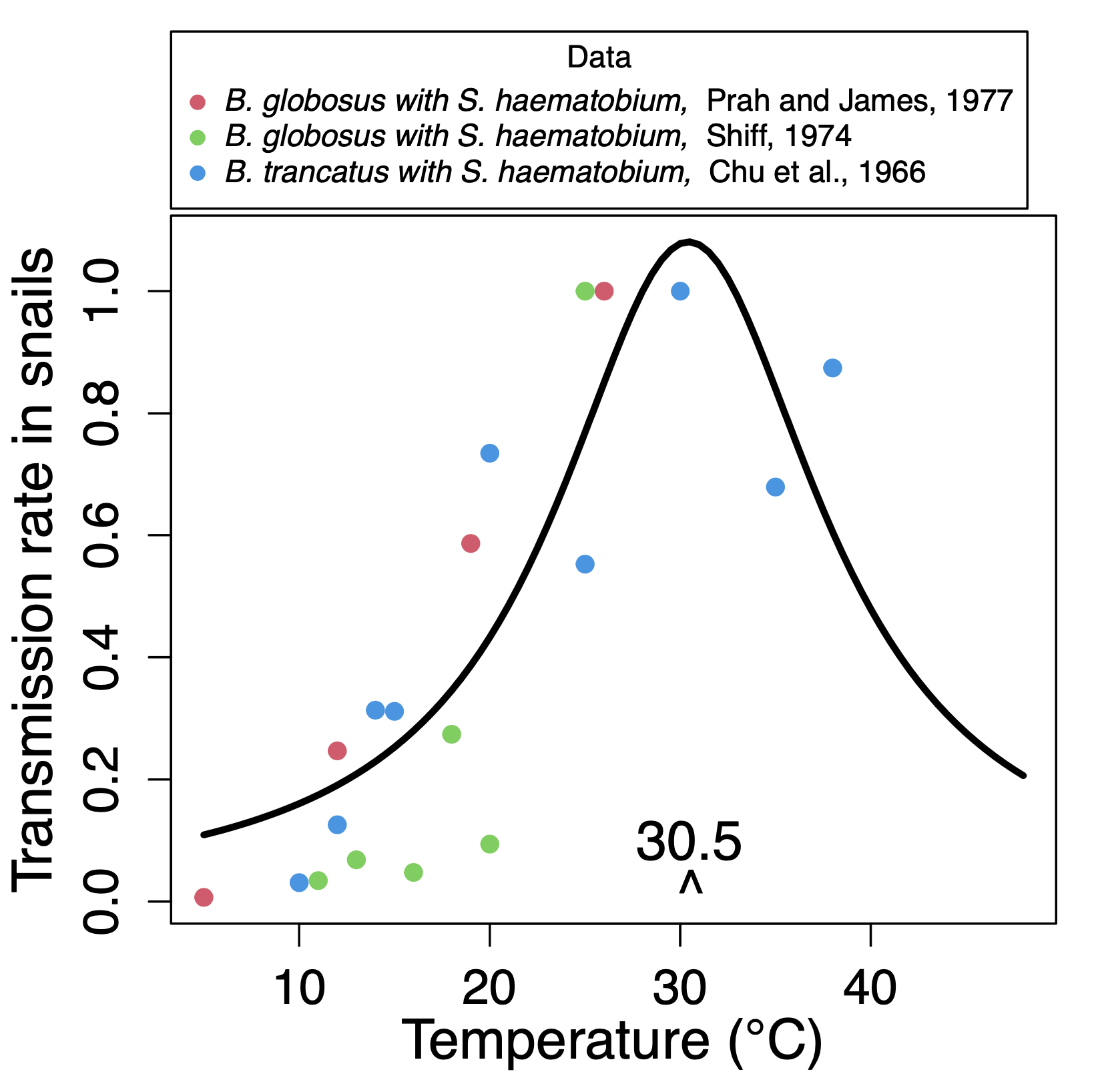 | B  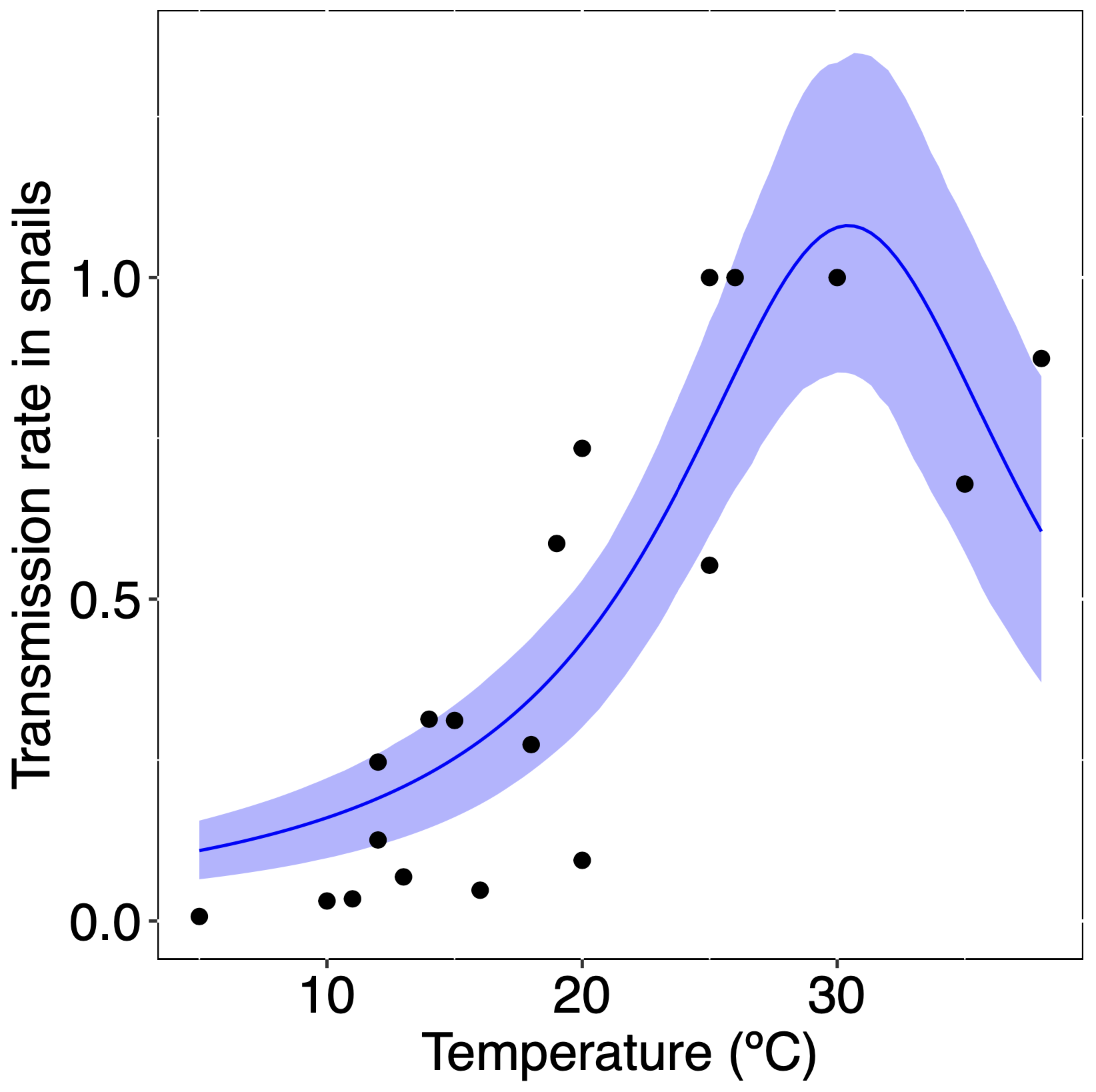 |
| --- | --- |

Fig O: Transmission rate in snails and confidence interval Bulinus Spp.

### The transmission rate of schistosomiasis in humans

$\beta_{h}$ has the unit of 1/human*day and represents the rate of parasites that penetrate human skin per day. We use a model used in [47] to adjust $\beta_{h}$

$$\frac{dC(t)}{dt}= -\beta_{h}C(t)M-\mu_{c}C(t)$$

$$\frac{dW(t)}{dt}=\beta_{h}C(t)M$$

where *C* is the number of cercariae in the water and *M* is the number of mice (which remained constant). Thus if we solve for $\beta_{h}$ we can find the transmission rate of disease in snails.

*S. mansoni* [41]*.* Six hamsters expose to 200 original cercariae for one hour.

| Temperature | 2 hours percent penetrate rate ($\beta_{h}$) | 6 hours percent penetrate rate ($\beta_{h}$) | 24 hours percent penetrate ($\beta_{h}$) |
| --- | --- | --- | --- |
| 12^o^ C | 18.5 (0.9246078) | 17.4 (1.0535) | 14.3 (2.182979) |
| 15^o^ C | 16.5 (0.810981) | 16.8 (1.002849) | 11.1 (1.523512) |
| 18^o^ C | 25.3 (1.320736) | 15.7 (0.9284408) | 12.6 (1.777458) |
| 21^o^ C | 21.4 (1.092269) | 23.2 (1.481275) | 12.4 (1.840471) |
| 24^o^ C | 14.2 (0.6998873) | 12 (0.7225294) | 5.6 (0.8305635) |
| 27^o^ C | 22.9 (1.231836) | 10.3 (0.6554545 | 3.8 (0.7054314) |
| 30^o^ C | 14.8 (0.7901292) | 16.3 (1.251547) | 4.3 (1.339291) |
| 33^o^ C | 11.1 (0.6292689) | 8.4 (0.7475422) | 0 (0) |

*S. mansoni* [50]*.* Each mouse was exposed to 50 cercariae and repeated for 100 mice. We average the number of cercariae that successfully penetrate mice’s skin for one hour.

| Temperature (°C) | The number of cercaria convert to worm | Cercarial transmission rate ($\beta_{h}$) |
| --- | --- | --- |
| 23-25 | 1610 | 0.09590499 |
| 26-28 | 2320 | 0.1552625 |

With *S. mansoni* [50]

Each mouse exposed to 67 cercaria for 30 minutes and repeated with15 mices.

| Temperature | Percentage of cercarial infected | Cercarial transmission rate ($\beta_{h}$) |
| --- | --- | --- |
| 10^0^ | ND |  |
| 15^0^ | 13.3 | 0.4618475 |
| 20^0^ | 23 | 0.8464159 |
| 25^0^ | 26.7 | 1.00836 |
| 30^0^ | 31.5 | 1.237522 |
| 35^0^ | 17 | 0.6192221 |
| 40^0^ | ND |  |
| 45^0^ | ND |  |

12 mice and average cercaria per mouse 47 for 30 minutes.

| Temperature | Percentage of cercarial infect | Cercarial transmission  rate ($\beta_{h}$) |
| --- | --- | --- |
| 10^0^ | ND |  |
| 15^0^ | 9.1 | 0.385854 |
| 20^0^ | 15.7 | 0.6910937 |
| 25^0^ | 14.5 | 0.635236 |
| 30^0^ | 21.3 | 0.9783329 |
| 35^0^ | 22.6 | 1.0652 |
| 40^0^ | 6.2 | 0.2764123 |
| 45^0^ | ND |  |

10 mice exposed to 70 cercaria in average for 30 minutes.

| Temperature | Percentage of cercarial  infect | Cercarial transmission rate ($\beta_{h}$) |
| --- | --- | --- |
| 10^0^ | 14.2 | 0.7439515 |
| 15^0^ | 30.4 | 1.76077 |
| 20^0^ | 40 | 2.484149 |
| 25^0^ | 51.2 | 3.50177 |
| 30^0^ | 55.3 | 3.9655 |
| 35^0^ | 49.4 | 3.421058 |
| 40^0^ | 13.5 | 0.7532138 |
| 45^0^ | 0 | 0 |

12 mice exposed to 76 cercaria in average for 30 minutes.

| Temperature | Percentage of cercarial infect | Cercarial transmission rate ($\beta_{h}$) |
| --- | --- | --- |
| 0^0^ | 0 | 0 |
| 5^0^ | 0 | 0 |
| 10^0^ | 2.9 | 0.1190698 |
| 15^0^ | 14.8 | 0.6479505 |
| 20^0^ | 29.2 | 1.39834 |
| 25^0^ | 30.9 | 1.50036 |
| 30^0^ | NA |  |
| 35^0^ | ND |  |
| 40^0^ | ND |  |
| 45^0^ | ND |  |

The top five models for *Biomphalaria* snails with *S. mansoni*

| Model | AIC value | BIC value |
| --- | --- | --- |
| Modifiedgaussian | 3.05 | 12.7 |
| Sharpeschoolfull | 3.83 | 17.4 |
| Quadratic | 4.38 | 12.1 |
| lrf | 5.54 | 15.2 |
| Gaussian | 5.95 | 13.7 |
| Briere (8th in ranking) | 6.86 | 16.5 |

Briere curve is selected and has the following expression

[
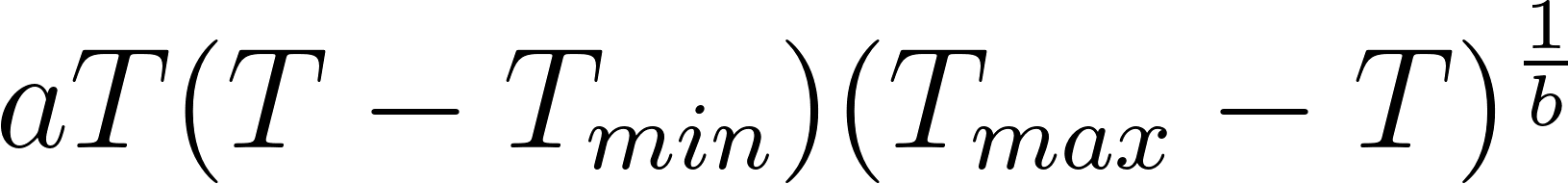
](https://www.codecogs.com/eqnedit.php?latex=aT(T-T_%7Bmin%7D)(T_%7Bmax%7D-T)%5E%5Cfrac%7B1%7D%7Bb%7D#0)

See [51] for additional information and the fitted curve to *Biomphalaria* data as follow

| 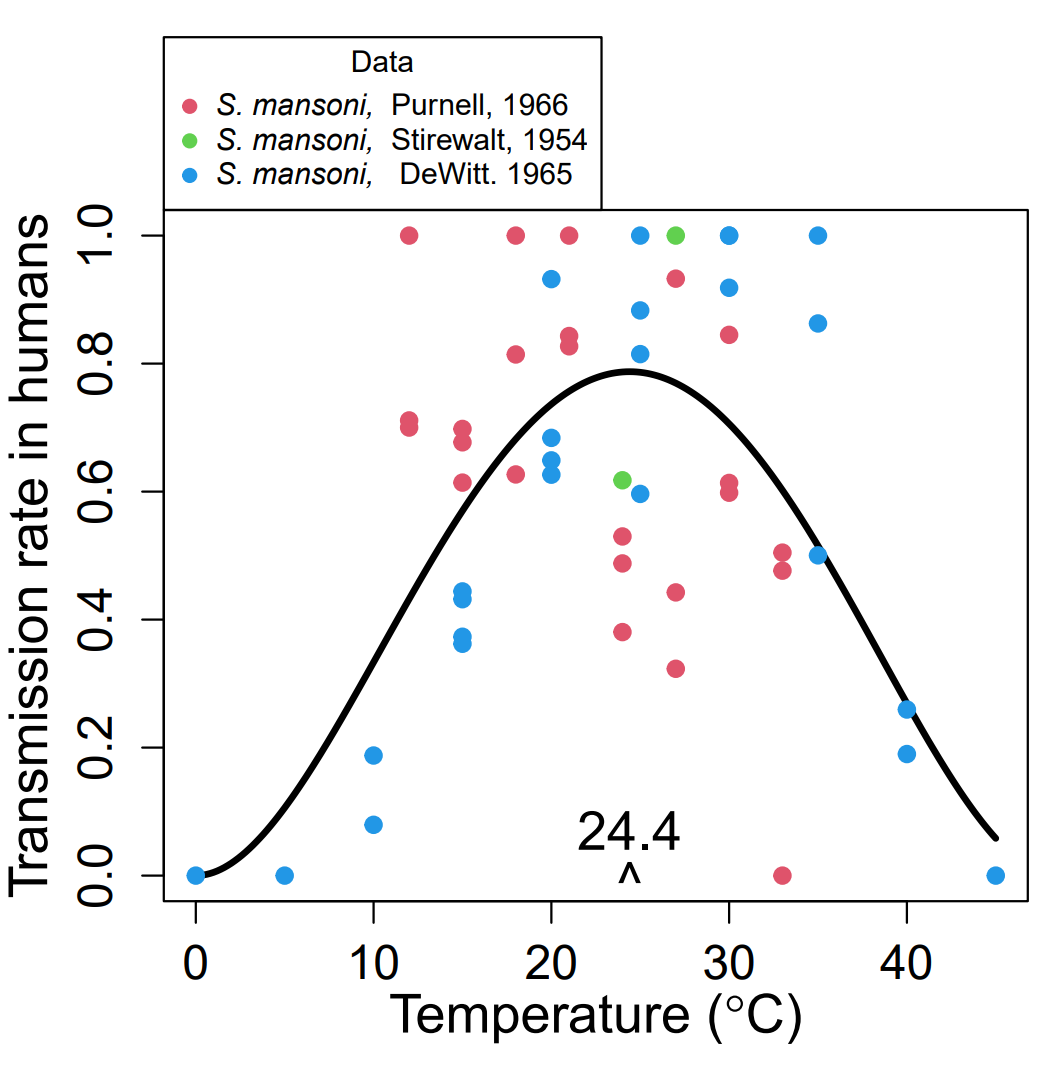 | 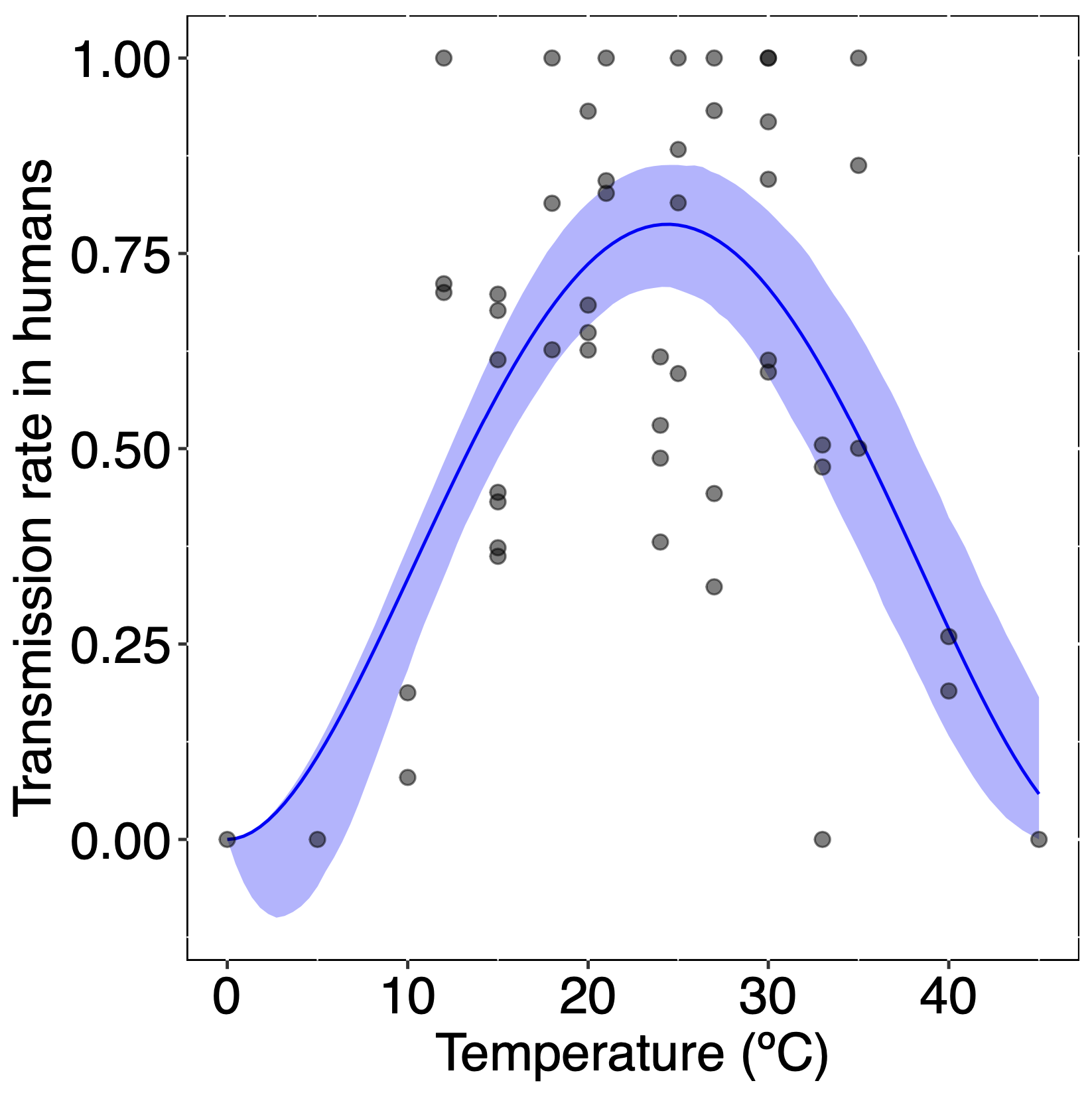 |
| --- | --- |

Fig P: Transmission rate in humans and confidence interval Biomphalaria Spp.

# Basic reproduction number

## The calculation of basic reproduction number

We use the next generation matrix method developed by [52] to calculate [
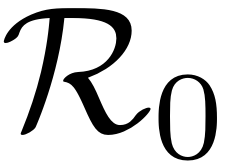
. The method gives a shortcut to draw
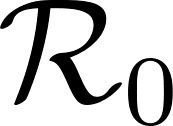
 value. First, we find the rates of new infection for all infected compartments. We call this
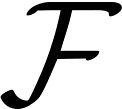
.](#D2L_inline_eq_$\mathcal{R}_0$)

$$\begin{matrix} \mathcal{F}=\left[ \begin{matrix} \lambda\left( T \right)S \\ 0 \\ \beta_{h}\left( T \right)\frac{\nu_{c}\left( T \right)}{\mu_{c}\left( T \right)}I \\ 0 \\ 0 \end{matrix} \right] \end{matrix}$$

Then, we find the rates of changes for all other compartments. We call this $\mathcal{V}$.

$$\begin{matrix} \mathcal{V}=\left[ \begin{matrix} \left( \sigma_{s}\left( T \right)+\mu_{i}\left( T \right) \right)E \\ -\sigma_{s}\left( T \right)E+\mu_{i}\left( T \right)I \\ \sigma_{p}W \\ -\sigma_{p}W+\left( \mu_{h}+\mu_{p} \right)W_{m} \\ -\left( \nu_{s}\left( T \right)-\left( S+E+I \right)\nu\right)\left( S+rE \right)+\left( \lambda\left( T \right)+\mu\left( T \right) \right)S \end{matrix} \right] \end{matrix}$$

We calculate the Jacobian matrix from new infected compartment to all infectious compartments. We call this $\mathbf{F}$.

$$\begin{matrix} \mathbf{F}=\left[ \begin{matrix} 0 & 0 & 0 & \frac{\Lambda\beta_{s}\left( T \right)h\delta_{e}\left( T \right)\nu_{e}S}{\mu_{m}\left( T \right)\left( S+E+I \right)} \\ 0 & 0 & 0 & 0 \\ 0 & \frac{\beta_{h}\left( T \right)\nu_{c}\left( T \right)}{\mu_{c}\left( T \right)} & 0 & 0 \\ 0 & 0 & 0 & 0 \end{matrix} \right] \end{matrix}$$

We calculate the Jacobian of matrix from all infectious compartment to all infectious compartments. We call this $\mathbf{V}$.

$$\begin{matrix} \mathbf{V}=\left[ \begin{matrix} \sigma_{s}\left( T \right)+\mu_{i}\left( T \right) & 0 & 0 & 0 \\ -\sigma_{s}\left( T \right) & \mu_{i}\left( T \right) & 0 & 0 \\ 0 & 0 & \sigma_{p} & 0 \\ 0 & 0 & -\sigma_{p} & \mu_{h}+\mu_{p} \end{matrix} \right] \end{matrix}$$

We need to calculate the inverse of $\mathbf{V}$ to find the next generation matrix.

$$\begin{matrix} \mathbf{V}^{\mathbf{-}\mathbf{1}}=\left[ \begin{matrix} \frac{1}{\sigma_{s}\left( T \right)+\mu_{i}\left( T \right)} & 0 & 0 & 0 \\ \frac{\sigma_{s}\left( T \right)}{\mu_{i}\left( T \right)\left( \sigma_{s}\left( T \right)+\mu_{i}\left( T \right) \right)} & \frac{1}{\mu_{i}\left( T \right)} & 0 & 0 \\ 0 & 0 & \frac{1}{\sigma_{p}} & 0 \\ 0 & 0 & \frac{1}{\mu_{h}+\mu_{p}} & \frac{1}{\mu_{h}+\mu_{p}} \end{matrix} \right] \end{matrix}$$

The product of $\mathbf{F}\mathbf{V}^{\mathbf{-1}}$ at DFE equilibrium gives us the next generation matrix and the spectral radios of the next generation matrix equals to a threshold value of the basic reproduction number $\mathcal{R}_{0}$

$$\begin{matrix} \mathbf{F}\mathbf{V}^{\mathbf{-}\mathbf{1}} & \left( \mathbf{X}^{\mathbf{*}} \right) \\ = & \left[ \begin{matrix} 0 & 0 & \frac{\Lambda\beta_{s}\left( T \right)h\delta_{e}\left( T \right)\nu_{e}S^{*}}{\mu_{m}\left( T \right)\left( \mu_{h}+\mu_{p} \right)S^{*}} & \frac{\Lambda\beta_{s}\left( T \right)h\delta_{e}\left( T \right)\nu_{e}S^{*}}{\mu_{m}\left( T \right)\left( \mu_{h}+\mu_{p} \right)S^{*}} \\ 0 & 0 & 0 & 0 \\ \frac{\beta_{h}\left( T \right)\nu_{c}\left( T \right)\sigma_{s}\left( T \right)}{\mu_{c}\left( T \right)\mu_{i}\left( T \right)\left( \sigma_{s}\left( T \right)+\mu_{i}\left( T \right) \right)} & \frac{\beta_{h}\left( T \right)\nu_{c}\left( T \right)}{\mu_{c}\left( T \right)\mu_{i}\left( T \right)} & 0 & 0 \\ 0 & 0 & 0 & 0 \end{matrix} \right] \end{matrix}$$

Note we evaluate the next generation matrix at the DFE $X^{*}=\left( S^{*},0,0,0,0 \right)$. We can find a disease free equilibrium (DFE) by simple setting the right hand side of the Ordinary differential equation (ODE) system to zero and plugging the DFE. Therefore,

$$\begin{matrix} \left( \nu_{s}\left( T \right)-\mu\left( T \right)-S^{*}\nu\right)S^{*}=0\Rightarrow S^{*}=\frac{\nu_{s}\left( T \right)-\mu\left( T \right)}{\nu} \end{matrix}$$

The DFE is

$$\begin{matrix} \left( S^{*},E^{*},I^{*},W^{*},W_{m}^{*} \right)=\left( \frac{\nu_{s}\left( T \right)-\mu\left( T \right)}{\nu},0,0,0,0 \right) \end{matrix}$$

Calculate the spectral radios of the next generation matrix we get the basic reproduction number $\mathcal{R}_{0}$. Note that we assume $\nu_{s}\left( T \right)-\mu\left( T \right)>0$ in order to biologically be mean-full.

$$\begin{matrix} \mathbf{F}\mathbf{V}^{\mathbf{-}\mathbf{1}}\left( \mathbf{X}^{\mathbf{*}} \right)=\mathcal{R}_{0}=\left( \frac{\Lambda\beta_{s}\left( T \right)h\delta_{e}\left( T \right)\nu_{e}\beta_{h}\left( T \right)\nu_{c}\left( T \right)\sigma_{s}\left( T \right)}{\mu_{m}\left( T \right)\left( \mu_{h}+\mu_{p} \right)\mu_{c}\left( T \right)\mu_{i}\left( T \right)\left( \sigma_{s}\left( T \right)+\mu_{i}\left( T \right) \right)} \right)^{1/2} \end{matrix}$$

| 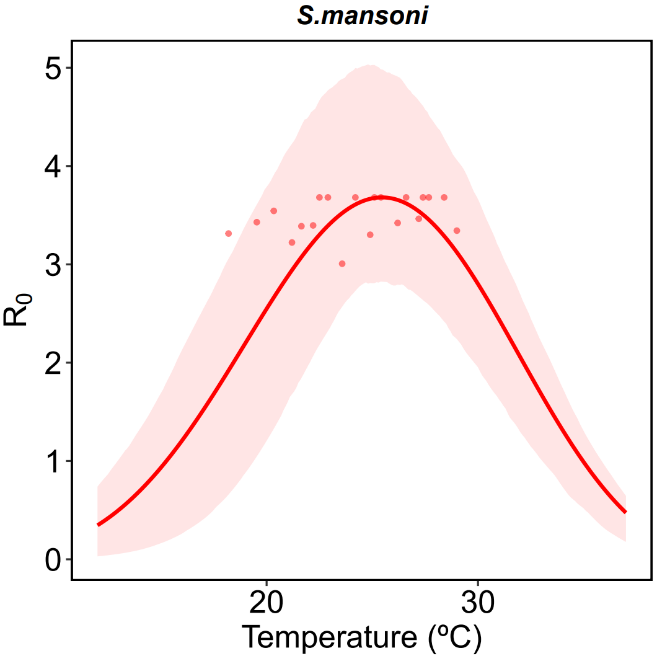 | ›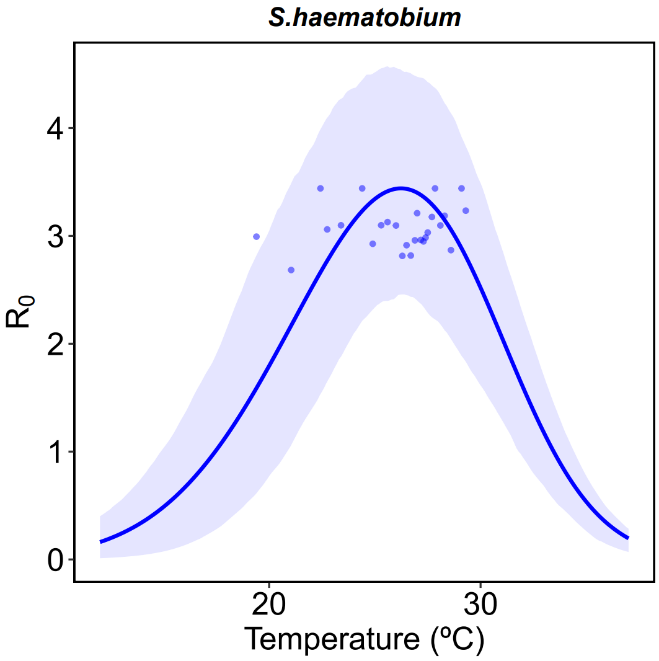 |
| --- | --- |

›

Fig Q : Simulation of basic reproduction number for S. mansoni (left) and S. haematobium (right)

# Model comparison with data

The prevalence data was pulled from the Global Neglected tropical disease (GNTD) database which is the percentage of infection in humans for nearly 11 thousand location locations of Africa for *S. mansoni* and *S. haematobium*. We also draw the bioclimatic variable from [53] for each location in GNTD data set and then created a data set of prevalence in humans and temperature for the locations in Africa. We subset the data to only include non-zero data points to focus on areas where schistosomiasis is endemic

## GNTD prevalence data and temperature

We fit a quantile GAM regression using the qgam R package to explore the relationship between temperature and schistosomiasis prevalence.

### Basic reproduction number as a predictor of prevalence

We used quantile regressions to evaluate our $R_{0}$ estimate as a predictor of prevalence. We found that our estimate of $R_{0}$ is a significant predictor of prevalence for all quantiles considered ($\tau=$0.25,0.5,0.75, 0.9, 0.95, 0.98, p-value < 0.005 for all, Figure 18). The R0 estimate from Nguyen et al. was not a significant predictor of prevalence, except for the 25th quantile. In addition, the model using our R0 estimate had a lower AICc (AIC(Aslan et al) = 3240, AIC(Nguyen et al) = 3297, $\tau=$ 0.5).


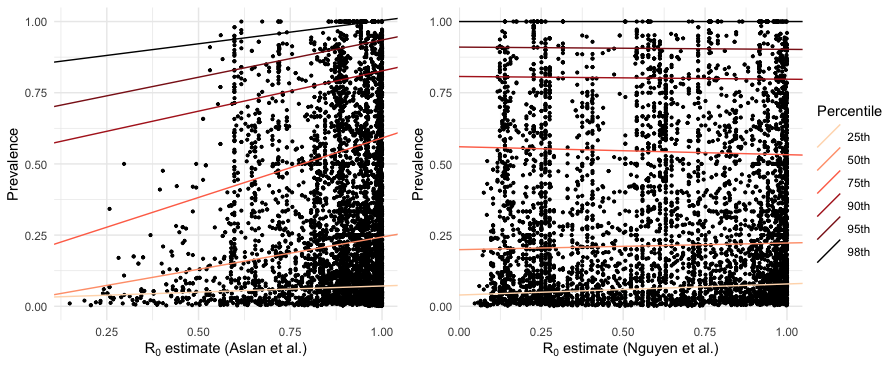


Fig R: Quantile regression of prevalence over $R_{0}$ estimates for our estimate and for Nguyen et al. estimate.

# Masking for non-suitable regions for schistosomiasis

We created a mask to remove areas that are non-suitable for schistosomiasis transmission with 25 km is away from permanent or temporary water bodies and the extremely low human population density. Accordingly, this mask is based on two sources of input data: the Global Surface Water (GSW) Maximum Extent (30x30 m resolution) and the Gridded Population of the World (GPW), version 4, Population Density for 2020 (1x1 km resolution). The GSW Maximum Extent data identifies locations where surface water was detected at least once in the 38-year Landsat observation period (1984-2021), providing a conservative estimate of the water bodies potentially suitable for schistosomiasis transmission at 30m resolution. GSW data was mosaic and reprojected to WGS 1984. ArcGIS Pro v3.2.0 Distance Accumulation tool was used to derive a binary mask of locations in Africa within 25km of surface water and outside. The GPW data was similarly used to derive a binary mask removing areas that have less than 2 people per square kilometer. The intersection of the two maps was retained as potentially suitable habitat [54,55].

# Human water contact rate

We formulate water contact rate for varying temperature by using a sigmoid function as follow

$C_{w}(T) = \frac{1}{1+e^{-a(T-T_{med})}} + b$

where: $b\geq0$ is the minimum contact rate at low temperature, $T_{med}$ is the temperature at which the slope of mid-point occurs, and $a\geq0$ is the parameter that governs the steepness of the function. The following plot show the simulations of water contact rate for three different scenarios.


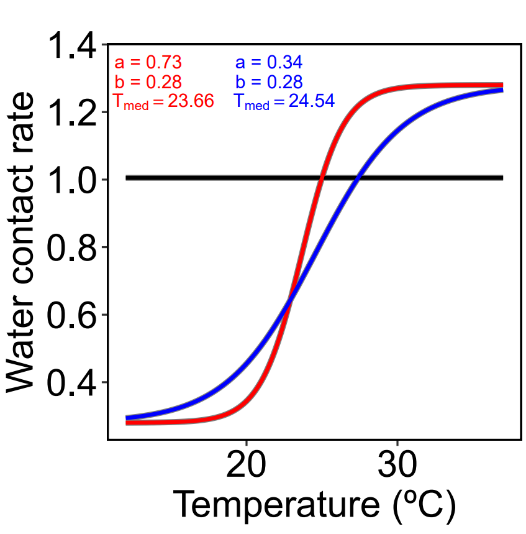


Fig S: Blue and red color show when the water contact rate is non constant function of temperature and black color shows when the water contact rate is constant and equals to 1 for all temperature.

# Sensitivity

In this section we discuss a sensitivity analysis method by taking the partial derivative of basic reproduction number $R_{0}$

$$\begin{matrix} \frac{d\mathcal{R}_{0}}{dT}=\frac{\partial\mathcal{R}_{0}}{\partial x_{1}}\frac{\partial x_{1}}{\partial T}+...+\frac{\partial\mathcal{R}_{0}}{\partial x_{n}}\frac{\partial x_{n}}{\partial T} \end{matrix}$$

if we are interested in sensitivity of a certain parameters. We can simple assume all other parameter are constant, so

$$\begin{matrix} \frac{d\mathcal{R}_{0}}{dT}=\frac{\partial\mathcal{R}_{0}}{\partial x_{i}}\frac{\partial x_{i}}{\partial T} \end{matrix}$$

In order to find the sensitivity of $\mathcal{R}_{0}$ for each parameter we need to take derivative of the parameter respect to temperature and also derivative of $\mathcal{R}_{0}$ with respect to the corresponding parameter and multiple with each other. Thus, we need to take derivative of thermal performance function with respect to temperature.

## The derivative of each thermal performance function

### Gaussian

The explicit equation is

$$\begin{matrix} G\left( T \right)=r_{max}e^{-0.5\left( \frac{T-T_{opt}}{a} \right)^{2}} \end{matrix}$$

Then the derivative would be

$$\begin{matrix} \frac{dG\left( T \right)}{dT}=\frac{-r_{max}\left( T-T_{opt} \right)}{a^{2}}e^{-0.5\left( \frac{T-T_{opt}}{a} \right)^{2}} \end{matrix}$$

### Spain

The explicit equation is

$$\begin{matrix} S\left( T \right)=r_{0}e^{aT}\left( 1-be^{cT} \right) \end{matrix}$$

Then the derivative would be

$$\begin{matrix} \frac{dS\left( T \right)}{dT}=r_{0}e^{aT}\left( a-abe^{cT}-bce^{cT} \right) \end{matrix}$$

### Filnn

The explicit equation is

$$\begin{matrix} F\left( T \right)=\frac{1}{1+a+bT+cT^{2}} \end{matrix}$$

Then the derivative would be

$$\begin{matrix} \frac{dF\left( T \right)}{dT}=\frac{-b-2cT}{\left( 1+a+bT+cT^{2} \right)^{2}} \end{matrix}$$

### Thomas

The explicit equation is

$$\begin{matrix} T\left( T \right)=ae^{bT}-\left( c+de^{eT} \right) \end{matrix}$$

Then the derivative would be

$$\begin{matrix} \frac{dT\left( T \right)}{dT}=abe^{bT}-dee^{eT} \end{matrix}$$

### Quadratic

The explicit equation is

$$\begin{matrix} Q\left( T \right)=a+bT+cT^{2} \end{matrix}$$

Then the derivative would be

$$\begin{matrix} \frac{dQ\left( T \right)}{dT}=b+2cT \end{matrix}$$

### Briere2

The explicit equation is

$$\begin{matrix} B\left( T \right)=aT\left( T-T_{min} \right)\left( T_{max}-T \right)^{\frac{1}{b}} \end{matrix}$$

Then the derivative would be

$$\begin{matrix} \frac{dB\left( T \right)}{dT}=a\left( T-T_{min} \right)\left( T_{max}-T \right)^{\frac{1}{b}}+aT\left( T_{max}-T \right)^{\frac{1}{b}}+\frac{-aT}{b}\left( T-T_{min} \right)\left( T_{max}-T \right)^{\frac{1-b}{b}} \end{matrix}$$

### Johnson Lewin

The explicit equation is

$$J\left( T \right)= \frac{r_{0}e^{\frac{-e}{k*(T+273.15)}}}{1+e^{-\frac{e_{h}-\left( \frac{e_{h}}{{(T}_{opt}+273.15)}+k*\ln\left( \frac{e}{e_{h}-e} \right) \right)*(T+273.15)}{k*(T+273.15)}}}$$

Since this function is not in use of $R_{0}$, we do not have to take the partial derivative.

## The partial derivative of each parameter

Now, let calculate the partial derivative for each parameter function by assuming all other parameters are constant

### Transmission rate in human $\boldsymbol{\beta}_{\boldsymbol{h}}\left( \boldsymbol{T} \right)$

The curve was modeled with briere2 function whose derivative given above, thus

$$\begin{matrix} \frac{d\mathcal{R}_{0}}{dT} & =\frac{\partial\mathcal{R}_{0}}{\partial\beta_{h}\left( T \right)}\frac{\partial\beta_{h}\left( T \right)}{dT}=\frac{\mathcal{R}_{0}}{2\beta_{h}\left( T \right)}\frac{\partial\beta_{h}\left( T \right)}{dT} \\ = & \frac{\mathcal{R}_{0}}{2}*\left( \frac{1}{T}+\frac{1}{T-T_{min}}-\frac{1}{b\left( T_{max}-T \right)} \right) \end{matrix}$$

### Transmission rate in snail $\boldsymbol{\beta}_{\boldsymbol{s}}\left( \boldsymbol{T} \right)$

The curve was modeled with spain function whose derivative given above, thus

$$\begin{matrix} \frac{d\mathcal{R}_{0}}{dT} & =\frac{\partial\mathcal{R}_{0}}{\partial\beta_{s}\left( T \right)}\frac{\partial\beta_{s}\left( T \right)}{dT}=\frac{\mathcal{R}_{0}}{2\beta_{s}\left( T \right)}\frac{\partial\beta_{s}\left( T \right)}{dT} \\ = & \frac{\mathcal{R}_{0}}{2r_{0}e^{aT}\left( 1-be^{cT} \right)}r_{0}e^{aT}\left( a-abe^{cT}-bce^{cT} \right) \\ = & \frac{\mathcal{R}_{0}\left( a-abe^{cT}-bce^{cT} \right)}{2\left( 1-be^{cT} \right)} \end{matrix}$$

### Miracidia hatching rate $\boldsymbol{\delta}_{\boldsymbol{e}}\left( \boldsymbol{T} \right)$

The curve was modeled with flinn function whose derivative given above, thus

$$\begin{matrix} \frac{d\mathcal{R}_{0}}{dT} & =\frac{\partial\mathcal{R}_{0}}{\partial\delta_{e}\left( T \right)}\frac{\partial\delta_{e}\left( T \right)}{dT}=\frac{\mathcal{R}_{0}}{2\delta_{e}\left( T \right)}\frac{\partial\delta_{e}\left( T \right)}{dT} \\ = & \frac{\mathcal{R}_{0}}{2\delta_{e}\left( T \right)}\frac{-b-2cT}{\left( 1+a+bT+cT^{2} \right)^{2}} \\ = & \frac{-\mathcal{R}_{0}\left( b+2cT \right)}{2\left( 1+a+bT+cT^{2} \right)} \end{matrix}$$

### Cercarial releasing rate $\boldsymbol{\nu}_{\boldsymbol{c}}\left( \boldsymbol{T} \right)$

The curve was modeled with Gaussian function whose derivative given above, thus

$$\begin{matrix} \frac{d\mathcal{R}_{0}}{dT} & =\frac{\partial\mathcal{R}_{0}}{\partial\nu_{c}\left( T \right)}\frac{\partial\nu_{c}\left( T \right)}{dT}=\frac{\mathcal{R}_{0}}{2\nu_{c}\left( T \right)}\frac{\partial\nu_{c}\left( T \right)}{dT} \\ = & \frac{\mathcal{R}_{0}}{2\nu_{c}\left( T \right)}\frac{-r_{max}\left( T-T_{opt} \right)}{a^{2}}e^{-0.5\left( \frac{T-T_{opt}}{a} \right)^{2}} \\ = & \frac{-\mathcal{R}_{0}\left( T-T_{opt} \right)}{2a^{2}} \end{matrix}$$

### Prepatent period $\boldsymbol{\sigma}_{\boldsymbol{s}}\left( \boldsymbol{T} \right)$

The curve was modeled with Gaussian function whose derivative given above, thus

$$\begin{matrix} \frac{d\mathcal{R}_{0}}{dT} & =\frac{\partial\mathcal{R}_{0}}{\partial\sigma_{s}\left( T \right)}\frac{\partial\sigma_{s}\left( T \right)}{dT} \\ = & \frac{\mathcal{R}_{0}}{2}...... \end{matrix}$$

### Mortality rate of miracidia, $\boldsymbol{\mu}_{\boldsymbol{m}}\left( \boldsymbol{T} \right)$

The curve was modeled with Thomas function whose derivative given above, thus

$$\begin{matrix} \frac{d\mathcal{R}_{0}}{dT} & =\frac{\partial\mathcal{R}_{0}}{\partial\mu_{m}\left( T \right)}\frac{\partial\mu_{m}\left( T \right)}{dT}=-\frac{\mathcal{R}_{0}}{2\mu_{m}\left( T \right)}\frac{\partial\mu_{m}\left( T \right)}{dT} \\ = & -\frac{\mathcal{R}_{0}}{2\mu_{m}\left( T \right)}\left( abe^{bT}-dee^{eT} \right) \\ = & \frac{\mathcal{R}_{0}\left( -abe^{bT}+dee^{eT} \right)}{2\left( ae^{bT}-\left( c+de^{eT} \right) \right)} \end{matrix}$$

### Mortality rate of cercaria, $\boldsymbol{\mu}_{\boldsymbol{c}}\left( \boldsymbol{T} \right)$

The curve was modeled with Spain function whose derivative given above, thus

$$\begin{matrix} \frac{d\mathcal{R}_{0}}{dT} & =\frac{\partial\mathcal{R}_{0}}{\partial\mu_{c}\left( T \right)}\frac{\partial\mu_{c}\left( T \right)}{dT}=-\frac{\mathcal{R}_{0}}{2\mu_{c}\left( T \right)}\frac{\partial\mu_{c}\left( T \right)}{dT} \\ = & -\frac{\mathcal{R}_{0}}{2r_{0}e^{aT}\left( 1-be^{cT} \right)}r_{0}e^{aT}\left( a-abe^{cT}-bce^{cT} \right) \\ = & -\frac{\mathcal{R}_{0}\left( a-abe^{cT}-bce^{cT} \right)}{2\left( 1-be^{cT} \right)} \end{matrix}$$

### Mortality rate of infected snails, $\boldsymbol{\mu}_{\boldsymbol{i}}\left( \boldsymbol{T} \right)$

The curve was modeled with flinn function whose derivative given above, thus

$$\begin{matrix} \frac{d\mathcal{R}_{0}}{dT} & =\frac{\partial\mathcal{R}_{0}}{\partial\mu_{i}\left( T \right)}\frac{\partial\mu_{i}\left( T \right)}{dT}=\frac{\mathcal{R}_{0}}{2}\frac{-\left( \sigma_{s}\left( T \right)+2\mu_{i}\left( T \right) \right)}{\mu_{i}\left( T \right)\left( \sigma_{s}\left( T \right)+\mu_{i}\left( T \right) \right)}\frac{\partial\mu_{i}\left( T \right)}{dT} \\ = & \frac{\mathcal{R}_{0}}{2}\frac{-\left( \sigma_{s}\left( T \right)+2\mu_{i}\left( T \right) \right)}{\mu_{i}\left( T \right)\left( \sigma_{s}\left( T \right)+\mu_{i}\left( T \right) \right)}\frac{-b-2cT}{\left( 1+a+bT+cT^{2} \right)^{2}} \\ = & \frac{\mathcal{R}_{0}\left( \sigma_{s}\left( T \right)+2\mu_{i}\left( T \right) \right)\left( b+2cT \right)}{2\mu_{i}\left( T \right)\left( \sigma_{s}\left( T \right)+\mu_{i}\left( T \right) \right)\left( 1+a+bT+cT^{2} \right)^{2}} \end{matrix}$$

# References

1. Hailegebriel T, Nibret E, Munshea A. Prevalence of Schistosoma mansoni and S. haematobium in snail intermediate hosts in Africa: a systematic review and meta-analysis. Journal of tropical medicine. 2020;2020.

2. Kalinda C, Mushayabasa S, Chimbari MJ, Mukaratirwa S. Optimal control applied to a temperature dependent schistosomiasis model. Biosystems. 2019;175:47–56.

3. Sokolow SH, Huttinger E, Jouanard N, Hsieh MH, Lafferty KD, Kuris AM, et al. Reduced transmission of human schistosomiasis after restoration of a native river prawn that preys on the snail intermediate host. Proceedings of the National Academy of Sciences. 2015;112(31):9650–5.

4. Gurarie D, Lo NC, Ndeffo-Mbah ML, Durham DP, King CH. The human-snail transmission environment shapes long term schistosomiasis control outcomes: Implications for improving the accuracy of predictive modeling. PLoS neglected tropical diseases. 2018;12(5):e0006514.

5. CHEEVER A, MACEDONIA J. MOSHMANN. 1 E. & CHEEVER, EA-Kinetics of egg production and egg excretion by Schistosoma mansoni and Schistosoma japonicum in mice infected with a single pair of worms. Amcr. J trop Med Hyg. 1994;50:281–95.

6. Castillo-Chavez C, Feng Z, Xu D. A schistosomiasis model with mating structure and time delay. Mathematical biosciences. 2008;211(2):333–41.

7. Fulford A, Butterworth A, Ouma J, Sturrock R. A statistical approach to schistosome population dynamics and estimation of the life-span of Schistosoma mansoni in man. Parasitology. 1995;110(3):307–16.

8. Daniel P, Hannah O. rTPC: Functions for Fitting Thermal Performance Curves [Internet]. Available from: https://padpadpadpad.github.io/rTPC/

9. Kot M. Elements of mathematical ecology. Cambridge University Press; 2001.

10. McCreesh N, Arinaitwe M, Arineitwe W, Tukahebwa EM, Booth M. Effect of water temperature and population density on the poulation dynamics of Schistosoma mansoni intermediate host snails. Parasites & Vectors. 2014;7:1–9.

11. El-Hassan AA. Laboratory studies on the direct effect of temperature on Bulinus truncatus and Biomphalaria alexandrina, the snail intermediate hosts of schistosomes in Egypt. Folia Parasitol (Praha). 1974;21(2):181–7.

12. Kock KN de, Eeden JA van. Effect of programmed circadian temperature fluctuations on population dynamics of Biomphalaria pfeifferi (Krauss). African Zoology. 1986;21(1):28–32.

13. Appleton CC. The influence of temperature on the life-cycle and distribution of Biomphalaria pfeifferi (Krauss, 1948) in South-Eastern Africa. International Journal for Parasitology. 1977 Oct;7(5):335–45.

14. Sturrock R. The influence of temperature on the biology of Biomphalaria pfeifferi (Krauss), an intermediate host of Schistosoma mansoni. Annals of Tropical Medicine & Parasitology. 1966;60(1):100–5.

15. Sturrock R, Sturrock B. The influence of temperature on the biology of Biomphalaria glabrata (Say), intermediate host of Schistosoma mansoni on St. Lucia, West Indies. Annals of Tropical Medicine & Parasitology. 1972;66(3):385–90.

16. Michelson EH, others. The effects of temperature on growth and reproduction of Australorbis glabratus in the laboratory. American Journal of Hygiene. 1961;73(1):66–74.

17. Shiff C. The influence of temperature on the intrinsic rate of naturol increase of the freshwater snail, Biomphalaria pfeifferi (Krauss)(Pulmonata: Planorbidae). Arch Hydrobiol. 1967;62:429–38.

18. Kalinda C, Chimbari MJ, Mukaratirwa S. Effect of temperature on the Bulinus globosus—Schistosoma haematobium system. Infectious Diseases of Poverty. 2017;6(1):1–7.

19. Shiff C. Studies on Bulinus (Physopsis) Globosus in Rhodesia. Annalsof Tropical Medicine & Parasitology. 1964;58(1).

20. Kubirizajournal GK, Madsen H, Likongwe JS, Stauffer Jr JR, OmbeJeremiah K𠆘, Kapute F. Effect of temperature on growth, survival and reproduction of Bulinus nyassanus (Smith, 1877)(Mollusca: Gastropoda) from Lake Malawi. African Zoology. 2010;45(2):315–20.

21. Lynch M, Gabriel W. Environmental tolerance. The American Naturalist. 1987;129(2):283–303.

22. Johnson FH, Lewin I. The growth rate of E. coli in relation to temperature, quinine and coenzyme. Journal of Cellular and Comparative Physiology. 1946;28(1):47–75.

23. Joubert P, Pretorius S, De Kock K, Van Eeden J. Survival of Bulinus africanus (Krauss), Bulinus globosus (Morelet) and Biomphalaria pfeifferi (Krauss) at constant high temperatures. African Zoology. 1986;21(1):85–8.

24. Foster R, others. The effect of temperature on the development of Schistosoma mansoni Sambon 1907 in the intermediate host. Journal of Tropical Medicine and Hygiene. 1964;67(12):289–92.

25. El-Emam M, Madsen H. The effect of temperature, darkness, starvation and various food types on growth, survival and reproduction of Helisoma duryi, Biomphalaria alexandrina and Bulinus truncatus (Gastropoda: Planorbidae). Hydrobiologia. 1982;88:265–75.

26. Cunningham P. Basic Microcomputer Models in Biology: by James D. Spain Addison-Wesley; Reading MA, 1982 xiv+ 354 pages. $23.50;\pounds 15.50. Wiley Online Library; 1983.

27. Montagnes DJ, Morgan G, Bissinger JE, Atkinson D, Weisse T. Short-term temperature change may impact freshwater carbon flux: a microbial perspective. Global Change Biology. 2008;14(12):2823–38.

28. Pfluger W. Experimental epidemiology of schistosomiasis. Zeitschrift fur Parasitenkunde. 1981;63(2).

29. Pflüger W. Experimental epidemiology of schistosomiasis: I. The prepatent period and cercarial production of Schistosoma mansoni in Biomphalaria snails at various constant temperatures. Zeitschrift für Parasitenkunde. 1980;63:159–69.

30. Gordon R, Davey T, Peaston H. The transmission of human bilharziasis in Sierra Leone, with an account of the life-cycle of the schistosomes concerned, S. mansoni and S. haematobium. Annals of Tropical Medicine & Parasitology. 1934;28(3):323–418.

31. Pflüger W, Roushdy M, Emam ME. The prepatent period and cercarial production of Schistosoma haematobium in Bulinus truncatus (Egyptian field strains) at different constant temperatures. Zeitschrift für Parasitenkunde. 1984;70:95–103.

32. Yang GJ, Utzinger J, Sun LP, Hong QB, Vounatsou P, Tanner M, et al. Effect of temperature on the development of Schistosoma japonicum within Oncomelania hupensis, and hibernation of O. hupensis. Parasitology Research. 2007;100:695–700.

33. Nguyen KH, Boersch-Supan PH, Hartman RB, Mendiola SY, Harwood VJ, Civitello DJ, et al. Interventions can shift the thermal optimum for parasitic disease transmission. Proceedings of the National Academy of Sciences. 2021;118(11):e2017537118.

34. Samuelson JC, Quinn JJ, Caulfield JP. Hatching, chemokinesis, and transformation of miracidia of Schistosoma mansoni. The Journal of parasitology. 1984;321–31.

35. Flinn P. Temperature-dependent functional response of the parasitoid Cephalonomia waterstoni (Gahan)(Hymenoptera: Bethylidae) attacking rusty grain beetle larvae (Coleoptera: Cucujidae). Environmental Entomology. 1991;20(3):872–6.

36. Fried B, LaTerra R, Kim Y. Emergence of cercariae of Echinostoma caproni and Schistosoma mansoni from Biomphalaria glabrata under different laboratory conditions. Journal of helminthology. 2002;76(4):369–71.

37. Stirewalt MA. Effect of snail maintenance temperatures on development of Schistosoma mansoni. Experimental Parasitology. 1954;3(6):504–16.

38. Schreiber FG, Schubert M. Experimental infection of the snail Australorbis glabratus with the trematode Schistosoma mansoni and the production of cercariae. The Journal of parasitology. 1949;35(1):91–100.

39. Upatham ES, others. The effect of water temperature on the penetration and development of St. Lucian Schistosoma mansoni miracidia in local Biomphalaria glabrata. Southeast Asian J Trop Med Public Health. 1973;4(3):367–70.

40. Anderson RM, Mercer JG, Wilson RA, Carter NP. Transmission of *Schistosoma mansoni* from man to snail: experimental studies of miracidial survival and infectivity in relation to larval age, water temperature, host size and host age. Parasitology. 1982 Oct;85(2):339–60.

41. Purnell R. Host-parasite relationships in schistosomiasis: III.—The effect of temperature on the survival of Schistosoma monsoni miracidia and on the survival and infectivity of Schistosoma mansoni cercariae. Annals of Tropical Medicine & Parasitology. 1966;60(2):182–6.

42. Prah S, James C. The influence of physical factors on the survival and infectivity of miracidia of Schistosoma mansoni and S. haematobium I. Effect of temperature and ultra-violet light. Journal of Helminthology. 1977;51(1):73–85.

43. Thomas MK, Aranguren-Gassis M, Kremer CT, Gould MR, Anderson K, Klausmeier CA, et al. Temperature–nutrient interactions exacerbate sensitivity to warming in phytoplankton. Global change biology. 2017;23(8):3269–80.

44. Lawson JR, Wilson R. The survival of the cercariae of Schistosoma mansoni in relation to water temperature and glycogen utilization. Parasitology. 1980;81(2):337–48.

45. Ghandour A. A study of the relationship between temperature and the infectivity of Schistosoma mansoni and Schistosoma haematobium cercariae. Journal of Helminthology. 1976;50(3):193–6.

46. Chu K, Massoud J, Sabbaghian H. Host-parasite relationship of Bulinus truncatus and Schistosoma haematobium in Iran: 3. Effect of water temperature on the ability of miracidia to infect snails. Bulletin of the World Health Organization. 1966;34(1):131.

47. Mangal TD, Paterson S, Fenton A. Predicting the impact of long-term temperature changes on the epidemiology and control of schistosomiasis: a mechanistic model. PLoS one. 2008;3(1):e1438.

48. DeWitt WB. Influence of temperature on penetration of snail hosts by Schistosoma mansoni miracidia. Experimental Parasitology. 1955;4(3):271–6.

49. Coelho JR, Bezerra FS. The effects of temperature change on the infection rate of Biomphalaria glabrata with Schistosoma mansoni. Memórias do Instituto Oswaldo Cruz. 2006;101:223–4.

50. DeWitt WB, others. Effects of temperature on penetration of mice by cercariae of Schistosoma mansoni. American Journal of Tropical Medicine and Hygiene. 1965;14(4):579–80.

51. Briere JF, Pracros P, Le Roux AY, Pierre JS. A novel rate model of temperature-dependent development for arthropods. Environmental Entomology. 1999;28(1):22–9.

52. Van den Driessche P, Watmough J. Reproduction numbers and sub-threshold endemic equilibria for compartmental models of disease transmission. Mathematical biosciences. 2002;180(1–2):29–48.

53. bioclim.html [Internet]. [cited 2024 Jan 24]. Available from: https://www.worldclim.org/data/bioclim.html

54. 1. Jean-Francois Pekel, Andrew Cottam, Noel Gorelick,... - Google Scholar [Internet]. [cited 2024 Mar 12]. Available from: https://scholar.google.com/scholar?hl=en&as_sdt=0%2C5&q=1.%09Jean-Francois+Pekel%2C+Andrew+Cottam%2C+Noel+Gorelick%2C+Alan+S.+Belward%2C+High-resolution+mapping+of+global+surface+water+and+its+long-term+changes.+Nature+540%2C+418-422+%282016%29.+%28doi%3A10.1038%2Fnature20584%29&btnG=

55. Warszawski L, Frieler K, Huber V, Piontek F, Serdeczny O, Zhang X, et al. Center for international earth science information network—ciesin—columbia university.(2016). gridded population of the world, version 4 (gpwv4): Population density. palisades. ny: Nasa socioeconomic data and applications center (sedac). doi: 10. 7927/h4np22dq. Atlas of Environmental Risks Facing China Under Climate Change. 2017;228.
