# Supplementary material for: Re-assessing thermal response of schistosomiasis transmission risk: Evidence for a higher thermal optimum than previously predicted
Source: PLoS Negl Trop Dis. 2024 Jun 10;18(6):e0011836. doi: 10.1371/journal.pntd.0011836 (PMC11207148; doi:10.1371/journal.pntd.0011836)
Supplement: Attachment — Submitted filename: PNTD-D-23-01531.docx [file pntd.0011836.s002.docx]

PNTD-D-23-01531

This paper provides a thorough re-evaluation of the temperature optima for both *Schistosoma mansoni* and *S. haematobium*, with important conclusions being that thermal optima for both parasites look to be higher than previously considered, and that elevated temperature per se seems to be an important factor in modifying human behavior in a way that increases water contact and thus transmission from snails to people. Overall, I am enthusiastic about seeing this paper published.

I’ll confess the mathematical details are beyond me and I must leave it to others to critically evaluate them, but the model description seems logical and appropriate to me and similar to other schistosomiasis models I’ve seen before.

The effort to parameterize the model is laudable. And, yes, this is tricky owing to a dearth of the kind of basic data needed. As a matter of basic snail maintenance and field experience, we can support the idea that the temperature optima of *Bulinus* is greater than *Biomphalaria*. Your figure 2 curves for tolerance of temperature extremes for *Bulinus* and *Biomphalaria* look similar, but there is some reason to believe it is higher for *Bulinus* (at least *B. globosus*) – see the last point below. Practical experience dictates that miracidia are really susceptible to increasing temperatures (longevity sharply declines) as are infected snails.

In lines 362 to 364, it says something to the effect that as temperatures rise, snail egg production drops to zero, and tends to imply this has the effect of increasing R_0_. I was puzzled by this as it seems it would have the opposite effect.

Somewhere near the beginning of the paper, perhaps even in the abstract, it would be helpful to the reader to make a statement relative to what the authors mean exactly when they use the term “temperature”, particularly when defining their optimum ranges? Understood this is a bit slippery in that both air and water temperatures are relevant here, and that temperature is both pervasive and ever changing (but somewhat predictable) in its impacts. In general, there seems to be a scarcity of water temperature data in waterbodies transmitting schisto.

Also quite understood that it is helpful and convenient to express thermal optima simply, in this case as a range of temperatures, and that such values facilitate comparisons with other biological systems and enable schistosomes to be discussed more broadly by climate modelers. What is somewhat harder is for public health people and field workers to understand operationally if the temperature regimes experienced in their local environments are below, at, or above the optima. One concern is that a few simple water temperature measurements might lead to an erroneous conclusion about a particular habitat being too hot or too cool if the measurements fall outside the range.

In response to this kind of concern, the authors have appropriately made note of several important caveats that will influence the outcomes, including temperatures that vary with depth and time of day such that mid-day might be in excess of the range estimates and night-time or deepwater temperatures being below?? How the ranges expressed might relate to more integrated measurements of temperature regimes (degree days or some such?) would be worth exploring.

Although figure 2 is already busy, it would be helpful to the reader if the vertical axis could be labeled not only with the particular parameter being measured but if possible also with a brief verbal descriptor of what the parameter is, so that a lot of back-and-forth looking could be eliminated, making it more reader friendly.

I agree that previously stated optima were too low and unrealistic. The new estimates, higher and near equal for both schistosome species, made me wonder a little bit relative to a classic pattern reported along the East African coast where *S. haematobium* is endemic (transmitted by snails like *Bulinus nasutus* and *B. globosus*), relative to adjacent (cooler) uplands where the snail *Biomphalaria pfeifferi* appears and transmits *S. mansoni* (the bulinids also appear on higher ground too). The thought has been, and lab works seems to support this, that it is simply too hot for *pfeifferi* on these lowland equatorial plains, but the bulinids make it?? I bring this up as a possible way to look more closely to see how your model might handle this situation. Perhaps this is what you are alluding to in your line 365. Maybe there are some cases where a better test for model performance is looking at vector snail distribution data rather than schistosomiasis prevalence. In other words, if there is a demonstrable difference in the upper bounds of what the bulinids and biomphalarids can take, and if it seems to explain a biogeographic pattern in schisto distribution, it would be good if your calculations supported this?? Also, the predictions as to what might happen in future as temperatures rise in equatorial lowland East Africa might be that rather than favoring expansion of both schisto species, it might lead to the elimination of *B. globosus* along the coast (currently living near thermal max) and shift both schistosome species to higher, cooler ground, with real range contractions, especially for *S. mansoni*?? The papers by O’Keeffe on *Bul. globosus* in coastal Kenya are interesting in this regard.

I think it is important to consider the thinking of Mas-Coma on your topic and to cite his review paper, below. He considers effects of increasing temps on both shortening the pre-patent period which you indicate is an important consideration, and also increased mortality rates of prepatent and shedding snails with increased temps, which I note you have considered, but might warrant a second look.

Mas-Coma, S., Valero, M.A. and Bargues, M.D., 2009. Climate change effects on trematodiases, with emphasis on zoonotic fascioliasis and schistosomiasis. *Veterinary parasitology*, *163*(4), pp.264-280.
