# Supplementary material for: Re-assessing thermal response of schistosomiasis transmission risk: Evidence for a higher thermal optimum than previously predicted
Source: PLoS Negl Trop Dis. 2024 Jun 10;18(6):e0011836. doi: 10.1371/journal.pntd.0011836 (PMC11207148; doi:10.1371/journal.pntd.0011836)
Supplement: Attachment — Submitted filename: response_to_the_reviewers.docx [file pntd.0011836.s003.docx]

Dear Dr. Luc E. Coffeng,

I am writing on behalf of all authors of our research paper to express our gratitude for the constructive feedback provided by the reviewers on my manuscript titled "**Re-assessing thermal response of schistosomiasis transmission risk: evidence for a higher thermal optimum than previously predicted**" submitted to *PLOS Neglected Tropical Diseases*. We appreciate the time and effort invested in reviewing our work.

We have carefully considered each comment and suggestion and have made significant revisions to address the concerns raised by the reviewers. There were three major points the reviewers and you asked us to address, namely:

1. Availability of code and data: We have made the code and data publicly available through the link <https://github.com/ibrahimhalilaslan/Thermal_sensitive_schistosomiasis_model>

2. Visualization and label issues in the Figures: We have modified Figures 2 and 3 with their labels to enhance their clarity and interpretability.

3. Unclear and insufficient information in the main text: We have clarified some of the sentences and added some additional pieces of information and discussion to the main text.

We have enclosed below a point-by-point response on how we addressed the reviewers’ feedback and highlight our response in red. These modifications, we believe, have enhanced the overall quality and clarity of the manuscript.

On behalf of all co-authors, I want to thank the reviewers for their insightful suggestions.

We kindly request a reevaluation of the manuscript, and we are hopeful and confident that it now meets the standards for publication in *PLOS Neglected Tropical Diseases*. We appreciate your time and consideration in this matter.

Thank you for your continued support, and I look forward to hearing from you soon.

Sincerely,

Ibrahim Halil Aslan

Postdoctoral scholar

Department of Biology, Stanford University

Contents

[Methods	1](#_Toc1608046386)

[Results	5](#_Toc1264840045)

[Conclusions	7](#_Toc31081488)

[Summary and General Comments	11](#_Toc1499914083)

## **Methods**

Reviewer #1:

Appropriate throughout - no concerns

Reviewer #2:

The objectives of the study are clearly explained in the introduction and the study design is appropriate to address these questions. However, some more details on the methods used for the literature review are welcome that gives the reader an idea of the effort that has been put in this review e.g. the keywords used, search engines, how many studies were retrieved and how many were deemed useful etc.

Thank you for your feedback. We add sentences as follow in L172.

“We used the search engine of ISI Web of Science between Nov 2022 and April 2023 and found 143 papers matching the keywords: (“*temperature” OR "thermal response" OR "thermal sensitivity"*) AND schistosomiasis AND snail. We identified about 50 papers reporting experimental results and used their bibliography and citations to identify further papers that might have not been included in the original search.”

Reviewer #3:

The objective of the study is clear and a relevant contribution to the field. Generally speaking, the methods are appropriate, but I have several issues that should be addressed before publication.

First of all, it is standard practice to make code and data available and reproducible. The code used in the analysis is not available, as far as I can see. The data on life history traits is only available in a large number of tables in the supplementary materials, which is not an easily accessible format. It is entire unclear what data was used to calculate R0 in the locations in the GNTD database (lines 214-221). To rigorously review the methods, the code necessary to reproduce the analysis should be made available. (I understand this may not be entirely possible for the parts of the analysis that involve the GNTD database).

Thank you for your comments. We made the code and data publicly available through the link. <https://github.com/ibrahimhalilaslan/Thermal_sensitive_schistosomiasis_model>

Please note that we use GNTD data to support our results. In L214, we mention how we derived R_0_. “We derived the basic reproduction number $R_{0}$ by using the next generation matrix method ^55,56^ (see SM for detail)..” Recall that we separately found the thermal response curve for each of these parameters as discussed in the parameterization section. We plug these curves into R _0_ and evaluate the value of R _0_ for each temperature.

The fitting of thermal performance of the traits is key to the entire analysis.  The choice for which functions you chose as the best fit could be better argued for. According to the supplementals, the choice for curves was based on 1) AIC values, 2) the number of parameters, and 3) biological applicability. However, you do not always choose the curve with the lowest AIC value, the number of parameters is already accounted for in the AIC, and you could argue better for what biological applicability is. E.g. you might a priori reject certain curves for certain traits.

Thank you for bringing this up and allowing us to provide a better rationale for our selection of model candidate when we decided that the thermal curve with the lowest AIC value was not necessarily the best choice. We have modified the sentence in L182 as “We determined the best-fitting curve for each temperature-dependent parameter out of the family of 24 curves based on the Akaike Information Criterion (AIC), number of parameters, and biological applicability. We overcome AIC criteria when the curve with lowest AIC shows unrealistic trends, like taking negative values in the range of temperatures where schistosomiasis data are available or showing a multimodal behavior near extreme temperatures in schistosomiasis transmission areas.” It is true that AIC already penalized the model for the number of parameters, however, the penalty is a combination of Likelihood of the distance between the true model and estimated model plus the number of parameters used in the estimated model. Due to low sample sizes, most of times we chose models with fewer parameters even where the AIC was not the lowest. This will lead us to pick the model with the lowest bias.

The rTPC package is great, but it has limitations, which you do not discuss. Some widely used curves are not part of the package, such as the Growing Degree Day or linear function. I appreciate that you estimated the uncertainty of the estimates - however, resampling assumes that the observations are independent, which the observations here are not (especially where multiple species are pooled together). An alternative approach would be to use Bayesian inference, which multiple of the coauthors on the paper have used in similar studies in the past.

We agree with the reviewer, and we revised the manuscript accordingly to explicitly acknowledge the limitations of the rTPC package, the most important of which is the lack of monotonic functions (whether increasing or decreasing, linear or non-linear). We added a sentence at L524 “... Another caveat of our study is that the lack of simpler functions within the rTPC package. Most functions used within the rTPC package are quite complex, which does not match well the inherent of LHTs. Using simpler (e.g., monotonic) functions might instead help to narrow the confidence intervals associated with the estimation of thermally sensitive parameters. Thus, packages like rTPC need to be improved with simpler and monotonic functions.”

The reviewer makes also an interesting point about using Bayesian statistics or better resampling/bootstrapping methods. Instead of individual data points, we could bootstrap data by stratifying them by different studies, experiments, or snail species.. However, the data is so sparse that we didn’t really have the choice but to use all the data we had and treat them consistently through all the life history traits and epidemiological parameters. Ideally, one day we will have enough experimental data to estimate thermal tolerance curves for each snail species individually, but we are not quite there yet. Following the approach implemented in other seminal papers (see for instance Mordecai et al. 2013, *Ecology Letters*) we pooled data together at the genus level, and we will leave Bayesian approaches to further developments of our models as we anticipate that they would not change the outcome of the present analysis. We now added a better and more explicit discussion of the potential limitations of our bootstrapping approach in L506, namely:

“The scarcity of species-specific experimental data to describe how temperature affects life history traits and epidemiological parameters of snails and their parasites prevented us to estimate thermal sensitive Ro and prevalence curves for each individual competent snail species. For the same reason, it was not possible to account for potential local adaptation of same-species snails to different climates (Diakité et al. 2023). Combining the species belonging to *Biomphalaria* and *Bulinus*, respectively, thus introducing possible sources of uncertainty or bias in the estimation of genera due to the sparsity of data leads deviation from accuracy in our estimation process. In addition, for any specific model parameter, we have bootstrapped all data available without stratifying them by published study, experiment, or snail species and future analyses should consider the application of more sophisticated Bayesian approaches.”

I appreciate how you go about modelling the water contact rate, i.e. first as a constant and then as temperature dependent.

Thank you for your comment.

Showing the disease-free equilibrium (eq. 11, line 201) may be superfluous. I don't see it used anywhere in the results section.

Thank you for your suggestion. We decided to move it to the supplementary material.

When comparing to the GNTD data, it could be interesting to know if modeled prevalence was a good predictor of observed prevalence. Right now, you only show that R0 is a good predictor of prevalence, but one might expect modeled prevalence would perform even better.

We compared our results to Nguyen’s using R_0_ because their estimation was done with R _0_. We compared our simulation of prevalence with GNTD data in the Figure 3C-D. Our goal was to fit general trends in the data and check whether model projections reflected underlying patterns, but we never intended to precisely estimate prevalence in each location, as the actual value of prevalence results from many other socio-economic, public health, and environmental variables (including access to clean piped water, use of latrines, access to health care, and whether the human population received one or more rounds of mass drug administration, among the others).

Furthermore, you mention that you limit the analysis to locations with non-zero prevalence (line 216). Temperatures might be one of the reasons why schistosomiasis is absent from these locations, so excluding them could clearly affect your results. Please explain more and at least mention how many data points were excluded in this step.

Thank you for bringing up this important detail. There are 11,333 data points for *S. mansoni*, of which 6,250 have non-zero prevalence in humans, and 14,313 *S. haematobium* data points, of which 4,873 have non-zero prevalence. We now include this information in the main text at L231 and write “The 2015 version of the GNTD database included 11,333 *S. mansoni*, of which 6,250 have non-zero prevalence in the human population and 14,313 *S. haematobium,* of which 4,873 have non-zero prevalence, out of nearly 8,000 unique locations.” We decided to remove the zero-prevalence data points as we could not easily distinguish between areas where we would not expect schistosomiasis transmission at all (areas with little presence of, or access to, water), and areas where temperature might be limiting. One of our assumptions we made in model parameterization section is that “.....as our model parameterization assumes that schistosomiasis is present in the population.

## **Results**

Reviewer #1:

Generally, clearly presented - some suggestions made on attached comments

Thank you for your comments, which are specifically addressed at the end of this letter.

Reviewer #2:

Some of the TPCs show some counterintuitive shapes (e.g. the death rate curves in fig 2 C and D) How is it possible that the death rate barely increases with decreasing temperature? Is the tolerance limit of the snails this low that they can survive temperatures lower than 10 degrees and isn’t there any data available for lower temperatures? Anyway it is good to use the inset figures for the parameterization of the model with temperatures under 33 °C

Thank you for your comments and question. The death rate does increase with decreasing temperatures, but that is hard to see because the spike in mortality rate when temperatures approach the upper critical thermal threshold is much sharper and larger than the smooth increase in mortality rate at low as documented by El-Hassan et al. 1974. In other terms, heat is deadlier than cold temperature for snails within the temperature range measured. We just note that this is consistent with the small schistosomiasis outbreak occurred in the Cavu river in the Corsica Island in 2013, as described by Boissier et al. 2016 (Outbreak of urogenital schistosomiasis in Corsica France, an epidemiological case study; The Lancet). Here temperatures might occasionally drop close to, or below, zero °C, and yet snails can overwinter in a dormancy state. We reported here below the data and the interpolating curve on a semi-log scale that we think clarifies the point, so we decided to replace the plots in Figure 2 C and D with these new plots.

|  |  |
| --- | --- |

Reviewer #3:

The results are convincing and for the most part clearly presented.

I am in particular concerned about the fit for the infected snails' mortality rate, panels 2E-F, which seems to fit the data poorly at the high end. I am wondering if fitting to the lifespan (one over the death rate) would give a better fit?

It is a good suggestion, and we tried it, but it didn’t improve the fitting. A few points were missing in the plot visualization for high temperatures. An updated version of the data and the best fitting curve is reported here below.

|  |  |
| --- | --- |

Furthermore, fitting the prepatent period as part of a gaussian function (line 269, panels 2G-H) seems like a curious choice. See my comment above about the growing degree day model.

The reviewer is totally right. As already mentioned in replying to a previous point made by Reviewer 3 above, there we suffer a limitation of rTPC here. The fact that rTPC does not include monotonic functions (in this specific case, an exponentially decreasing function), which would have been the most obvious choice for the duration of the prepatenttime data.The reason we pick Gaussian because of the number of parameters, however, It is counterintuitive to have the optimal temperature at the negative temperatures. Thus, we change the best fit for the prepatent period to Irf curve. We hope this will fix the concern. (As a side note, because the problem is the lack of monotonic functions in rTPC, estimating temperature vs 1/prepatent – in place of temperature vs prepatent time - didn’t solve the problem either).

I find the description of figure 3 unclear. I cannot understand what the larger dots are I presume figure 3C-D show modelled prevalence, but this is not clear from the figure description. In figure 3A-B, the interval goes above 1.0, so I assume you divided the R0 from each sample with the mean max(R0). Wouldn't it be more appropriate to divide the values of each sample with the max(R0) from that particular sample, such that R0/max(R0) is strictly below 1?

Thank you for your suggestion. We added a sentence in the description of Figure 3“ The shaded points in C and D represent all data points from GNTD, while each one of the larger dots in A, B, C, and D represents the 98^th^ percentile of GNTD prevalence data within each bin of 400 sequential temperature points from the GNTD locations.”. We also mention this in the method section at L401.

Thank you for your suggestion. Yes, your assumption is true. If we divide each sample curve with its own max, we will have the plot as below witha flat top which might mislead the readers. The shadow area, representing the 95% credible intervals, inevitably exceeds 1, and we would prefer to keep it as such.

On line 295, I don't really see how a Gaussian curve should have a Tmin and a Tmax, and if it does, these should be symmetrical around the peak.

Thank you for your question. This is a very good catch. We added a sentence to define T_min and T_max in L283 “Note that ($T_{min}$) and ($T_{max}$) are assumed to be the temperature values at which the rate is close to zero.” Yes, Gaussian should be symmetric. We refined our sentence L336 “a Gaussian curve, with its peak at 27.5 ^o^ C, matching the finding in^26^, and $T_{min}$and $T_{max}$ around 10 and 40 ^o^ C, respectively”.

## Conclusions

Reviewer #1:

The conclusions seem valid and are appropriately nuanced by the authors.

Reviewer #2:

Some of the conclusions are a bit too strongly formulated and not fully supported by the data. Especially the conclusion in the abstract on L37: Is there a greater risk of increased transmission with future warming? Were there any projections made in geographic space? What is the statement that there will be a greater risk of increased transmission with future warming based upon? I can imagine this does not hold for all areas, especially those that are already experiencing high temperatures.

Thank you for your feedback. We currently are working on a paper about temperature projections, but we decided not to illustrate actual projections here. The comments we made in L42 are based on the observation that the thermal optimum is actually higher than previously estimated. The problem is that most regions where schistosomiasis is endemic currently have mean annual temperatures below the temperature, we found to be optimal. Our optimal temperature. Therefore, all the regions in which schistosomiasis is already endemic and whose mean annual temperature is below the thermal optimum might experience an increase in transmission risk driven by climate change. To clarify this, we modified the last sentence “… future warming for the regions with mean annual temperature below the optimal temperature”. In addition, we decide to add one more figure shows the regions at which increasing the risk of transmission. The extensive results and discussion are in the main tex. A quantitative assessment of the change in schistosomiasis transmission across Africa under a climate change scenario requires the inclusion of additional factors and will be part of our future work.

Reviewer #3:

The results are discussed well, including the wider context, the limitations of the study, and avenues for further research.

The analysis focuses on one prior estimate of optimal temperatures for schistosomiasis transmission (namely reference 25). It would be good to at least mention other estimates as well, such as by McCreesh & Booth (2014).

Thank you for mentioning this. We mentioned McCreesh & Booth (2014).’s paper in both the introduction and the discussion, L105 and L463, but did not compare our results with theirs directly because they did not use R_0_ as a metric.

At the very end of the study (line 420) you mention several countries in Southeast Africa. However, it is unclear to me how you reach this conclusion, as this is the first time in the entire study that your results are discussed in geographic space.

Thanks for your question. We decided to extend our conclusion to the entire Africa. Our conclusion is simply based on countries having mean annual temperatures that are lower or higher than the optimal temperature we found. Please revisit the main text for the extensive simulation of results and discussion.

## **Editorial and Data Presentation Modifications**

Use this section for editorial suggestions as well as relatively minor modifications of existing data that would enhance clarity. If the only modifications needed are minor and/or editorial, you may wish to recommend “Minor Revision” or “Accept”.

Reviewer #1:

A few questions have been posed and a couple of modifications to reduce the amount of cross-checking the reader must do have been made.

Thank you for your suggestions. They are addressed at the end of this response.

Reviewer #2:

Introduction:

L57-60: restructure this sentence a bit to the end of the discussion (“we use a mechanistic, process… on the transmission risk of schistosomiasis”) and the next paragraph on schistosomiasis (“a water-associated parasitic disease affecting more than two hundred million people worldwide (not new infections per year!), the vast majority living in SSA”)

We modified the paragraph as follows: L61 “Many of these ectotherm organisms are involved in the transmission of environmentally mediated diseases in humans, making transmission a temperature-dependent process. Schistosomiasis is one of these environmentally mediated diseases. It is a water-associated, acute and chronic parasitic disease of poverty, infecting more than two hundred million people worldwide, the vast majority of whom are in Sub-Saharan Africa (SSA)”.

L64-66: this is quite a complex sentence, can it be subdivided into smaller sentences?

Yes, we have modified the sentence for clarity.

L67: eggs only hatch upon freshwater contact

We have now modified the sentence accordingly.

L73: this sentence is not completely correct. Now it reads as if people contact the water by burrowing through their skin.

We replaced the sentence as follows: in L73 “…… A few weeks after infection, depending upon temperature, snails start shedding a second free-living larval stage of the Schistosoma parasite, called cercaria, which infects humans by penetrating through the skin”

L75: Is there no data on cercariae infectivity or the incubation period of miracidia?

Thank you for your feedbacks, We have empirical studies regarding the incubation period of snails and also infectivity of cercaria, however, it seems we were not clear enough in the previous version main tex. We changed our sentences in L79-82, “….Effects of temperature have been reported for the survival and infectivity of cercariae and miracidia ^16–25^, mortality rate of cercariae ^18,26^, miracidia hatching, and cercariae emergence ^27^, snail fecundity, growth, mortality ^10,28–30,30–34^, and parasite incubation period within the snail ^15,21,35,36^, demonstrating pervasive effects of temperature across the schistosome life cycle. While these experiments provide insight on the response of individual LHTs…” The reference shown are the empirical study where we collect the data.

L89-92 which temperature-dependent LHTs have been omitted in papers 37 and 39?

The prepatent period in snails, hatching, and maturation of snails are omitted in papers 37 and 39. We refined the sentence L89-92“… which is strongly correlated with temperature; furthermore, the temperature dependence of snail egg production, hatching, and survival rate have not previously been incorporated.”

Materials and methods
L149 move the definition of sigma higher so all parameters are explained under the equation that first mentions them.

we moved the definition to L158.

L158 Could the authors provide some more details on the methods for the literature review? E.g. the keywords used, search engines, how many studies were retrieved and how many were deemed useful etc.

We add the sentence in L172.

“We used the search engine of ISI Web of Science between Nov 2022 and April 2023 and found 143 papers matching the keywords: (“temperature” OR "thermal response" OR "thermal sensitivity") AND schistosomiasis AND snail. We identified about 50 papers reporting experimental results and used their bibliography and citations to identify further papers that might have not been included in the original search.”

L175 MPB has not been defined yet (I assume that MPB is the mean parasite burden in the populations, i.e. the prevalence in the populations?). Additionally, is there any data available on the mean number of parasites in a single human host? I might have missed it but it seems that this parameter is missing although the number of eggs produced per worm inside the human body is present in the equations (ve in equation 1). Or does the model consider only one parasite per human? If so mention this explicitly.

Thank you for alerting us for forgetting the abbreviation. Yes, MPB refers to mean parasite burden. Note that MPB is related but not equivalent to prevalence, which is typically defined as the fraction of infected individuals within a population. As for your question about data availability on the mean number of parasites in a single human host, we recognize that the relationship between MPB (the key state variable of any schistosomiasis dynamics model since the publication of the first McDonald model in the 1960s ) and egg count in urine (for *S. haematobium*) or in feces (for *S. mansoni*) is tricky, as the only observable variable is the egg count, but the state variable in the model is the mean parasite burden. Information on paired female worm fecundity can be derived only from limited studies on animal models. We specifically referred to Cheever et al. 1994 ("Kinetics of egg production and egg excretion by *Schistosoma mansoni* and *S.* j*aponicum* in mice infected with a single pair of worms", American Journal of Tropical Medicine and Hygiene). Although the per capita worm fecundity is considered to be extremely variable, there is a general consensus that *S. mansoni* pairs can lay approximately 350 eggs per day, and that half of the eggs produced daily are eventually released through urine (*S. haematobium*) or feces (*S. mansoni*). If we assume that a school-age child (SAC) produces around 1.25 liters of urine/day (Rose et al. 2015, The Characterization of Feces and Urine: A Review of the Literature to Inform Advanced Treatment Technology), the concentration of eggs in 10 ml of urine for every worm pair is about 350*0.5 *10/1250 = 1.4 eggs per worm pair/day/infected SAC. Therefore, in our model a mean parasite burden of 80 worm/SAC is equivalent, on average, to an egg count of ca. 56 eggs per 10ml of urine, which is well in line with the range of empirical data on egg counts from our field sites in Senegal (Sokolow et al. 2015, Rohr et al. 2023) and the simulation output from other well-established modelling papers (Truscott et al. 2017, Fig. 3C, D).

To derive the prevalence of infection associated with a given MPB, we then assumed a negative binomial distribution of parasites (and eggs) in the human population, with clumping parameter increasing with mean parasite burden. This reflects the observation that worms are clustered in fewer human subjects when MPB is low (e.i. clumping parameter < 1), whereas the worm distribution tends to a Poisson distribution when MPB is high (e.i. clumping parameter approximately equal to or above 1) . Given the MPB, we derived the clumping parameter and then, following May 1977 (“Togetherness among schistosomes its effects on the dynamics of infection”), the prevalence of infection in the population (i.e., the probability of number of human subjects with at least one pair of reproductive parasites).

Results
L261 to 267 specify in the text that no optimum for survival could be determined. Otherwise, it is a bit strange to report it for the healthy snails but not for infected snails as this would be a really interesting comparison.

We agree with the reviewer, it would be very interesting. Unfortunately, the data on the mortality rate for infected snails are scarce and, as a result, our exercise of thermal function interpolation does not yield a clear thermal optimum (the temperature that minimizes the mortality of infected snails). Accordingly, we add the following sentence at L305: “Note that it was not possible to determine the temperature minimizing the mortality rate of infected snails due to the strong noise in the data, particularly for *Biomphalaria* spp. This also leads to a wider confidence interval at higher temperatures (Figure S12B-S13B).”  

L 305 what is meant by 3.68 and 3.44 peak value? The curve only goes until 1.

Figure 3A-B show normalized values. However, non-normalized results are shown in the supplementary material. We revised the sentence in the main text at L389: “...unimodal concave shape for both *S. mansoni* and *S. haematobium*, peaking at 25.5 ^o^C (CI: 23.1-27.3 ^o^C) and 26.2 ^o^C (CI: 23.6-27.9 ^o^C) with 3.68 and 3.44 peak values (Figure 17SA-B, SM), respectively .

Discussion
L348 the authors didn’t look exclusively at thermal tolerance but also at thermal optima.

We added this in discussion at L358. “…on the thermal tolerance and optima for the LHTs of the parasites *S. mansoni* and *S. haematobium* and their associated snail hosts, which in this context had not been studied separately yet. …

Conclusion
L420 Zambia instead of Zombie?

Fixed.

Reviewer #3:

Table 1 would be clearer if the first column were split in two: one with the symbols used for the parameters and one with the description of what the parameter is, just as in Table 1 of the supplementals.

Thank you for your suggestions. We modified the table as you described.

There are several minor typos in throughout the text. I will mention some:
- The abbreviation MPB is used several times but never written in full (fixed)
- Line 306, I think 24 C should be 24.0 C. If this is a mistake it occurs several times in the text (fixed)
- Line 329, I think the word 'the' is missing (fixed)
- Line 330 and 331, 'reachs' should be 'reaches' (fixed)
- Line 362, 'reproduction' should be 'production'. (fixed)
- Line 377 'Snails' should be 'snails' (fixed)
- Line 100, my guess is 'to a higher degree' should mean 'to a higher temperature'(fixed)
- In the supplementary materials, on page 65, some equations are missing (fixed)

## Summary and General Comments

Use this section to provide overall comments, discuss strengths/weaknesses of the study, novelty, significance, general execution and scholarship. You may also include additional comments for the author, including concerns about dual publication, research ethics, or publication ethics. If requesting major revision, please articulate the new experiments that are needed.

Reviewer #1:

In general, I am enthusiastic about seeing this paper published as it pulls together a huge amount of information to come up with more realistic estimates for temperature optima for schistosomiasis transmission for the 2 most important schistosome species.

We appreciate your enthusiasm for our paper and thank you for your comments that greatly improved the manuscript.
See attached comments

Reviewer #2:

This paper investigates the thermal optimum for schistosomiasis transmission using a mechanistic modelling approach parameterised by experimental life history data obtained through a literature review. This is undoubtedly a valuable effort for schistosomiasis research and risk assessments and investigations on the effects of climate change on schistosomiasis transmission. The work done is of high quality despite the somewhat limited and outdated data available to parameterise the models. As the authors highlight themselves, more recent and reliable data on both snails and parasites is needed. Related to this data deficiency is the main limitation of this study: the authors used data on different snail species to generalise findings across snail species. It would have been more interesting to run the model per snail species as the species differ between regions, which might result in different optimal temperatures for schistosomiasis transmission. These presumptive differences could result in variable forecasts in schistosomiasis transmission under climate change in different areas. Therefore, I believe that some of the statements made on future schistosomiasis transmission are a bit too strongly formulated as it is not sure that climate change will increase transmission in all areas. Projection of the model results to geographic space could already give a better idea of how transmission dynamics could change in the future under different climate change scenarios in different areas.

Thank you for your thoughtful review and comments. We do agree with you that species-specific models would be preferable and more precise. Even when we focus on a specific species, we could not rule out the potential for local adaptation to climate change, in that populations of snails from different climatic regions might exhibit locally climate-adapted life-history traits. Unfortunately, species-specific data are so scarce and scattered that we were left with no other alternative than pooling together data for same-genus snail species (i.e., *Biomphalaria* and *Bulinus,* respectively). We agree with the reviewer that this is a limitation of our study and, therefore, we have openly and directly discussed it in the corresponding section of the discussion, where we write (in L506-L510): “The scarcity of species-specific experimental data to describe how temperature affects LHTs, epidemiological parameters and their parasites prevented us to estimate species-specific thermal response curve of $R_{0}$ and the LHTs curves. To offset the paucity of data over a large thermal breadth, we had to pool together data from snails belonging to the same genus but to different species. Combining the species belonging to the genera *Biomphalaria* and *Bulinus*, respectively, introduces possible sources of uncertainty and bias in the estimation accuracy due to the sparsity of data.”. However, species-specific data are so scarce and so scattered that it would have been impossible to estimate thermal sensitive model parameters for each relevant snail species. Therefore, following previous studies (Mordecai et al. 2013, Shocket et al. 2018), we grouped together data for different snail species by taxa/genus, i.e., either *Biomphalaria* or *Bulinus* genera”. In addition, we also modified some sentences (L506-L510) to soften the language and the take-home message.

Reviewer #3:

This paper is a really nice contribution to the field that advances our understanding of the effect of temperature on schistosomiasis transmission.

The schistosomiasis transmission model is well designed and the analysis is complete.

I am requesting major revision because the code and data are not available (in an accessible format).

A better discussion of the limitations of the methods used to fit thermal performance curves would improve the paper, and possibly some further analysis on some of the traits would as well. I find figure 3 unclear, and at the very least the caption should explain the figure better.

Some further copy editing is also needed.

We thank you for your thorough review and for your comments. We have modified the manuscript based on your comments.

Attached (Reviewer #1)

PNTD-D-23-01531

This paper provides a thorough re-evaluation of the temperature optima for both *Schistosoma mansoni* and *S. haematobium*, with important conclusions being that thermal optima for both parasites look to be higher than previously considered, and that elevated temperature per se seems to be an important factor in modifying human behavior in a way that increases water contact and thus transmission from snails to people. Overall, I am enthusiastic about seeing this paper published.

I’ll confess the mathematical details are beyond me and I must leave it to others to critically evaluate them, but the model description seems logical and appropriate to me and similar to other schistosomiasis models I’ve seen before.

The effort to parameterize the model is laudable. And, yes, this is tricky owing to a dearth of the kind of basic data needed. As a matter of basic snail maintenance and field experience, we can support the idea that the temperature optima of *Bulinus* is greater than *Biomphalaria*. Your figure 2 curves for tolerance of temperature extremes for *Bulinus* and *Biomphalaria* look similar, but there is some reason to believe it is higher for *Bulinus* (at least *B. globosus*) – see the last point below. Practical experience dictates that miracidia are really susceptible to increasing temperatures (longevity sharply declines) as are infected snails.

Thank you for your comments. Our findings are indeed consistent with this reviewer’s experience and intuition. Our projected thermal optimum for R_0_ for *S. haematobium* is 0.7 °C higher than *S. mansoni* (Fig. 3A and 3B). The upper critical thermal threshold for *S. haematobium* (Fig. 3D) is also significantly higher than that for *S. mansoni* (Fig. 3C) in line with the reviewer’s knowledge. We know that we have to take these results with a grain of salt, given the paucity of species-specific data, especially for *Bulinus* snails and *S. haematobium* parasites, but we are happy that our projections are consistent with the reviewer’s expectation. Also, we do agree with the general statement that miracidia longevity decreases when temperatures exceed the thermal optimum, as shown in Figure 2 I, J, is in agreement with the metabolic theory of ecology.

In lines 362 to 364, it says something to the effect that as temperatures rise, snail egg production drops to zero, and tends to imply this has the effect of increasing R_0_. I was puzzled by this as it seems it would have the opposite effect .

We have rewritten these statements for improving clarity (L482): “we found multiple empirical studies ^28,29,68^ on the thermal response of egg production by snails, egg hatching rate and hatching time, survival and maturation rates of immature snails, which had not previously been incorporated into models. Increasing temperatures increase the fraction of snail eggs that hatch and reduce the hatching time, thus increasing the pace of transmission, and contributing to further shifting the thermal optimum of prevalence in humans and $R_{0}$ toward higher temperatures. At some point, however, the benefits for parasite transmission derived from increasing snail and parasite hatching rates at increasing temperatures are more than compensated by the costs due to the exponential increase in snail mortality at temperatures approaching or exceeding 30 °C, thus leading to a sharp drop in $R_{0}$ in correspondence of the upper thermal threshold.”

Somewhere near the beginning of the paper, perhaps even in the abstract, it would be helpful to the reader to make a statement relative to what the authors mean exactly when they use the term “temperature”, particularly when defining their optimum ranges? Understood this is a bit slippery in that both air and water temperatures are relevant here, and that temperature is both pervasive and ever changing (but somewhat predictable) in its impacts. In general, there seems to be a scarcity of water temperature data in waterbodies transmitting schistos.

Thank you for your suggestion. We always mean water temperature in our study because all experiments (as well as transmission in the field) are happening in waterbodies. We added a sentence at L243: “Note that temperature is a shortcut for water temperature in our study and assume that the waterbody temperature is equal to the mean annual temperature  in the region.” We agree that this is a limitation for these models.

Also quite understood that it is helpful and convenient to express thermal optima simply, in this case as a range of temperatures, and that such values facilitate comparisons with other biological systems and enable schistosomes to be discussed more broadly by climate modelers. What is somewhat harder is for public health people and field workers to understand operationally if the temperature regimes experienced in their local environments are below, at, or above the optima. One concern is that a few simple water temperature measurements might lead to an erroneous conclusion about a particular habitat being too hot or too cool if the measurements fall outside the range.

In response to this kind of concern, the authors have appropriately made note of several important caveats that will influence the outcomes, including temperatures that vary with depth and time of day such that mid-day might be in excess of the range estimates and night-time or deep water temperatures being below?? How the ranges expressed might relate to more integrated measurements of temperature regimes (degree days or some such?) would be worth exploring.

This is a good suggestion. We added the following sentence at L504 in the discussion section.

“In addition, temperature might affect the dynamics of submerged vegetation where competent snails thrive, which in turn might affect snail abundance and reproductive rates in ways that are not accounted for in our model. Moreover, the thermal response in this study has been described with reference to average water temperatures which also assume to be equal to mean annual temperature. Yet, water temperature is influenced by multiple factors, including depth, geographic location, seasonality, and time of day. Consequently, our estimates may deviate from observed prevalence of infection in a specific location.”

Although figure 2 is already busy, it would be helpful to the reader if the vertical axis could be labeled not only with the particular parameter being measured but if possible also with a brief verbal descriptor of what the parameter is, so that a lot of back-and-forth looking could be eliminated, making it more reader friendly.

Thank you for your suggestion. We tried several ways to express the parameters on the y-axis. However, since the description of each parameter is long, and the figures are small, full labels are not legible and using different description may cause confusion. Therefore, we now include the description of the relevant parameters in the legend of Figure 2.

I agree that previously stated optima were too low and unrealistic. The new estimates, higher and near equal for both schistosome species, made me wonder a little bit relative to a classic pattern reported along the East African coast where *S. haematobium* is endemic (transmitted by snails like *Bulinus nasutus* and *B. globosus*), relative to adjacent (cooler) uplands where the snail *Biomphalaria pfeifferi* appears and transmits *S. mansoni* (the bulinids also appear on higher ground too). The thought has been, and lab works seems to support this, that it is simply too hot for *pfeifferi* on these lowland equatorial plains, but the bulinids make it?? I bring this up as a possible way to look more closely to see how your model might handle this situation. Perhaps this is what you are alluding to in your line 365. Maybe there are some cases where a better test for model performance is looking at vector snail distribution data rather than schistosomiasis prevalence. In other words, if there is a demonstrable difference in the upper bounds of what the bulinids and biomphalarids can take, and if it seems to explain a biogeographic pattern in schisto distribution, it would be good if your calculations supported this?? Also, the predictions as to what might happen in future as temperatures rise in equatorial lowland East Africa might be that rather than favoring expansion of both schisto species, it might lead to the elimination of *B. globosus* along the coast (currently living near thermal max) and shift both schistosome species to higher, cooler ground, with real range contractions, especially for *S. mansoni*?? The papers by O’Keeffe on *Bul. globosus* in coastal Kenya are interesting in this regard.

Thank you for your extensive comments and evaluation. Yes, our analysis showed that *S. haematobium* has a higher optimal temperature than *S. mansoni* and, also, that the upper critical thermal threshold for *S. haematobium* is higher than that for *S. mansoni*, in agreement with the reviewer’s knowledge and the available field observations. A temperature increase will generally favor *S. haematobium* as it seems to be able to better persist both at lower and higher temperature, as reflected by its larger thermal breadths. Whether transmission risk will increase with temperature is likely to be location-specific, as it depends on where the current temperature is placed on the thermal tolerance curve: if the temperature in a given location is below the thermal optimum, then higher temperatures have the potential to increase transmission risk. If, instead, the local temperature is close to the thermal optimum or above it, an increase in temperatures may lead to a potential reduction in the transmission rate. Thus, we believe that our calculations would support this reviewer’s inferences. We have not done future projection of schistosomiasis yet, but our addition simulation also compatible with the reviewer inference. Our future work will be to investigate projections of schistosomiasis dynamics under climate change scenarios.

I think it is important to consider the thinking of Mas-Coma on your topic and to cite his review paper, below. He considers effects of increasing temps on both shortening the pre-patent period which you indicate is an important consideration, and also increased mortality rates of prepatent and shedding snails with increased temps, which I note you have considered, but might warrant a second look.

Mas-Coma, S., Valero, M.A. and Bargues, M.D., 2009. Climate change effects on trematodiases, with emphasis on zoonotic fascioliasis and schistosomiasis. *Veterinary parasitology*, *163*(4), pp.264-280.

Thank you for bringing Mas-Coma’s important paper to our attention. We now include it in both the introduction and the discussion.
