# Supplementary material for: Re-assessing thermal response of schistosomiasis transmission risk: Evidence for a higher thermal optimum than previously predicted
Source: PLoS Negl Trop Dis. 2024 Jun 10;18(6):e0011836. doi: 10.1371/journal.pntd.0011836 (PMC11207148; doi:10.1371/journal.pntd.0011836)
Supplement: Attachment — Submitted filename: response_to_the_reviewer.docx [file pntd.0011836.s004.docx]

Dear Dr. Luc E. Coffeng,

On behalf of all the authors, I would like to express our sincere gratitude for the constructive feedback provided by the reviewer in the second round of reviews for our manuscript titled "**Re-assessing Thermal Response of Schistosomiasis Transmission Risk: Evidence for a Higher Thermal Optimum than Previously Predicted**," submitted to PLOS Neglected Tropical Diseases. We greatly appreciate the time and effort invested in reviewing our work.

We have carefully considered each comment and suggestion and have made significant revisions to address the concerns raised by the reviewer. There were a few points the reviewers and you asked us to address, namely:

1. We have corrected minor inconsistencies in the text and R code, which do not affect our results.
2. We have corrected some incorrect notations and misplaced paragraphs.
3. The reviewer made some suggestions regarding the presentation of our results. We have implemented those we found most effective.

We have enclosed below a point-by-point response on how we addressed the reviewers’ feedback and highlight our response in red. These modifications, we believe, have enhanced the overall quality and clarity of the manuscript.

I want to thank the reviewers for their insightful suggestions.

We kindly request a reevaluation of the manuscript, and we are hopeful and confident that it now meets the standards for publication in *PLOS Neglected Tropical Diseases*. We appreciate your time and consideration in this matter.

Thank you for your continued support, and I look forward to hearing from you soon.

Sincerely,

Ibrahim Halil Aslan

Postdoctoral scholar

Department of Biology, Stanford University

Contents

Methods	2

Results	2

Conclusion	3

Editorial and Data Presentation Modifications	4

Summary and General Comments	4

## **Methods**

Reviewer #3: Thank you for addressing the points raised in the first round - in particular for making your code available.

I just stumbled over an odd thing in your code: in some scripts, bins are 300 and in others 400, while in some scripts, you take the 95th percentile and in others the 98th. (this github search shows it nicely: <https://github.com/search?q=repo%3Aibrahimhalilaslan%2FThermal_sensitive_schistosomiasis_model+%22bins_maker%28%22&type=code)>

Otherwise I have no further comments on the methods, I think they are entirely satisfactory.

Thank you so much for highlight the mistake which also caused an inconsistency in the main text. We changed the 300 to 400 and 95^th^ to 98^th^ in the R code file and then replotted $R_{0}$and added these new plots into Figure 3 of the main text again.

## Results

Reviewer #3: The authors have made some important improvements to the results and discussion compared to the first version. I also thank the authors for responding to my questions even if the text was not changed. I have a few points, mainly about some of the text that has been added since the first version.

The sentence you added on line 290: "Note that (T_min) and (T_max) are assumed to be the temperature values at which the rate is close to zero". What is close to zero? I tried to look for this in the code but couldn't find it. For Bulinus fecundity (panel B), you report the Tmin as 10 degrees in the text, but your plot really doesn't show anything that indicates a critical minimum there. In my view, if a curve doesn't have a clear thermal minimum, there's no need to report one. (As an aside, critical thermal minima/maxima are typically abbreviated CTmin/CTmax).

We agree that we need to use CTmin/CTmax in place of T_min and T_max and thank you for pointing it out. Note that, the newly derived thermal sensitive $R_{0}$ has a much wider thermal breadth than the thermal sensitive $R_{0}$ previously derived by Nguyen et al. and, in addition it matches the thermal breadth of the GNTD data much better than the previous one. However, CTmin/CTmax cannot be simply derived from the relative $R_{0}$ (due to re-scaled to compare previous estimate), So, we used CTmin/CTmax of curve of prevalence to determine the thermal breadth. On the other side, we agree that we do not calculate CTmin/CTmax for most of the LHTs curve, however, we think it is useful to inform the reader about thermal breadths and we hope that the reviewer considers acceptable if we rephrase the wording in the main text as “a rough approximation of thermal breadth (14, 33 °C)”. in here and all other places.

I now understand figure 3, thanks for the clarifications. I understand that the flat top you get by dividing each replicate could be misleading. However, I think presenting it in the way you do here also has some downsides, namely 1) allowing R0/max(R0) to be above 1 is also confusing; 2) you lose the biological meaning of R0 by dividing with the max; and 3) showing points with prevalence relative to the maximum observed prevalence is confusing, especially if the axis is labelled R0/max(R0). I would propose you instead use 2 y-axes, with the left y-axis just R0 (not normalized - I understand one of the new/previous estimates curves would be higher than the other, but that is also a result), and the right y-axis prevalence. You could add a horizontal line at R0=1 to indicate the thermal limits of transmission, i.e. the temperature range between which both models predict stable transmission is possible.

We think that the reviewer makes a series of good points. Accordingly, we have updated the figure with two axis, $R_{0}$ on the left (without units, as in Mordecai et al. 2013,. Fig. 2) and prevalence data on the right axis. In the Figure legend, we added: The y-axis for R0 is not scaled because its absolute value depends on other climatic and socio-economic factors.

It's also not entirely clear to me why you show the 98th percentile of prevalence of each bin in the figure, while the result you report in the text uses the median. This looks even more odd because there seems to be some data articact that means prevalence is capped at 50% (I understand there is nothing you can do about this). Finally, if I understand this figure correctly, the dots in figures A and C should match, but they don't seem to (the dots in figures B and D do match).

Thank you for asking for further clarification. We use the 98^th^ percentile of data in each bin for visual comparison of our results with previous estimate. (we choose 98^th^ because our $R_{0}$ curve looks a good fit with GNTD data up to 98^th^). we run quantile regression for quantitative compassion of our result with previous estimate. We conclude that *o*ur estimate of $R_{0}$ is a significant predictor of prevalence for all quantiles *(*$\tau=$*0.25,0.5,0.75, 0.9, 0.95, 0.98, p-value < 0.005)* while the previous estimation was not a significant predictor of prevalence beyond the 25th quantile. We did not use the median in our analysis. I believe the word median might be confusing in the text. Thus, we reword our sentence in the result section “*o*ur estimate of $R_{0}$ is a significant predictor of prevalence for all quantiles *(*$\tau=$*0.25,0.5,0.75, 0.9, 0.95, 0.98, p-value < 0.005)* while the previous was not a significant predictor of prevalence beyond the 25th quantile.”

I think the map of Africa is a nice addition, and it is much clearer where the climate data comes from. The jump from annual mean air temperature to observed thermal responses in the lab is big, but saying anything about water temperature as experienced by snails is very difficult, so I think that that is fine for the purposes of this paper. Plotting both R0 and its derivative w.r.t. temperature is clever. One suggestion I have is to use a color scale with a break at R0 = 1, so that your map also shows in which areas your model predicts stable transmission is (not) possible.

Thanks a lot for your supportive comments. We agree that the relationship between air temperature and water temperature is very tricky and, depending upon the size of the water body, can go in very different directions. As for the heatmap, we have updated the figure as suggested, we hope that it is much more clear now where transmission risk is high (red areas), where it is low (yellow areas, where transmission is less likely) and where transmission is simply not possible (white regions) as there are no nearby waterbodies that are either permanent or last at least 5 months or longer.

As mentioned above, since $R_{0}$ depends on several socio- economic variables and it is highly regional, it is hard to know the magnitude of $R_{0}$. if we use the quantity of our $R_{0}$ estimation which ignores many other variables, we will give some extra wrong information

You also added a paragraph in the discussion on lines 465-470, where you introduce numbers that are not in the results or methods. I think you can have two options here: either remove these lines and keep a more thorough spatial analysis in the paper you mention you are working on, or move these to the Results section and make it clearer what analysis you actually performed. In particular, I think you jump too quickly from the derivative (below/above the thermal optimum) to increasing/decreasing with climate change. A derivate of 0 does not mean the expected change due to climate change would be 'minimal', because the degree of climate change matters. With extreme climate change, even areas with a dR0/dT above 0 could eventually see decreased transmission. You could also add confidence intervals to all numbers you mention based on the bootstrap to show the robustness of these results.

The reviewer makes a good point, we moved these lines in the Result session and acknowledged that further simulations will be necessary to assess where and how much transmission risk might change under different climate change, namely:

”Further analyses also show that ca. 62.7% of the areas where temperatures are currently suitable for urogenital schistosomiasis transmission are below the thermal optimum and, thus, a small increase in temperature, as pre-dicted by climate change, might lead to an increase in transmission risk (56.2% for intestinal schistosomiasis). Ap-proximately 29.3% of these areas are above the thermal optimum and thus, a temperature increase would lead to a reduction in transmission risk for urogenital schistosomiasis (34.7% for intestinal schistosomiasis), while around 8% of the areas (9.1% for intestinal schistosomiasis) are close to the thermal optimum and, thus, small changes in tem-perature are expected to have a negligible effect of on transmission risk. Areas close to the thermal optimum might still experience a decrease in transmission risk if temperatures increase substantially. Ultimately, further simulations of transmission risk will be necessary to precisely assess where and how much transmission will change under climate change scenarios. “ >>

## Conclusion

Reviewer #3: The conclusions are clear and well backed up by the results presented.

Thank you.

## Editorial and Data Presentation Modifications

Reviewer #3: All references seem to be off by 1, at least halfway through the text. Maybe you just forgot to refresh the reference manager?

We fixed the reference list.

On like 315, you mention the "Irf hyperbolic function". I think this what is meant here is the Lobry-Rosso-Flandros (LRF) model - which however does not seem to be hyperbolic. I can't find any reference to a "Irf hyperbolic function" anywhere else.

Yes, we are referring to the Lobry-Rosso-Flandros curve and you are right, it is not hyperbolic. We meant it has multiple inflection points. We decided to remove the word hyperbolic in the text.

Is the GNTD dataset publicly available? The link in your references leads to a page which asks for a login.

Sadly, curator is not currently making the GNTD data set publicly available. So, one needs to contact the owner of data set. Dr. Penelope Vounatsou, Swiss TPH, email: penelope.vounatsou@swisstph.ch ,who is properly mentioned in the acknowledgement

Finally, I would take one more look at the very first sentence of this paper. You mention the climate has warmed by 0.6 degrees - I think this comes from the Arnell paper, but that compares 1981-2010 with pre-industrial. Most datasets show 1.1 or 1.2 degrees of warming to date. You also cite the third IPCC report from 2001 (why not the latest?), and "some climate change scenarios project even faster increases in the future" sounds a bit weak. I know these are details, but a powerful first sentence could improve the entire paper.

Thank you for rising this point. We followed your suggestion by changing the 1.5^o^C rise and add IPCC recent report.

## Summary and General Comments

Reviewer #3: I appreciate the detailed responses to the points raised in the first round of review. The clarity of the paper has been much improved and the discussion sections is much more complete. Most of my concerns have been addressed. Almost all of my remaining suggestions are about the way data is presented and do not require major modifications to the paper.

Thank you for your valuable inputs.
